# Modular Polymers With Tuneable Charge and Amphiphilicity for Effective Antimicrobial Photodynamic Therapy

**DOI:** 10.1002/marc.70426

**Published:** 2026-09-10

**Authors:** Ali Balasini, Jannik Fryn, Margaux Bonnardot, Jérémy Godard, Tristan Montier, Gilles Lemercier, Franck Thétiot, Tony Le Gall, Ulrich Jonas

**Affiliations:** ^1^ Macromolecular Chemistry Department of Chemistry and Biology University of Siegen Siegen Germany; ^2^ Univ Brest Inserm EFS UMR1078 GGB F‐29200 Brest France; ^3^ ITODYS‐UMR CNRS 7086 Univ. Paris Cité Paris France; ^4^ CHU de Brest Service de Génétique Médicale et Biologie de la Reproduction Centre de Référence des Maladies Rares "Maladies Neuromusculaires" Brest France; ^5^ Université de Reims Champagne‐Ardenne Reims France; ^6^ CNRS UMR 6521 CEMCA Univ. Brest Brest France

**Keywords:** antimicrobial polymers, photodynamic therapy, polymer–metal hybrids, post‐polymerisation modification, ruthenium complexes, structure‐activity relationships

## Abstract

A modular synthetic platform delivers polymers that integrate, within a single architecture, a hydrophilic backbone, hydrophobic side chains, charged groups, and photoactive agents for antimicrobial photodynamic therapy (aPDT). Ruthenium complexes are used as representative photoactive systems, and clinical strains of *Pseudomonas aeruginosa*, *Staphylococcus aureus*, and *Escherichia coli* serve as bacterial targets. Two complementary strategies are deployed in parallel within a single design framework: free‐radical copolymerisation of acrylic ruthenium–photosensitiser monomers, and post‐polymerisation modification of a pentafluorophenyl‐acrylate precursor with amine‐functionalised ruthenium complexes. Both routes allow independent tuning of composition, solubility, amphiphilic balance, and net charge, a set of properties that together govern the polymers' interaction with bacterial surfaces. The approach yields a library of seven water‐dispersible, amphiphilic polymer–metal hybrids. Benchmarked against a bare ruthenium complex, the aPDT‐polymers exhibit strong UV–visible absorption, efficient reactive oxygen species production, low (photo)cytotoxicity towards human bronchial epithelial cells, and potent antibacterial activity, particularly against multidrug‐resistant *P. aeruginosa* in planktonic and biofilm states. Multivariate clustering reveals clear guidelines for structure–activity relationships: anionic acrylic‐acid‐rich architectures are most effective against *P. aeruginosa* biofilms under saline conditions, while cationic compositions are preferential against planktonic *S. aureus*. The platform thus provides a rationally tuneable basis for next‐generation polymer‐based photoactive antimicrobials.

## Graphical abstract

A modular dual‐strategy platform combining free radical copolymerisation and post‐polymerisation modification yields a library of ruthenium‐functionalised polymers for antimicrobial photodynamic therapy. Tuneable composition and net charge govern activity against multidrug‐resistant *Pseudomonas aeruginosa* biofilms and *Staphylococcus aureus* under physiologically relevant conditions, establishing structure‐activity relationships for rational design of next‐generation photoactive antimicrobial polymers.

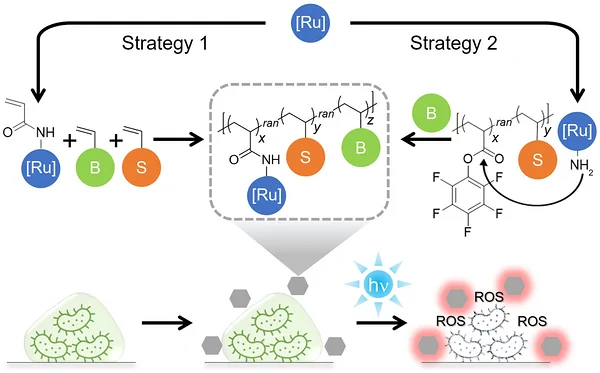

**Abbreviations**
^1^O_2_
: singlet oxygen
AA: acrylic acid
Apdt: antimicrobial photodynamic therapy
ASM: artificial sputum medium
CFU: colony‐forming unit
DMSO: dimethyl sulfoxide
DNA: deoxyribonucleic acid
EC: *Escherichia coli*
FRP: free‐radical (co)polymerisation
GPC: gel permeation chromatography
HEAAm: *N*‐hydroxyethyl acrylamide
LED: light‐emitting diode
MIC: minimum inhibitory concentration
MRSA: methicillin‐resistant *Staphylococcus aureus*
NMR: nuclear magnetic resonance
PA: *Pseudomonas aeruginosa*
PDT: photodynamic therapy
PFP: pentafluorophenyl
PFPA: pentafluorophenyl‐acrylate
PPM: post‐polymerisation modification
PS: photosensitiser
RFU: relative fluorescence unit
ran: random
RLU: relative light unit
ROS: reactive oxygen species
r.t.: room temperature
SA: *Staphylococcus aureus*
SAR: structure–activity relationship
SOSG: singlet oxygen sensor green
Surf‐EH3A: acrylate of surfactant ECOSURF EH‐3
TTC: 2,3,5‐triphenyltetrazolium chloride
UV: ultraviolet

## 1 Introduction

Antimicrobial resistance (AMR) is one of the most pressing global health challenges of the century, associated with an estimated 1.27 million deaths directly attributable to bacterial AMR in 2019 and projected to increase further if current trends continue [1]. Its burden extends beyond mortality to substantial healthcare costs and to the persistence of hospital‐acquired infections caused by ESKAPE pathogens [2, 3, 4]. Antibiotic misuse, incomplete treatment regimens, and the persistence of biofilm‐associated infections in chronic and healthcare‐associated settings all contribute to this trajectory, and demand therapeutic strategies that circumvent, rather than exacerbate, the selection of resistant strains. Antimicrobial photodynamic therapy (aPDT) has emerged as one legit such strategy: it relies on the light‐activated generation of reactive oxygen species (ROS) by a photosensitiser (PS) [5, 6] which inflict oxidative damage on bacterial membranes, proteins, and nucleic acids through both type I and type II pathways [7, 8]. Because this damage is multi‐target and largely non‐selective, resistance is difficult to develop [9, 10, 11]. aPDT is moreover intrinsically dose‐ and localisation‐controlled, combining efficacy against multidrug‐resistant strains with a low propensity for driving resistance selection [11, 12].

An effective aPDT PS must combine several properties: strong absorption at a therapeutically usable wavelength, efficient generation of ROS upon irradiation, adequate photostability, and biocompatibility towards host tissues [12]. Among the classes explored, ruthenium(II) polypyridyl complexes have attracted sustained interest owing to their intense metal‐to‐ligand charge‐transfer (MLCT) absorption in the visible region, high ^1^O_2_ quantum yields under blue‐light irradiation, chemical stability, and photophysics that can be tuned through ligand modification [10, 13, 14, 15, 16]. Their activity against clinical bacterial isolates, including antibiotic‐resistant strains, frequently exceeds that of organic PSs [9, 10, 13]. Their use in vivo, however, is limited by two persistent obstacles: poor aqueous solubility, requiring co‐solvents such as DMSO that themselves constrain the tolerated dose range, and inefficient penetration of complex environments such as biofilms and respiratory mucus, which shield bacterial targets from small‐molecule PSs [17]. Overcoming these limitations without sacrificing the intrinsic photophysical performance of the Ru core is a prerequisite for translating aPDT towards clinically relevant scenarios such as chronic pulmonary infections and infected wounds.

Incorporating PSs into supramolecular or macromolecular carriers is a natural response to these constraints, and a range of nanoplatforms has been developed to that end [18]. Lipid‐based systems, inorganic nanoparticles, and stimuli‐responsive hybrids have each been shown to improve PS delivery and antimicrobial performance [19, 20, 21, 22, 23, 24, 25, 26, 27, 28, 29], confirming that scaffold engineering is a productive route to overcoming the intrinsic limitations of small‐molecule PSs. Within this landscape, polymeric carriers offer distinctive advantages: methylene‐blue‐loaded PLGA nanoparticles and chlorin e6–triphenylphosphonium/PEI micelles, for example, enhance PS solubility, prevent aggregation, and enable controlled release [30, 31].

Covalent polymer–PS conjugates stand out within this landscape as a particularly attractive, yet less explored, route. Unlike encapsulation‐based systems, in which the PS can leak or redistribute during storage or use, covalent grafting to a polymer backbone anchors the photoactive centre to a scaffold whose composition, charge, and amphiphilicity can be tuned independently of the PS itself. The polymer confers water solubility on otherwise hydrophobic PSs, modulates their interaction with bacterial surfaces, and enables delivery in aqueous formulations free of the vehicle‐imposed dose limits of small‐molecule administration [17, 18]. Despite these advantages, the coupling of ruthenium complexes to tailored polymer backbones remains comparatively little explored, and the synthetic strategies available to achieve it have rarely been compared directly on a shared compositional space. Two strategies dominate: direct copolymerisation of PS‐bearing monomers, most commonly by free‐radical copolymerisation (FRP), and post‐polymerisation modification (PPM) of reactive precursor polymers, in which the PS is grafted onto an established backbone. Each has recognised advantages — FRP for its operational simplicity and its access to a broad compositional space, PPM for its modularity and its separation of polymerisation and functionalisation steps — but the two are typically deployed in isolation, so that the influence of synthetic strategy on the resulting material is difficult to assess.

In this work (Scheme 1), the challenges associated with delivering ruthenium‐based PSs to the site of action are addressed by integrating them into modular, low‐fouling acrylic polymer backbones. Two complementary synthetic strategies are deployed in parallel within a single design framework: (i) free‐radical copolymerisation (FRP) of Ru‐bearing acrylic monomers with hydrophilic and surfactant comonomers, and (ii) post‐polymerisation modification (PPM) of a reactive pentafluorophenyl‐acrylate precursor with amine‐functionalised Ru complexes. This dual approach allows the same compositional space to be accessed by two orthogonal routes, and the influence of synthetic strategy on the resulting material to be evaluated directly.

**SCHEME 1:**
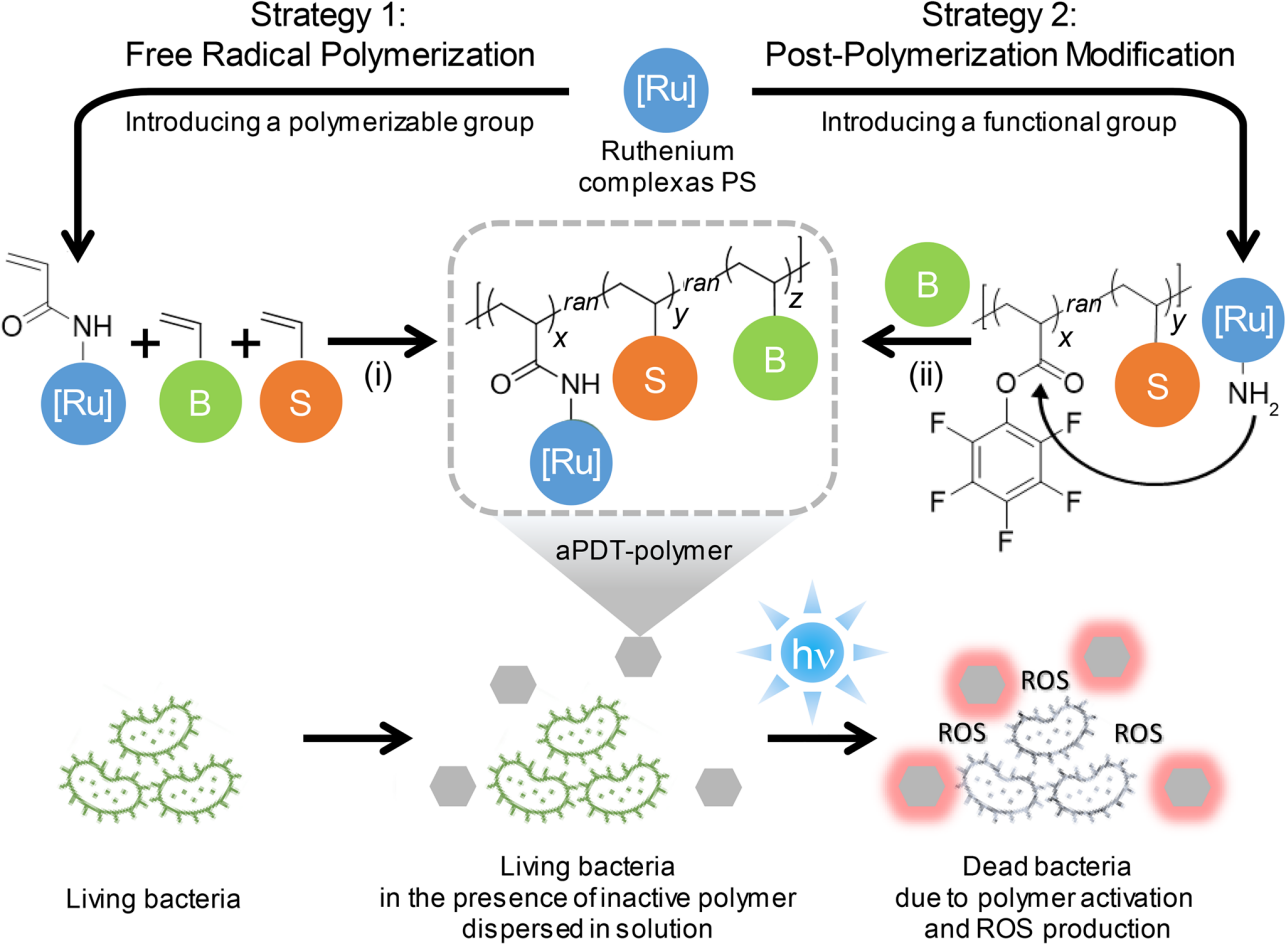
Schematic overview of the study strategy. Two adjustable complementary synthetic routes, free‑radical polymerisation (FRP) and post‑polymerisation modification (PPM), are used to generate tailored polymers bearing ruthenium complexes ([Ru], as photosensitizer (PS)) together with optional functional moieties, including biocompatible grafts (B) and surfactants (S). In strategy 1 (i), the polymerisation is performed using an initiator, whereas in strategy 2 (ii), an amine on the Ru complex reacts with a pre‐made active ester co‐surfactant polymer, and an excess of B is used to substitute the remaining esters. All resulting polymers disperse readily in aqueous media. Systematic variation of the polymer composition (x, y, z repeat units; *ran* standing for random) yields a library of materials that are subsequently evaluated in antimicrobial photodynamic therapy (aPDT) assays against Gram‑positive and Gram‑negative bacteria from three clinically relevant species. The resulting dataset enables the delineation of structure‐activity relationships across the polymer series.

The platform rationally integrates four functional features within a single macromolecular architecture: (i) a hydrophilic biocompatible backbone (*N*‐hydroxyethyl acrylamide, acrylic acid) ensuring solubility in aqueous and biological media; (ii) hydrophobic surfactant side chains expected to engage the bacterial cell membrane; (iii) charged groups cationic Ru centres, which mediate direct electrostatic association with negatively charged bacterial surfaces, and anionic carboxylates, which modulate amphiphilic balance, confer pH‐responsiveness, and may support divalent‐cation‐mediated bridging with the bacterial envelope; and (iv) covalently grafted ruthenium complexes that generate ROS under blue‐light irradiation, providing spatio‐temporal control of antibacterial action. The designed combination of these features within one polymer backbone is expected to provide a “multidentate effect,” i.e., supporting cooperative, multivalent engagement with bacterial surfaces, conferring activity not only on planktonic cells but also on biofilms. The resulting library of seven aPDT‐polymers is benchmarked against a reference ruthenium complex and evaluated across three clinically relevant bacterial species, with the dual aim of establishing structure–activity relationships that guide rational polymer design and demonstrating that the platform is intrinsically extensible (beyond the specific Ru PSs reported here) to other transition‐metal complexes and polymer–metal architectures, with applications well beyond aPDT.

## 2 Results and Discussion

### 2.1 Synthesis and Characterisation of the Polymer Library

#### 2.1.1 Overview of the Dual‐Strategy Design

Two complementary synthetic strategies were deployed in parallel to generate the polymer library (Scheme 2 and Table 1): free‐radical copolymerisation (FRP) of Ru‐bearing acrylic monomers with hydrophilic and surfactant comonomers, and post‐polymerisation modification (PPM) of a reactive pentafluorophenyl‐acrylate copolymer with amine‐functionalised Ru complexes. This dual approach enables the same compositional space to be accessed by two orthogonal routes and provides direct comparison of how synthetic strategy influences the resulting materials.

**SCHEME 2:**
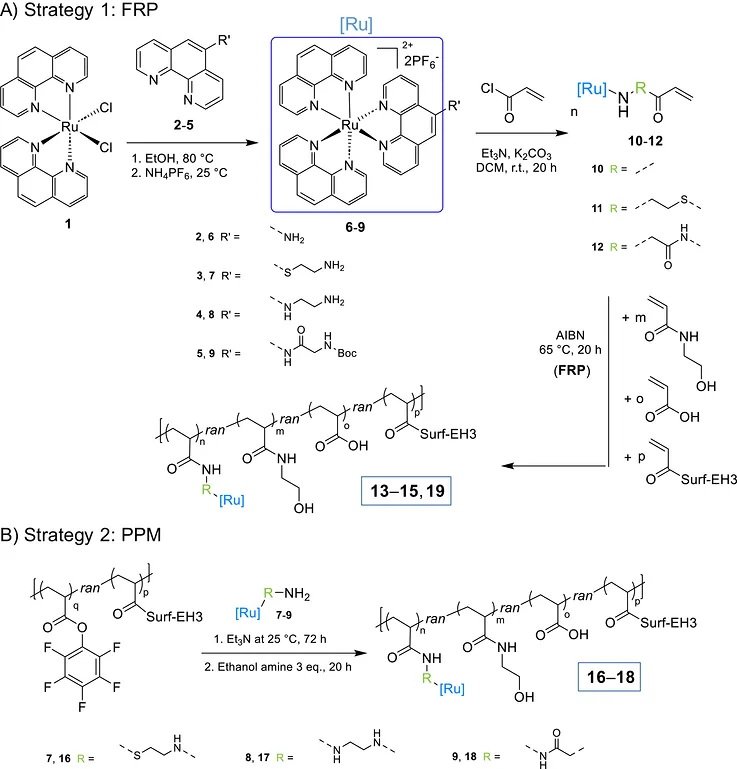
Two orthogonal synthetic routes to ruthenium‐functionalised polymers, deployed in parallel within a single design framework. Refer to Table 1 for the specific composition of final polymers (**13**–**19**, differing in their R linker). *ran* stands for random.

**TABLE 1:** Detailed composition of the polymers obtained through either of the two synthesis strategies, i.e., copolymerisation (FRP) or post‐polymerisation modification (PPM).

aPDT‐polymers (Strategy, charge, and composition)	Strategy	*m* HEAAm Mol%	*n* [Ru] Mol%	*o* AA Mol%	*p* Surf. Mol%	R linker
**13** (FRP · [+] · 0% AA · 0.5% Ru)	FRP	99.5	0.5	0	0.0	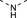
**14** (FRP · [+] · 0% AA · 0.6% Ru)	FRP	95.0	0.6	0	4.4	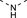
**15** (FRP · [+] · 0% AA · 1.0% Ru)	FRP	97.0	1.0	0	2.0	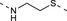
**16** (PPM · [‐] · 20% AA · 0.5% Ru)	PPM	77.5	0.5	20	2.0	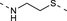
**17** (PPM · [‐] · 20% AA · 1.3% Ru)	PPM	76.7	1.3	20	2.0	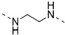
**18** (PPM · [‐] · 71% AA · 2.0% Ru)	PPM	25.0	2.0	71	2.0	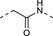
**19** (FRP · [‐] · 20% AA · 1.0% Ru)	FRP	77.0	1.0	20	2.0	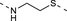

AA, acrylic acid; HEAAm, *N*‐hydroxyethyl acrylamide. Data for build‐in ratios (*m*, *n*, *o*, *p*) refer to mole fractions of the corresponding monomers, calculated from NMR measurements. Positively‐ or negatively‐charged moieties correspond to the R group and AA, respectively. * **17** carries trithiocarbonyl end groups. Composition labels used elsewhere in the manuscript combine the polymer number, acrylic‐acid content, and ruthenium loading, with charge sign ([+], cationic; [‐], anionic) added where relevant to interpretation. *Surf*. stands for surfactant.

In free‐radical copolymerisation (FRP) route (Scheme 2A), [Ru(phen)_2_Cl_2_] (**1**) is functionalised with an amine‐bearing phenanthroline ligand (**2**–**5**) to yield an amine‐modified Ru complex (**6**–**9**) with different linker structures R’; acrylation with acryloyl chloride affords the polymerisable Ru monomers (**10**–**12**); free‐radical copolymerisation with *N*‐hydroxyethyl acrylamide (HEAAm) and the surfactant comonomer Surf‐EH3A in the presence of AIBN yields polymers **13**–**15** and **19**. In post‐polymerisation modification (PPM) route (Scheme 2B), pentafluorophenyl acrylate (PFPA) is first copolymerised with Surf‐EH3A to give the reactive precursor polyPFPA‐*ran*‐Surf‐EH3; the same amine‐modified Ru complexes (**7**, **8**, **9**) are then coupled to this backbone by active ester aminolysis in the presence of triethylamine, and the remaining PFP esters are consumed by treatment with ethanolamine, yielding polymers **16**–**18**. In both routes, the final polymers share the same generic architecture, and the same amine‐modified Ru precursors (**7**–**9**) can be used in either strategy, providing direct access to a common compositional space via two orthogonal chemistries. Detailed procedures and characterisation data are provided in the Supporting Information (Schemes S1–S15, NMR spectra S1–S19, GPC data S1–S13).

#### 2.1.2 FRP Route

The FRP strategy proceeded through two synthetic stages. First, a family of amine‐functionalised Ru(II) phenanthroline complexes was prepared: the precursor *cis*‐[Ru(phen)_2_Cl_2_] (**1**) was reacted with functional phenanthroline ligands (**2**–**5**), either commercially available (**2**) or prepared through modified literature procedures (**3**–**5**), to afford complexes **6**–**9** in 50%–70% yield. In the custom‐modified ligands **3**–**5**, an alkyl spacer was introduced to enhance the reactivity of the primary amine group in subsequent coupling steps while preserving the photophysical characteristics of the Ru core. Notable procedural improvements included an optimised purification protocol for ligand **3** (yield increased from ∼60% to over 90%), microwave‐assisted synthesis of ligand **4** (reaction time reduced from overnight to 3 h), and a refined purification of ligand **5** (yield ∼90%). Full procedures are provided in the Supporting Information.

Polymerisable double bonds were then introduced by acylation of complexes **6**, **7**, and **9** with acryloyl chloride, affording the Ru‐based aPDT monomers **10**–**12** in yields above 75%. These were subsequently copolymerised with *N*‐hydroxyethyl acrylamide (HEAAm) and, where relevant, with the surfactant comonomer ECOSURF EH‐3 acrylate (Surf‐EH3A), yielding polymers **13**–**15** (Scheme 2A). HEAAm was selected as the major component (typically over 90 mol% in the feed) to provide a non‐fouling, biocompatible backbone and to ensure sufficient hydrophilicity for aqueous solubility of the aPDT unit. The surfactant comonomer was used to modulate the hydrophilic–hydrophobic balance and overall amphiphilicity of the resulting polymers.

Batch‐averaged compositions were determined by ^1^H NMR spectroscopy and revealed that built‐in ratios systematically differed from monomer feed ratios, as expected for FRP of comonomers with distinct reactivity ratios and polarities, an intrinsic feature of FRP that will be discussed further below (§2.1.6). Distinctively, Polymer **19**, containing pendent carboxylate groups from copolymerisation with acrylic acid alongside HEAAm, Surf‐EH3A, and Ru‐complex monomer **11**, was added to the library as an anionic FRP counterpart to the PPM‐derived polymer **16**, allowing a direct comparison of the influence of net charge at otherwise similar composition.

#### 2.1.3 PPM Route

The PPM strategy started from a well‐defined reactive precursor: the copolymer polyPFPA‐*ran*‐Surf‐EH3, prepared by FRP of pentafluorophenyl acrylate (PFPA) with the Surf‐EH3A comonomer at a 98:2 molar ratio (Scheme S7). Successful synthesis and characterisation of this reactive backbone by ^1^H NMR and GPC (*M*_n_ = 20 kDa, *Đ* = 1.5) provided a scaffold for the subsequent grafting of Ru moieties as PS.

Post‐polymerisation grafting was then evaluated with the amine‐functionalised complexes **7**–**9**. Preliminary attempts with complex **6**, in which the amine group is directly attached to the aromatic core of the ligand, did not yield the expected product; this is consistent with reduced reactivity of aromatic amines towards active esters. The introduction of an alkyl spacer in complexes **7**–**9** overcame this limitation, and two PPM approaches were then investigated: (path 1) partial modification of polyPFPA‐co‐Surf‐EH3 with the aPDT complexes, followed by aminolysis of the remaining PFP esters with excess ethanolamine to yield HEAAm‐type repeat units; and (path 2) reaction of polyPFPA‐*ran*‐Surf‐EH3 with amine‐functionalised phenanthroline ligands, followed by attempted complexation with *cis*‐[Ru(phen)_2_Cl_2_] **1**. While path 2 yielded macro‐ligands, subsequent complexation was unsuccessful (Scheme S15). Consequently, the polymers reported here were prepared through path 1, followed by dialysis against water for 24–72 h until the wash turned colourless, ensuring removal of unreacted low‐molar mass components. Path 1 afforded polymers **16**–**18** in isolated yield (Scheme 2B).

Comprehensive characterisation of the PPM‐derived polymers was performed to confirm the outcome of the PPM reaction. ^1^H NMR spectra of the modified polymers exhibited characteristic aromatic protons in the 7–8 ppm range attributable to the ruthenium complex, together with ethyl protons of the incorporated ethanolamine in the 2.7–4 ppm region. Complementary evidence for covalent attachment (from ^1^ ^9^F NMR, UV‐visible spectroscopy, SEC‐UV chromatography, and electrophoretic behaviour) is discussed below (§2.1.5). Together, the FRP and PPM routes provided a library of seven aPDT‐polymers spanning a broad compositional space, at gram scale and with high purity.

#### 2.1.4 Batch‐Averaged Compositions

The resulting library of seven aPDT‐polymers (**13**–**19**), together with the reference Ru complex **I** ([Ru(phen)_3_]^2^^+^·2PF_6_^−^), are presented in Table 1. Batch‐averaged compositions, determined by ^1^H NMR spectroscopy, span a broad range of the compositional space accessible to this aPDT platform: HEAAm content between 25 and 99.5 mol%, ruthenium content between 0.5 and 2 mol%, surfactant content between 0 and 4.4 mol%, and acrylic acid content between 0 and 71 mol%. Four distinct R groups (Table 1) are represented across the library, corresponding to the four amine‐functionalised Ru complexes used as monomers or PPM reagents. This diversity spans, within a single library, both synthetic strategies (four polymers via FRP, three via PPM), both charge signs (cationic **13**–**15**, through the Ru complex; anionic **16**–**19**, through pendent carboxylates), and a broad range of the amphiphilic balance. Notably, and as anticipated for FRP, the built‐in ratios in polymers **13**–**15** and **19** differed from the feed ratios in the polymerisation reaction, an important point that we return to in §2.1.6 in the context of intra‐population compositional heterogeneity. In what follows, this compositional diversity is exploited to establish structure–activity relationships across the library.

#### 2.1.5 Covalent Attachment: Converging Evidence

The covalent character of the Ru–polymer association is supported by the convergence of four independent lines of evidence, as follows:

##### 2.1.5.1 Size‐Exclusion Chromatography With Simultaneous UV and RI Detection at a Ru‐Sensitive Wavelength

SEC coupled to RI and UV detection at 280 nm, corresponding to a strong phenanthroline absorption of the Ru(II) complex, was performed on the aPDT‐polymers alongside the reference complex **7** (example shown for polymer **16** in Figure 1A; raw elugrams of all polymers and Ru complex are provided in the Supporting Information). The 280 nm signal of the elutes follows the refractive‐index (RI) signal across the entire polymer molar mass distribution, indicating that the ruthenium chromophore is present across the polymer population rather than segregated in a specific fraction. Crucially, no significant low‐molar mass peak corresponding to a free ruthenium complex is observed at the elution volume of **7**, confirming that the purification protocol effectively removes non‐covalently associated complex. Moderate asymmetry between the RI and UV traces is usually observed in the SEC instrument due to a slight detector offset.

**FIGURE 1:**
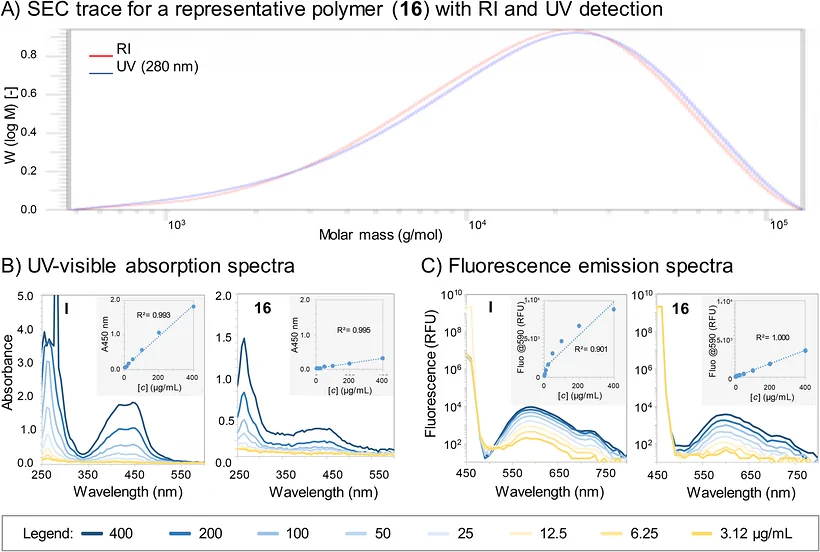
Structural and photophysical characterisation of the aPDT‐polymers, benchmarked against the reference complex I ([Ru(phen)_3_]^2^^+^·2PF_6_^−^). (**Panel A**) Size‐exclusion chromatogram of polymer **16** (representative example), recorded in parallel by refractive‐index (RI, red trace) and UV detection at 280 nm (blue trace). (**Panel B**) UV–visible absorption spectra of **I** and polymer **16** in water. Inset: absorbance at 450 nm as a function of concentration. (**Panel C**) Fluorescence emission spectra of the same samples upon excitation at 450 nm. Inset: emission intensity as a function of concentration. Refer to the Supporting Information for experimental details and characterisation of the full polymer library. Data represent means ± SD of *n* = 3, representative of three independent experiments.

##### 2.1.5.2 ^1^ ^9^F NMR of the PPM Step

^1^ ^9^F NMR spectra of the PPM‐derived polymers show near‐complete disappearance of the characteristic pentafluorophenyl resonances upon aminolysis, indicating that essentially all reactive ester sites of the polyPFPA‐*ran*‐Surf‐EH3 precursor have been consumed (Scheme S13, and NMR spectra S19).

##### 2.1.5.3 ^1^H NMR Resonance Shifts

Following coupling, the ^1^H NMR resonances of the linker units adjacent to the amine groups of the Ru complexes are shifted relative to the free amine groups of the pristine complex, consistent with amide bond formation to the polymer backbone rather than free amine (Schemes S1–S15, NMR spectra S1–S18). If unreacted complex monomer remained in the polymer samples, the NMR traces would show the corresponding double bond signal, but they are absent to the detection limit in all measured samples.

##### 2.1.5.4 Gel‐Electrophoretic Behavior

On agarose gel electrophoresis (Figure 2), the bare complex **I** migrates as a well‐defined cationic band, while the aPDT‐polymers migrate as their own distinct bands consistent with the net charge of each composition. For every polymer (**13**–**19**), no lane exhibits a separate free‐complex band at the migration position of **I**, providing an orthogonal confirmation that the ruthenium centres remain covalently associated with the polymer chains under the applied field.

**FIGURE 2:**
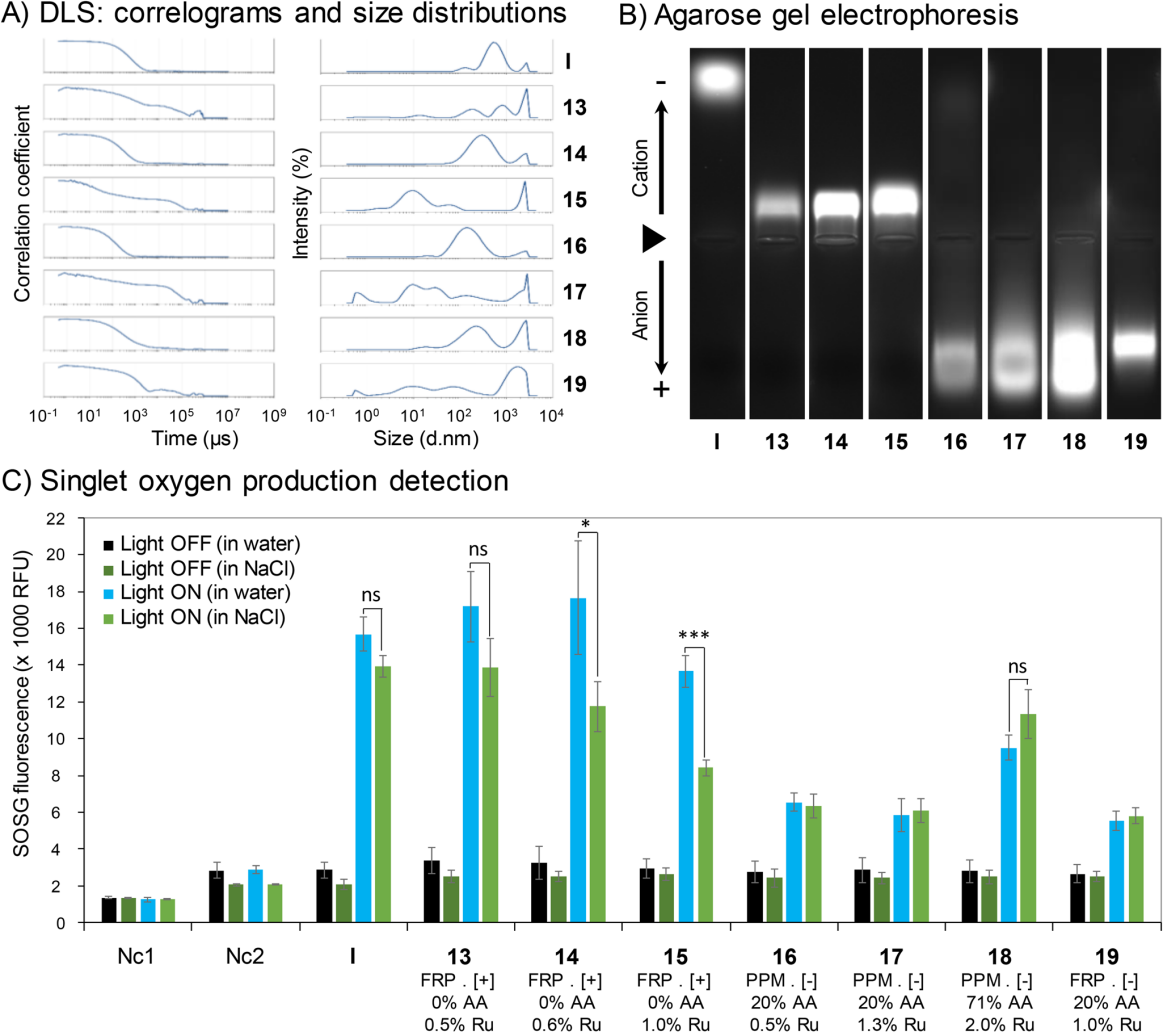
Physicochemical and photodynamic profiling of the aPDT‐polymer library. (**Panel A**) Intensity‐weighted hydrodynamic size distributions of the aPDT‐polymers and of **I** by dynamic light scattering (DLS) in Milli‐Q water at 1 mg/mL. (**Panel B**) Agarose gel electrophoresis of the aPDT‐polymers and of complex **I**, visualised by intrinsic Ru fluorescence. See Figure S6 for the Ru‐free polymer backbones. (Panel C) Singlet‐oxygen generation quantified by the SOSG fluorescence response at 50 µg/mL upon 450 nm irradiation. See the Supporting Information for experimental details. Data represent means ± SD of *n* = 3, from three independent experiments. Statistical significance: ^***^, *p* ≤ 0.001; ^**^, *p* ≤ 0.01; ^*^, *p* ≤ 0.05; ns, not significant.

Taken together, these four independent observations converge on the same conclusion: the ruthenium centres are covalently grafted onto the polymer backbones, rather than physically retained through nonspecific interactions.

#### 2.1.6 Compositional Heterogeneity and Acknowledged Limitation

A word is warranted here on the compositional resolution afforded by the present data. Free‐radical copolymerisation of monomers with different reactivity ratios and polarities is intrinsically expected to yield chain‐to‐chain variability, and the batch‐averaged compositions do not resolve the distribution of the different repeat unit types across individual polymer chains. Analogous, though distinct, considerations apply to PPM, where the local polarity of the increasingly modified backbone during active ester hydrolysis can affect the accessibility of the reactive sites. Complementary characterisation, including SEC‐UV analysis (§2.1.5), confirms that the ruthenium signal is distributed across the molar mass profile of the polymer ensembles rather than segregated in a specific fraction; however, chain‐level compositional resolution is not accessible with the methods used here. Accordingly, structure–activity claims made throughout this study should be understood as correlative associations between batch‐averaged compositional descriptors and antimicrobial performance across the library, rather than as causal attributions to specific molecular features.

### 2.2 Photophysical and Physicochemical Profiling

#### 2.2.1 Photophysics: Absorption and Emission

Absorption and fluorescence spectra of the aPDT‐polymers were recorded in water and in saline (0.9% NaCl) across a wide relevant concentration range. Polymer backbones lacking Ru moieties (**II** and **III**, used as controls) exhibited neither absorption nor fluorescence in the relevant spectral windows (Figure S3), confirming that both properties arise exclusively from the grafted Ru units. Figure 1 shows a representative comparison between the reference complex **I** and one aPDT‐polymer, **16**, chosen because it carries a high ruthenium loading and therefore provides a clear signature of Ru photophysics on a polymer backbone; the characterisation of the full library is presented in Figure S4.

Complex **I** displays the characteristic spectrum of a Ru(II) polypyridyl complex, with a strong phenanthroline absorption near 265 nm and a broader MLCT band between 425 and 455 nm peaking at ∼450 nm. Polymer **16** shows a comparable absorption pattern, though with intensities lower per unit mass than the free complex, consistent with the ∼50‐fold dilution of the Ru chromophore in the polymer matrix (as estimated from batch‐averaged compositions). Absorbance at 450 nm scales linearly with polymer concentration up to the highest tested (400 µg/mL), whereas linearity for **I** is preserved only up to 100–200 µg/mL due to detector saturation at this high complex concentration. Upon excitation at 450 nm, both **I** and **16** emit at ∼590 nm, and the polymer emission remains linear with concentration across the full range explored.

The full library (Figures S3 and S4) reveals two additional trends worth noting. First, ruthenium loading correlates with fluorescence intensity across most compositions. Second, however, polymers of matched Ru content (e.g., **13** and **16**) display distinct emission profiles, consistent with a substantial contribution of the local microenvironment (aggregation state, backbone polarity, and protonation of proximal carboxylates) to the emission properties of the Ru(II) centres (see also §2.2.2).

#### 2.2.2 Colloidal Behavior in Aqueous Media

Because the aPDT‐polymers are designed to combine hydrophilic backbones with hydrophobic surfactant side chains and, in most cases, mutually oppositely charged groups within a single macromolecular architecture, their state in aqueous solution is unlikely to be that of individually solubilised chains. Dynamic light scattering (DLS) was therefore used to characterise the colloidal state of the polymer library in Milli‐Q water at 1 mg/mL (Figure 2A).

The bare complex **I**, used as a reference, displays a clean monomodal correlation function decaying between ∼200 and 3000 µs, consistent with the small‐molecule nature of the free complex. In contrast, all aPDT‐polymers show correlograms indicative of aggregated species, but with two distinguishable behaviours. Polymers **14**, **16**, and **18** yield relatively clean monomodal decays, consistent with well‐defined nano‐aggregate populations of moderate polydispersity. Polymers **13**, **15**, **17**, and **19**, exhibit multi‐modal correlograms with extended tails towards long decay times (10^4^–10^6^ µs), consistent with the coexistence of nano‐aggregates and larger structures, and with broader size distributions overall. It is noteworthy that this observation is independent of the synthetic route, as **13**–**15** and **19** were obtained via FRP while **16**–**18** were obtained via PPM.

This distinction is consistent with the compositional profile of each polymer. The “clean” nano‐aggregate polymers (**14**, **16**, and **18**) carry either a single dominant charge sign or a moderate content of charged groups. The multi‐modal polymers (**13**, **15**, **17**, and **19**) either carry high densities of a single charge or bear both cationic (Ru) and anionic (acrylic acid) groups within the same chain, favouring intra‐ and intermolecular polyion‐complex (PIC) formation, further discussed in §2.2.4. In all cases, the polymers therefore form colloidal dispersions rather than being molecularly dissolved, and the intrinsic colloidal state is one of the physicochemical descriptors that co‐varies with antimicrobial activity across the library (§2.4).

#### 2.2.3 Passive Diffusion Assays

The mobility of the aPDT‐polymers in media of biological relevance was assessed by monitoring their fluorescence‐tracked diffusion in water and in artificial sputum medium (ASM), a mucin‐ and DNA‐rich model of respiratory secretions. As shown in Figure S5, the polymers reach diffusion equilibrium within experimentally relevant time frames across all tested media; diffusion is slower in ASM than in water, consistent with reduced mobility in the viscous, macromolecule‐rich environment.

This assay is best understood as a descriptive, integrative readout rather than as a mechanistic probe of specific polymer–medium interactions: viscosity, osmotic effects, and aggregation‐state changes all contribute to the observed mobility, alongside any specific polymer–ASM interactions. Therefore, the results reported here describe the net mobility of the polymers under standardised conditions, and enable a relative comparison across the library, without deconvoluting the individual physical contributions. Complementary characterisation of the colloidal state of the polymers in aqueous media, including DLS size distributions (§2.2.2), further supports the interpretation that the polymers retain their nano‐aggregate architecture across the media tested. Together, these observations indicate that the polymer platform is compatible with the physicochemical constraints of respiratory secretions, a prerequisite for potential applications in that context.

#### 2.2.4 Electrophoretic Behavior and Polyion‐Complex Formation

The intrinsic fluorescence of the ruthenium centres allows agarose‐gel electrophoresis to probe both the net charge and the mobility of the aPDT‐polymers under an applied field (Figure 2B). Polymer backbones lacking Ru moieties are undetectable on the gel (Figure S6), so all bands observed here originate from Ru‐bearing species. Under the conditions used, the bare complex **I** migrates towards the cathode as a well‐defined cationic band, as expected for its 2+ charge.

The polymer library separates into two groups. Polymers **13**, **14**, and **15**, all synthesised by FRP with only cationic and neutral repeat units, migrate as single bands towards the cathode, consistent with net cationic charge. Distinctively, polymers **16**, **17**, **18**, and **19**, all bearing pendant carboxylate groups, migrate primarily towards the anode, but with a more complex pattern: **16**, **17**, and **18** show a doublet of anion‐migrating bands (two closely spaced anionic populations), whereas polymer **19** shows a single, more compact anionic band. Notably, no cation‐migrating band is observed in any of these four lanes, indicating the absence of any free‐complex fraction and confirming the anionic net charge of the whole polymer population in each case. The presence of two anionic sub‐populations for **16**, **17**, and **18** is consistent with a degree of chain‐to‐chain compositional or conformational heterogeneity within these PPM‐derived batches, and possibly with the coexistence of free chains and intra‐ or intermolecular polyion‐complex (PIC) assemblies between the cationic ruthenium centres and the anionic carboxylates. Polymer **19**, obtained through FRP rather than PPM, shows a more homogeneous single‐band profile under the same conditions, which may reflect a difference in intra‐batch architectural distribution between the two synthetic strategies (see §2.1.6). Importantly, no distinct free‐complex band is observed in any polymer lane, providing an additional line of evidence that the ruthenium moieties are covalently associated with the polymer backbones rather than physically retained. Grouping by mobility can be interpreted as indicating similar behaviour under the conditions used; a definitive assessment of size and conformation would require charge‐matched conditions and complementary characterisation (e.g., SAXS or viscometry). Complementary electrophoresis experiments in artificial sputum medium (ASM), which includes DNA as a component, revealed that cationic polymers **13**, **14**, and **15** additionally sequester DNA through electrostatic interaction, forming polyplex‐like assemblies visible as retarded bands (Figure S7). This behaviour is consistent with the interaction of these polymers with negatively charged bacterial surfaces described in §2.3.1, and may also be relevant to interaction with genomic DNA released from bacteria under aPDT.

#### 2.2.5 ROS Generation

The photodynamic action of the aPDT‐polymers ultimately relies on their ability to generate reactive oxygen species (ROS) upon blue‐light irradiation. Singlet oxygen (^1^O_2_) was chosen as the primary readout, quantified through the fluorescence response of the SOSG dye upon irradiation at 450 nm (Figure 2C; kinetic data in Figure S10). Pre‐illumination addition of sodium azide reduced or entirely suppressed SOSG fluorescence for both **I** and representative polymers, confirming that the signal reports on ^1^O_2_ specifically (data not shown). Under prolonged blue‐light irradiation, the aPDT‐polymers retain their electrophoretic and fluorescence signatures (Figure S8), confirming their photostability across the timescales used in the antibacterial assays and ruling out photodegradation as a confounding factor for the ROS‐generation measurements described below.

At matched compound concentration (50 µg/mL), all aPDT‐polymers generated detectable ^1^O_2_ under blue‐light irradiation, in some cases outperforming the free complex **I** on a per‐mass basis (Figure S9). Given that the polymers dilute the Ru chromophore approximately fifty‐fold relative to **I** (§2.2.1), this indicates that the polymer environment is at least as efficient as, and can enhance, the intrinsic ^1^O_2_ production of the Ru(II) centres. Two features complicate this interpretation, however, and warrant explicit acknowledgement. First, the SOSG response is itself sensitive to the local environment and to electrostatic effects; thus, it is noteworthy that negatively charged polymers (**16**, **17**, **18**, **19**) may partially repel the anionic SOSG probe, leading to underestimated per‐Ru ^1^O_2_ generation. Second, aggregation state (§2.2.2) modulates the excited‐state photophysics of the Ru centres: accordingly, polymers forming denser or larger aggregates may show self‐quenching or reduced accessibility of the chromophore to dissolved oxygen. Consistent with this interpretation, cyclic irradiation reveals distinct kinetic profiles across the library (Figure S10): polymers **16** and **19**, for example, exhibit slower but sustained ^1^O_2_ generation, contrasting with the rapid but plateauing response of **I**. These distinct kinetic signatures indicate that the environmental factors identified above translate into functionally different photodynamic behavior, but not merely into differences in absolute yield. A direct determination of the singlet‐oxygen quantum yield for each polymer (requiring either comparative photolysis against a reference PS or direct detection of ^1^O_2_ phosphorescence at 1270 nm) would refine the per‐chromophore ranking of ^1^O_2_ production across the library and warrants dedicated follow‐up work.

Crucially, ^1^O_2_ output does not monotonically follow Ru loading: polymer **18**, the most Ru‐rich of the library, is not the most efficient producer per unit chromophore, whereas polymers **13** and **14** consistently emerge as among the most efficient. This decoupling of ^1^O_2_ output from Ru content is one of the compositional signatures the multivariate analysis (§2.4) exploits to identify structure–activity trends.

### 2.3 Antibacterial Activity and Biocompatibility

#### 2.3.1 Bacterial Interaction Assay

The intrinsic fluorescence of the ruthenium moieties was also exploited to quantify the interaction of the polymers with bacteria, both as planktonic cells and in biofilm state. Following incubation with the aPDT‐polymers, samples were centrifuged, and the residual fluorescence in the supernatant provided an indirect measure of the polymer–bacterial association. The results obtained with *P. aeruginosa* PA19 are shown in Figure 3A and were consistent across the other bacterial strains tested (Figure S11).

**FIGURE 3:**
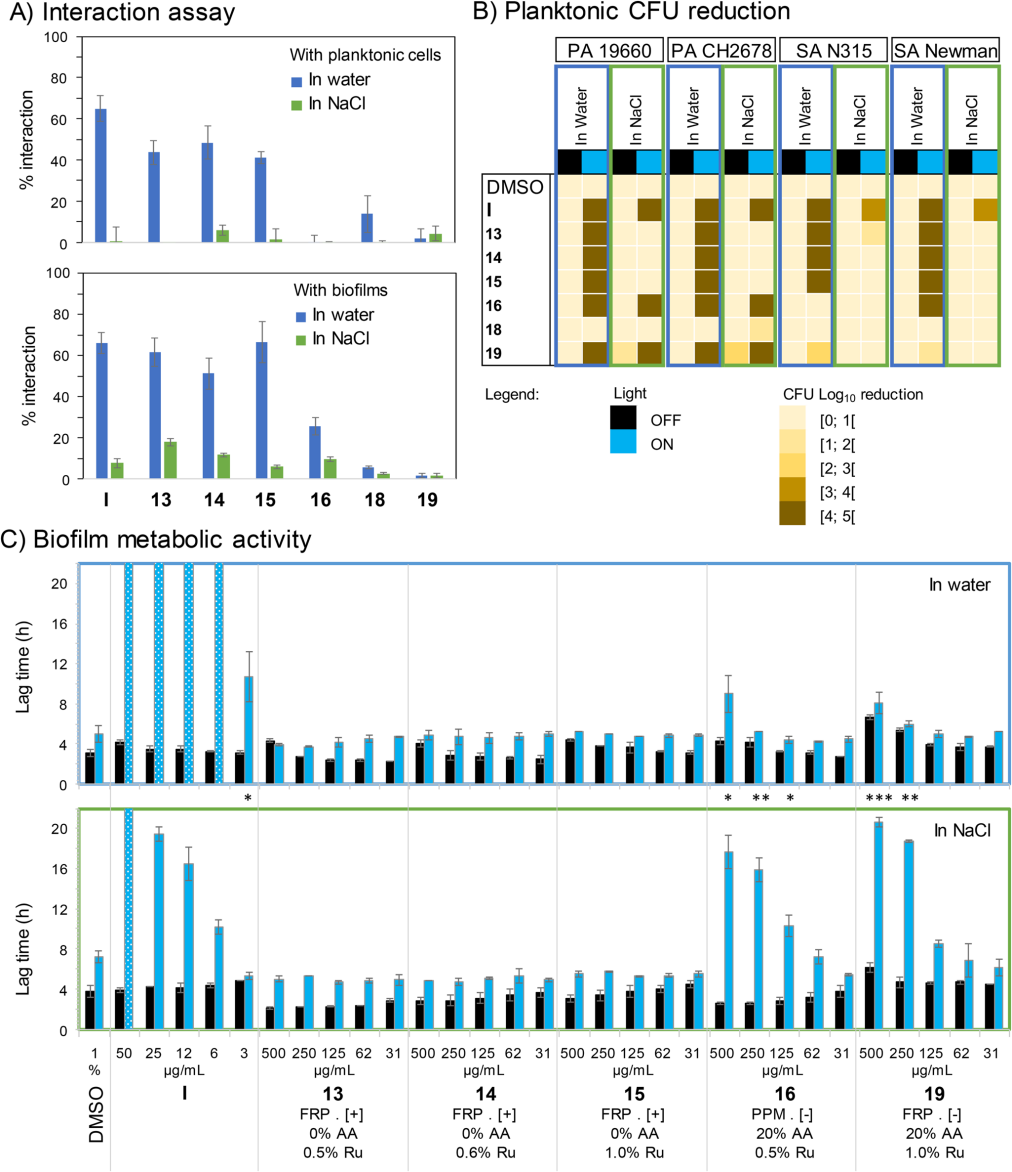
Antibacterial performance of the aPDT‐polymer library and of complex **I** under blue‐light irradiation. (**Panel A**) Interaction of the aPDT‐polymers with *P. aeruginosa* PA19, as planktonic cells (left) and pre‐formed biofilms (right), in water and in 0.9% NaCl. Equivalent data for the other strains are provided in Figure S11. (**Panel B**) Colour‐coded bacterial‐load reduction (log_10_ CFU/mL) following treatment with **I** or the aPDT‐polymers, in the dark (OFF) or under blue‐light irradiation (ON), against *P. aeruginosa* (PA19, CH2678) and *S. aureus* (N315, Newman), in water and in 0.9% NaCl. Corresponding MIC values are reported in Table 2. See Figure S13 for complementary results including *E. coli*. (**Panel C**) Antibiofilm activity against *P. aeruginosa* PA19 by TTC‐based kinetic assay; longer lag times indicate greater delay of biofilm recovery. Dotted bars indicate lag times >22 h. Complementary data for *P. aeruginosa* CH2678 in Figure S15. See the Supporting Information for experimental details. Data represent means ± SD of *n* ≥ 3, from three independent experiments. Statistical significance: ^***^, *p* ≤ 0.001; ^**^, *p* ≤ 0.01; ^*^, *p* ≤ 0.05; ns, not significant.

In water, cationic species (complex **I** and polymers **13**, **14**, and **15**) showed clear association with bacteria, consistent with electrostatic engagement of the cationic Ru centres with the net negatively charged bacterial surface. Anionic polymers **16**, **18**, and **19** showed only minimal or no association under the same conditions, as expected from the electrostatics. In 0.9% NaCl, however, all such interactions were largely abolished for both cationic and anionic species, indicating that they are dominated by electrostatic mechanisms rather than by specific molecular recognition. Analogous trends were observed for association with pre‐formed biofilms (Figure 3A, biofilm panel), with polymer **16** as a notable exception, retaining partial interaction under saline conditions.

#### 2.3.2 MIC and CFU Reduction

Minimum inhibitory concentrations (MIC) were determined for the polymer library and for complex **I** against three clinically relevant bacterial species – *P. aeruginosa* (PA19 and CH2678), *S. aureus* (N315 and Newman), and *E. coli* (DH5ɑ) – under blue‐light irradiation (Table 2), together with bacterial‐load reductions quantified by CFU counts (Figure 3B). The reference complex **I** could only be evaluated up to concentrations delivering 1% v/v DMSO in the assay well, above which vehicle‐induced toxicity would confound the readout; the polymers, being water‐dispersible, do not require DMSO and could be tested throughout their full active range. This is itself a practical advantage of the polymer platform for aqueous antimicrobial applications.

**TABLE 2:** MIC (in µg/mL) of **I** and aPDT‐polymers: Determination using three bacterial species.

	PA19		SA N315		*E. coli* DH5ɑ	
	OFF	ON	OFF	ON	OFF	ON
DMSO	>1%	>1%	>1%	>1%	>1%	>1%
**I**	>50	0.8	>50	3.1	>50	3.1
**13** (FRP · [+] · 0% AA · 0.5% Ru)	>500	15.6	>500	31.3	>500	15.6
**14** (FRP · [+] · 0% AA · 0.6% Ru)	>500	7.8	>500	15.6	>500	3.9
**15** (FRP · [+] · 0% AA · 1.0% Ru)	>500	15.6	>500	31.3	>500	15.6
**16** (PPM · [‐] · 20% AA · 0.5% Ru)	>500	0.5*	>500	>500	>500	>500
**18** (PPM · [‐] · 71% AA · 2.0% Ru)	>500	>500	>500	>500	>500	>500
**19** (FRP · [‐] · 20% AA · 1.0% Ru)	>500	62.5	>500	>500	>500	>500

Measurements were done when mixing bacteria and reagents under dark conditions (OFF), or after exposure to light for 30 min (ON). Reagents were tested for concentrations ranging from 0.05 to 50 µg/mL (Ru), or from 0.5 to 500 µg/mL (aPDT‐polymers). The bacteria tested were *P. aeruginosa* (PA19), *S. aureus* (N315) and *E. coli* (EC). **17** was not tested here owing to insufficient material at the time of the assay. *, unexpected behavior (read below). *‘*>1%' indicates that the MIC of reference complex I could not be determined within the tolerated vehicle limit of 1% v/v DMSO, above which DMSO alone becomes cytotoxic in the assay. The polymers are water‐dispersible and were tested without DMSO.

Across the library, MIC values and CFU reductions revealed clear compositional trends. Cationic polymers **13**, **14**, and **15** were the most active against planktonic *S. aureus* N315 in water, consistent with their strong cationic association with the negatively charged bacterial envelope. Anionic polymers **16** and **19** were most active against MDR *P. aeruginosa* under saline conditions, an environment that resembles the ionic strength of physiological fluids and abolishes purely electrostatic interactions. Under illumination and in water, complex **I** and most polymers achieved detectable CFU reduction against *P. aeruginosa*, whereas only cationic polymers reduced the bacterial load of *S. aureus* N315. Under saline conditions, polymers **16** and **19** stood out for their ability to nearly eliminate the bacterial load of both *P. aeruginosa* strains, while activity against *S. aureus* was largely abolished. Activity against *E. coli* DH5ɑ was generally comparable to that against *P. aeruginosa*, with the notable exception of polymer **16**, which showed substantially reduced activity against this species. This observation is consistent with the well‐known role of outer‐membrane composition and lipopolysaccharide architecture in modulating polymer–bacterial interactions and warrants further investigation in a strain‐specific follow‐up.

#### 2.3.3 Salinity, ASM, and Biofilm Activity

Two aspects of the antibacterial performance are particularly relevant to clinical translation, and were examined in dedicated follow‐up assays: activity as a function of ionic strength, and activity against biofilms.

Activity under variable NaCl concentrations was assessed at sub‐inhibitory doses of complex **I** and of polymers **16** and **17** (Figure S14). Compound **I** was strongly salt‐sensitive, with photodynamic efficacy diminishing at physiological ionic strengths. Polymer **16** showed intermediate sensitivity. Strikingly, polymer **17** exhibited a marked salt‐driven enhancement of its photodynamic activity, reaching maximum efficacy at 4× NaCl. This peculiar behaviour, unique to **17** within the library, may be related to its specific compositional features (R group, trithiocarbonate end groups; Table 1) and to possible surfactant‐like effects; further investigation is warranted, in particular given the clinical relevance of high‐salinity environments, such as hypertonic saline in cystic fibrosis therapy. Complementary tests in artificial sputum medium (ASM) confirmed the assay‐compatible mobility of the platform (see §2.2.3), with detectable aPDT activity retained upon partial dilution of the medium with water.

Antibiofilm activity was probed against two *P. aeruginosa* strains (the mucoid laboratory strain PA19 and the clinical CF isolate CH2678) using a TTC‐based kinetic assay measuring the time to reach a metabolic‐activity threshold post‐treatment (Figure 3C and Figure S15; longer times indicate greater delay of biofilm recovery). No dark toxicity was observed with any aPDT‐polymer. Under blue‐light irradiation, polymers **16** and **19** consistently emerged as the most active, significantly delaying biofilm recovery, though complete eradication was not achieved after a single treatment cycle. Comparative tests in saline confirmed the superior performance of **16** and **19** under saline conditions relevant to physiological environments. **18** did not exert any antibiofilm activity in any experimental condition.

#### 2.3.4 Biocompatibility in the Dark and Under Illumination

Given the reactive‐oxygen‐species‐generating character of the aPDT‐polymers under blue‐light irradiation, an assessment of host‐cell compatibility both in the dark and under the illumination protocol used for antibacterial assays is essential to establish the therapeutic window of the platform. Two complementary readouts were employed: haemolysis on rabbit erythrocytes as a first‐line indicator of membrane compatibility, and cell‐viability assays on two human epithelial cell lines relevant to respiratory applications (16HBE bronchial cells and A549 alveolar cells).

Haemolysis was assessed across the polymer library (**13**, **14**, **15**, **16**, **18**, **19**) and for the reference ruthenium complex **I** at concentrations spanning three orders of magnitude (0.5–1000 µg/mL), in the dark and under blue‐light irradiation at the illumination dose used for the antibacterial assays (Figure 4A). Under both conditions, haemolysis remained negligible across the entire concentration range. No polymer or reference compound induced more than 2% haemolysis up to 100 µg/mL, and haemolysis remained below approximately 3% at the highest concentration of 1000 µg/mL in the dark. Upon illumination, the overall response was essentially unchanged; a slight increase was observed for polymer **18** at 1000 µg/mL, reaching approximately 5%, while all other compounds remained below 2%. Even the highest values recorded are well below the 10% threshold commonly considered as the upper limit of acceptable haemolytic activity for biocompatible materials, and are reached only at concentrations approximately three orders of magnitude above the polymers' minimum inhibitory concentrations against the target bacterial species. Visual inspection of the assay plates confirms this quantitative result: erythrocyte pellets remain intact under all polymer treatments, and the corresponding supernatants remain colourless, in stark contrast to the fully red supernatants observed in the Triton X‐100 positive‐control wells.

**FIGURE 4:**
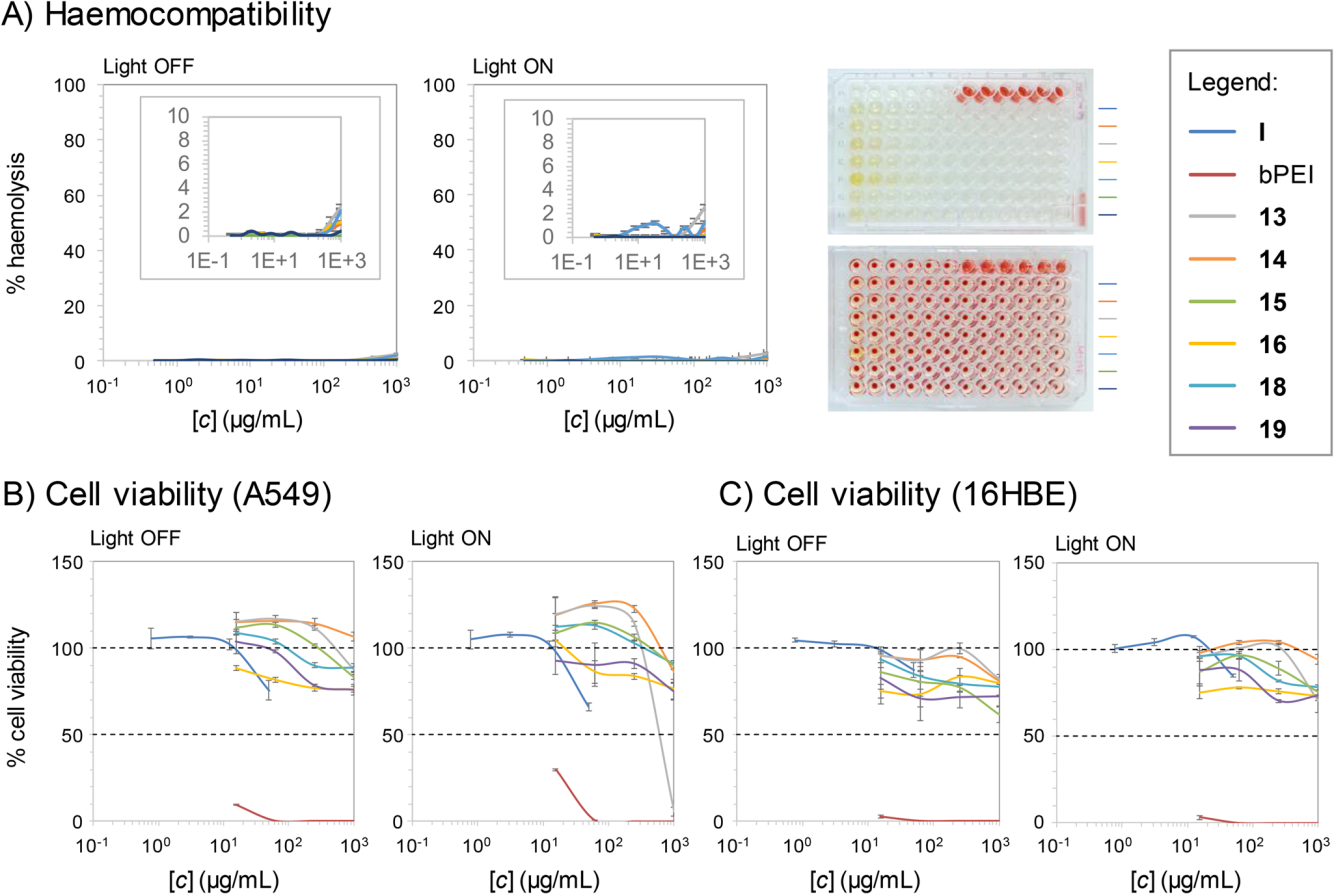
Biocompatibility of the aPDT‐polymer library and of the reference complex **I**, in the dark (OFF) and under blue‐light illumination (ON). (**Panel A**) Haemolysis of rabbit erythrocytes as a function of compound concentration (0.1–1000 µg/mL) in the dark (left) and after 30 min illumination (centre). Insets show the same data on an expanded y‐axis (0%–10%). Right: photographs of the assay plates after illumination, showing supernatants (upper plate) and erythrocyte pellets (lower plate). Complete haemolysis was defined using Triton X‐100 controls. (**Panel B**) Viability of A549 alveolar epithelial cells and (**Panel C**) of 16HBE bronchial epithelial cells as a function of compound concentration (0.1–1000 µg/mL), in the dark (left) and under illumination (right), quantified by intracellular ATP measurement (Vialight kit, Lonza). Branched polyethylenimine (bPEI) served as a positive cytotoxicity control. Dashed lines mark 100% (control viability) and 50% (cytotoxicity threshold). See Figure S16 for complementary cytotoxicity evaluation and the Supporting Information for experimental details. Data represent means ± SD of *n* = 3.

Cell viability was subsequently evaluated on 16HBE and A549 cells across the polymer library at concentrations from 15 to 1000 µg/mL, both in the dark (Light OFF) and under blue‐light irradiation (Light ON) at the same dose used for the antibacterial assays (Figure 4B,C). Branched polyethylenimine (bPEI) was included as a positive control for cationic‐polymer cytotoxicity. As expected, bPEI induced near‐complete cell death from the lowest concentration tested (∼15 µg/mL) in both cell lines and under both conditions, confirming the sensitivity of the assay. In contrast, all aPDT‐polymers displayed excellent tolerability across the concentration range: cell viability remained above 60% under all tested conditions with a single exception (see below), and typically between 70% and 100%, i.e., above the 50% cytotoxicity threshold across the full four‐log concentration range on both cell lines. Notably, illumination did not substantially compromise cell viability compared to the dark condition, indicating that ROS generated by the polymers under blue light does not translate into detectable damage of host respiratory cells at the doses examined. A single exception was observed on A549 cells under illumination at the highest concentration tested (1000 µg/mL), where polymer **13** displayed a marked drop in viability. The specific absence of surfactant in this polymer, compared to all others investigated (Table 1), suggests that the surfactant component may contribute to cellular tolerance, although this hypothesis warrants dedicated investigation.

Together, the haemolysis and cell‐viability data establish a comfortable, wide therapeutic window for the platform. The concentrations at which antibacterial activity is observed against the target pathogens (including MDR *P. aeruginosa* in biofilms) are one to two orders of magnitude below those at which any incipient host‐cell effect becomes detectable, and are three orders of magnitude below those at which haemolysis approaches the 10% acceptability threshold. This is a critical prerequisite for the intended aPDT applications, where local light‐activated bacterial killing must be achieved without compromising the surrounding host tissue.

### 2.4 Averaged Structure–Activity Relationships

#### 2.4.1 Motivation and Methodology

The diversity of compositions, physicochemical characteristics, and antimicrobial profiles observed across the library invites a systematic analysis of the relationships between polymer descriptors and biological performance. A multivariate approach was adopted to identify compositional and physicochemical axes that co‐vary with antimicrobial activity, without imposing a priori assumptions about which parameters matter most. Fifteen descriptors were assembled per polymer — compositional variables (mol% HEAAm, Ru, surfactant, and AA), molecular characteristics (*M*_n_, charge, size), photophysical parameters (A_450_, fluorescence, ^1^O_2_ output), and antimicrobial readouts (MIC against PA, EC, and SA) — and jointly analysed by hierarchical clustering and principal‐component analysis (PCA) after z‐score normalisation. Given the size of the library (seven polymers) and the number of descriptors, the multivariate analysis is best interpreted as exploratory: it highlights compositional axes worth pursuing, without providing statistical proof of causation. Details of the methodology are provided in the Supporting Information.

#### 2.4.2 Clustered Heatmap and Principal Component Analysis

The clustered heatmap (Figure 5A) reveals three distinct polymer groups. Group I contains the cationic FRP polymers **13**, **14**, and **15**, characterised by low AA content, low‐to‐moderate Ru loading, low charge magnitude with positive sign, moderate ^1^O_2_ output, and strong activity against *S. aureus* in water. Group II contains the anionic AA‐rich polymers **16**, **17**, and **19**, characterised by ∼20 mol% AA, moderate Ru loading, negative charge, moderate ^1^O_2_ output, and strong activity against *P. aeruginosa* including under saline conditions (this SAR clustering groups polymers according to their overall compositional and functional profile across all 15 descriptors; polymer **17**, for example, groups with **16** and **19** on this basis despite its more heterogeneous colloidal behaviour—see §2.2.2). Group III contains polymer **18**, which stands out as an outlier: the highest Ru loading of the library, the largest hydrodynamic size (>1 µm), the highest fluorescence, and the highest ^1^O_2_ production, yet remarkably weak antibacterial activity across all strains.

**FIGURE 5:**
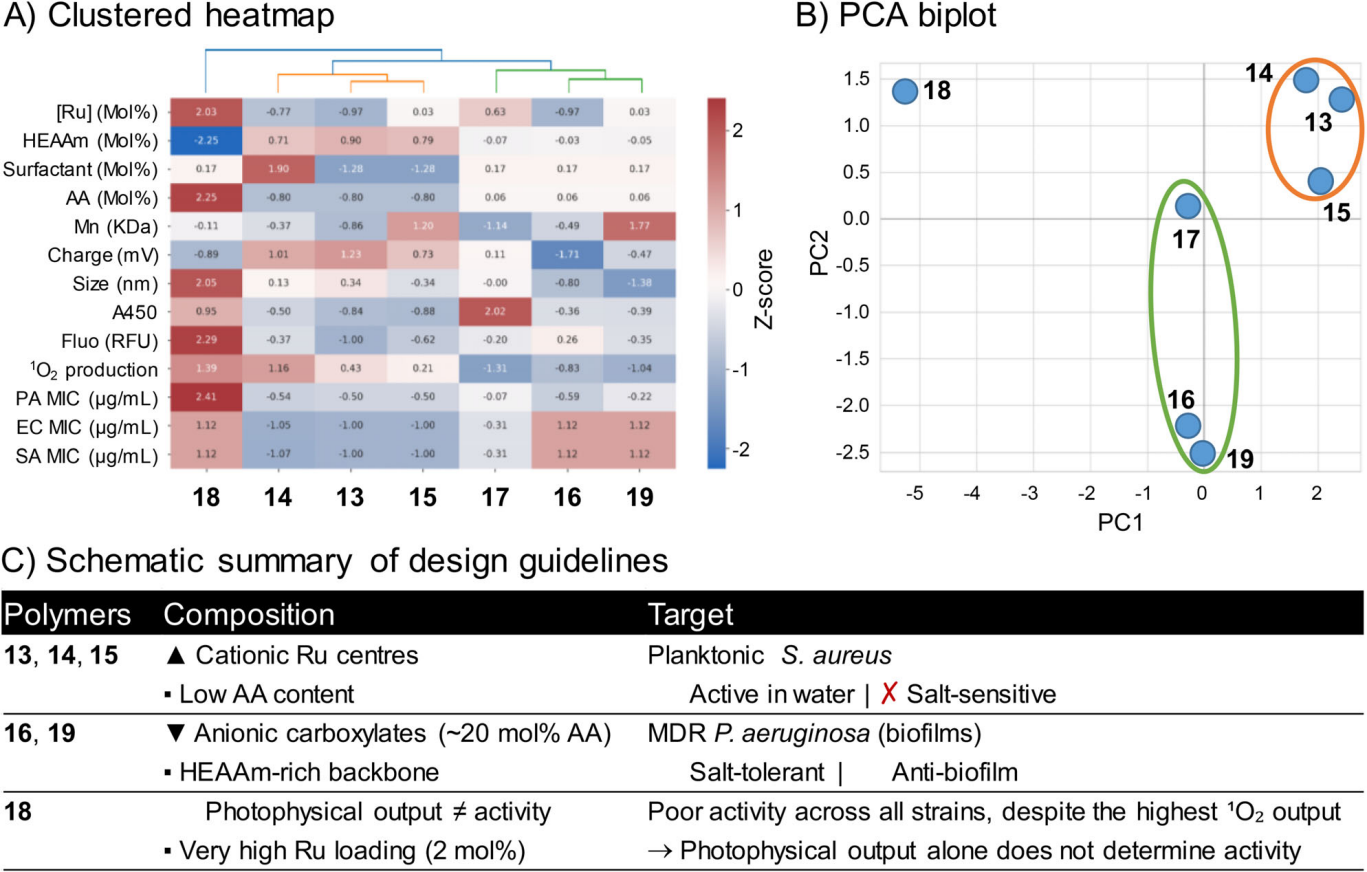
Multivariate structure–activity analysis of the aPDT‐polymer library. (**Panel A**) Clustered heatmap of z‐score‐normalised physicochemical, photophysical, and antimicrobial descriptors across the polymer library. Hierarchical clustering (Ward's linkage, Euclidean distances) identifies three polymer clusters. Descriptors correspond to built‐in compositions (%HEAAm, %Ru, %surfactant, %AA), molecular characteristics (*M*_n_ from SEC; charge and hydrodynamic size from DLS and electrophoresis), photophysical parameters (A_450_, fluorescence, ^1^O_2_ production), and MIC values against *P. aeruginosa*, *S. aureus*, and *E. coli* (Table 2). (**Panel B**) Principal‐component analysis (PCA) biplot of the same standardised dataset. The three clusters identified in Panel A are visible in PC1–PC2 space: cationic FRP polymers (**13**, **14**, **15**; orange ellipse), anionic AA‐rich polymers (**16**, **17**, **19**; green ellipse), and polymer **18** as a distinct outlier (top‐left). (**Panel C**) Design‐guidelines summary derived from the multivariate analysis; see §2.4.3 and §2.4.4 for detailed interpretation. See the Supporting Information (§S1.12) for methodology details.

Principal‐component analysis (PCA; Figure 5B) confirms this three‐cluster structure in reduced dimensional space. The first two components (noted PC1 and 2) together capture the dominant compositional and functional variance across the library, and the three clusters are well‐separated in PC1–PC2 space. Group I polymers cluster together in the upper‐right region, driven by their cationic character and MIC signatures against Gram‐positive bacteria. Group II polymers cluster in the lower‐central region, driven by AA content, negative charge, and MIC signatures against Gram‐negative strains, particularly under saline conditions. Polymer **18** projects into its own region, driven by its extreme Ru loading and photophysical output.

Two general points emerge from this analysis. First, polymer architecture (encoded in the interplay between AA content, surfactant content, and Ru loading) rather than ruthenium loading alone, is the dominant determinant of antimicrobial activity across the library. Second, photophysical performance (^1^O_2_ generation, fluorescence) does not linearly map onto antimicrobial activity: polymer **18**, the strongest ^1^O_2_ producer, is among the weakest antibacterial, indicating that photophysical output is a necessary but not sufficient condition for antimicrobial efficacy.

#### 2.4.3 Identified Design Guidelines

Two composition‐based design guidelines emerge from the multivariate analysis and are supported by the findings of the antibacterial performance (Figure 5C).

*Guideline 1*: For activity against Gram‐positive planktonic cells (particularly *S. aureus* N315 in water), cationic FRP compositions with low AA content (**13**, **14**, and **15**) are the most effective. Their activity is potentially dominated by electrostatic engagement with the negatively charged bacterial envelope and is essentially abolished under saline conditions, where charge interactions are screened.

*Guideline 2*: For activity against Gram‐negative pathogens (particularly MDR *P. aeruginosa*, including in biofilms and under saline conditions of clinical relevance), anionic AA‐rich compositions with a HEAAm‐rich backbone and moderate Ru loading (**16** and **19**) are the most effective. Under saline conditions, these polymers retain (and in some cases enhance) their photodynamic activity, indicating that their antibacterial action does not rely primarily on electrostatic interactions and may instead depend on aggregation‐driven or bridging mechanisms discussed in §2.4.4.

Both guidelines should be interpreted as correlative associations between batch‐averaged compositional descriptors and activity, rather than causal attributions to specific molecular features. The chain‐to‐chain compositional heterogeneity intrinsic to FRP, and the partial‐substitution character of PPM, both introduce a distribution of local architectures within each batch (§2.1.6), which cannot be resolved with the available characterization methods. What the multivariate analysis identifies is therefore a set of architectural signatures (combinations of composition, charge, and Ru content) that co‐vary with antibacterial performance in a reproducible way across the library, and that provide practical guidelines for the design of next‐generation aPDT‐polymers.

#### 2.4.4 Notable Behaviors: The Cases of Polymers 16 and 18

Two polymers of the library warrant particular attention. (1) Polymer **16** exhibits an unexpected concentration dependence of its antibacterial activity, with efficacy decreasing at higher concentrations against certain strains, a behaviour reproducible across independent synthesis batches (Figure S12). This is consistent with concentration‐dependent aggregation or PIC formation (§2.2.4), which may reduce the accessibility of the ruthenium centres or alter their photophysical behaviour at high polymer densities. Its salt‐enhanced biofilm activity, retained association with biofilms in saline conditions, and species‐selective activity (marked efficacy against *E. coli*) all point to compositional and colloidal features whose mechanistic dissection warrants a dedicated follow‐up study. (2) Polymer **18**, at the opposite compositional extreme, exhibits the paradoxical combination of the highest photophysical output of the library with one of the weakest antibacterial performances. Its large hydrodynamic size (>1 µm, Figure 2A) and its very high AA content (71 mol%) suggest that its ruthenium centres, while spectroscopically the most productive of ^1^O_2_ per unit mass, are physically inaccessible to the bacterial targets (either through internal PIC‐driven collapse of the polymer coil or through the formation of large aggregates that fail to associate with bacterial surfaces). Polymer **18** thus illustrates that photophysical performance and antibacterial efficacy can be decoupled by architectural factors, an important design lesson for future aPDT‐polymer platforms.

## 3 Conclusions

This work establishes a modular dual‐strategy platform for the synthesis of functionalised polymers, in which free‐radical copolymerisation (FRP) and post‐polymerisation modification (PPM) are deployed as orthogonal routes within a single design framework. This strategy was used to combine a hydrophilic backbone with a tuneable content of hydrophobic side chains and charged groups, together with Ru photoactive agents as PS for aPDT. To the best of our knowledge, this constitutes the first systematic side‐by‐side implementation of FRP and PPM applied to a Ru‐photopolymer library, allowing direct evaluation of how the synthetic strategy can be used to target the biological performance of the resulting material. The obtained aPDT‐polymers are water‐dispersible, photochemically stable, and non‐(photo)cytotoxic towards human bronchial epithelial cells up to concentrations exceeding 1 mg/mL, while exhibiting potent antibacterial activity (against both planktonic and biofilm forms) at much lower concentrations upon blue‐light irradiation. Haemolysis remained below 5% at 1000 µg/mL even under illumination, well within the range considered acceptable for biocompatible materials. Structure–activity analysis across the library identifies overall polymer architecture, rather than ruthenium loading, as the dominant determinant of activity: cationic compositions are most effective against planktonic *S. aureus*, whereas anionic compositions combining ∼20 mol% acrylic acid with a HEAAm‐rich backbone (polymers **16** and **19**) are most effective against MDR *P. aeruginosa*, including in biofilms and under saline conditions of clinical relevance. Activity against *E. coli* was generally comparable to that against *P. aeruginosa*, with the notable exception of polymer **16**, which showed substantially reduced activity against this species. In summary, beyond the specific Ru complexes investigated here, the platform is extensible to other types of PS and polymer–metal architectures, providing a versatile and rationally tuneable basis for the design of next‐generation polymer‐based photoactive antimicrobials and, more broadly, for functional polymer–metal hybrid materials.

## 4 Experimental Section

Full protocols, characterisation data, and statistical analyses are provided in the Supporting Information.

### 4.1 Polymer Synthesis

Free‐radical copolymerisations (FRP) were performed with AIBN as thermal initiator in dioxane/acetone or DMSO at 65–70°C for 24 h under argon. Postpolymerisation modification (PPM) was carried out by aminolysis of polyPFPA‐*ran*‐Surf‐EH3 with amine‐functionalised Ru complexes in DMF and triethylamine, followed by treatment with ethanolamine to consume residual pentafluorophenyl esters. All polymers were purified by precipitation and dialysis (2 kDa MWCO) and freeze‐dried before use.

### 4.2 Physicochemical and Photophysical Characterisation

UV‐visible absorption, fluorescence emission, dynamic light scattering, size‐exclusion chromatography with UV detection at 280 nm, agarose gel electrophoresis, and singlet‐oxygen detection (SOSG) were performed in water and in 0.9% NaCl. Blue‐light irradiations used a calibrated custom LED setup [32].

### 4.3 Antibacterial and Biocompatibility Assays

MIC values, CFU reductions, biofilm activity (TTC‐based kinetic assay), haemolysis (rabbit erythrocytes), and cell viability (16HBE and A549 cells; intracellular ATP quantification and measurements of adenylate kinase release) were assessed in the dark and under blue‐light irradiation.

### 4.4 Multivariate Analysis

Descriptors were z‐score‐normalised. Hierarchical clustering used Ward's linkage on Euclidean distances; principal‐component analysis was performed on the same standardised dataset.

## Author Contributions

AB, JF, JG, GL, FT, TLG, UJ: Conceptualization. AB, JF, JG, GL, FT, TLG, UJ: methodology. AB, JF, MB, JG, AN, TM, GL, FT, TLG, UJ: validation. AB, JF, MB, JG, AN, GL, FT, TLG, UJ: investigation. AB, JF, MB, JG, TM, GL, FT, TLG, UJ: resources. AB, JF, MB, JG, AN, GL, FT, TLG, UJ: data curation. AB, JF, JG, TM, GL, FT, TLG, UJ: writing the original draft preparation. AB, JF, MB, JG, AN, TM, GL, FT, TLG, UJ: writing, review and editing. AB, JF, MB, JG, AN, TM, GL, FT, TLG, UJ: visualization. GL, TLG, UJ: supervision. GL, TLG, UJ: project administration. GL, FT, TLG, UJ: funding acquisition. All authors have read and agreed to the published version of the manuscript.

## Funding

This work was supported by grants from ANR/BMBF (TARGET‐THERAPY project; ANR grant number: ANR‐20‐AMRB‐0009, RPV21103NNA; BMBF Förderkennzeichen: 16GW0342). Financial supports were also obtained from Inserm International Research Project (PHOENIX), the “Association de Transfusion Sanguine et de Biogénétique Gaétan Saleün” (France), “Banque Populaire Grand Ouest”, “Fondation Groupama”, “PLB Muco Callac”, and the “Région Bretagne” (France). Ali Balasini received a PhD fellowship from BMBF (TARGET‐THERAPY project; BMBF Förderkennzeichen: 16GW0342). Margaux Bonnardot holded a PhD fellowship jointly awarded by the French Ministry of Higher Education, Research and Innovation (Ministère de l'Enseignement supérieur, de la Recherche et de l'Innovation, Paris, France) and the Région Bretagne (France). Jérémy Godard was awarded a postdoctoral fellowship funded by ANR (TARGET‐THERAPY project; ANR grant number: ANR‐20‐AMRB‐0009, RPV21103NNA).

## Conflicts of Interest

The authors declare no conflicts of interest.

## Supporting information




**Supporting File**: marc70426‐sup‐0001‐SuppMat.pdf.

## Data Availability

The data that support the findings of this study are available from the corresponding authors upon reasonable request.
