# Supplementary material for: Modular Polymers With Tuneable Charge and Amphiphilicity for Effective Antimicrobial Photodynamic Therapy
Source: Macromol Rapid Commun. 2026 Sep 10;47(21):e70426. doi: 10.1002/marc.70426 (PMC13741393; doi:10.1002/marc.70426)
Supplement: Supplementary file 1 [file MARC-47-e70426-s001.pdf]

RESEARCH ARTICLE

Modular Polymers with Tuneable Charge and Amphiphilicity for Effective Antimicrobial Photodynamic Therapy

Ali Balasini[1] | Jannik Fryn[1] | Margaux Bonnardot[2] | Jérémy Godard[3] | Tristan Montier[2,4] | Gilles Lemercier[3,5] | Franck Thétiot[6*] | Tony Le Gall[2*] | Ulrich Jonas[1*]

[1] Macromolecular Chemistry, Department of Chemistry and Biology, University of Siegen, 57076 Siegen, Germany

[2] Univ Brest, Inserm, EFS, UMR 1078, GGB, F-29200 Brest, France

[3] ITODYS - UMR CNRS 7086, Univ. Paris Cité, 75013 Paris, France

[4] CHU de Brest, Service de Génétique Médicale et Biologie de la Reproduction, Centre de Référence des Maladies Rares "Maladies Neuromusculaires", 29200 Brest, France

[5] Univ. Reims Champagne-Ardenne

[6] Univ. Brest, CNRS, UMR 6521, CEMCA, 29238 Brest, France

Correspondence: Franck Thétiot (thetiot@univ-brest.fr) | Tony Le Gall (tony.le-gall@inserm.fr) | Ulrich Jonas (jonas@chemie.uni-siegen.de)

Funding: This work was supported by grants from ANR/BMBF (TARGET-THERAPY project; ANR grant number: ANR-20-AMRB-0009, RPV21103NNA; BMBF Förderkennzeichen: 16GW0342) and Inserm (PHOENIX International Research Project, 2025 IRP edition)

Keywords: antimicrobial polymers; photodynamic therapy; post-polymerisation modification; ruthenium complexes; structure-activity relationships; polymer–metal hybrids

Table of Content

S1 | Supplementary Experimental Section 3
S1.1 | Chemistry Section 3
S1.1.a | Chemical and Reagents 3
S1.1.b. | Analytical Techniques 3
S1.2 | Biological Section 4
S1.2.a | Reagents, Media and Devices 4
S1.2.b | Illumination Device and Protocol 4
S1.2.c | UV-Visible Absorption and Fluorescence Measurements 4
S1.2.d | Electrophoretic Assays 4
S1.2.e | SOSG Assays 5
S1.2.f | Bacterial Assays 5
S1.2.g | Eukaryotic Cell Assays 6
S1.2.h | Heatmaps and Hierarchical Clustering Dendrograms 7
S1.2.i | Statistical Analyses 7
S2 | Detailed Synthesis Part 8
S3 | Supplementary NMR Spectra 19
S4 | Supplementary GPC data 30
S5 | Supplementary Tables 44
S6 | Supplementary Figures 45
S7 | Supplementary References 62

S1 | Supplementary Experimental Section

S1.1 | Chemistry Section

S1.1.a | Chemical and Reagents

Solvents used were ethanol, ethyl acetate, hexane, methanol (98.5%, VWR), chloroform (99%, VWR), and dichloromethane (99.9%, Fisher Scientific) of technical purity, unless specified otherwise. Distilled water was obtained from a MilliQ apparatus (resistivity σ = 18.2 MΩ cm). Absolute ethanol (99.9%, Sigma Aldrich) was stored under inert conditions. Dioxane (98%, Roth Chemicals) was freshly distilled (over sodium). THF (99.9%, Fisher Scientific) was used as received. Other dried solvents were prepared through refluxing for one day under nitrogen over appropriate drying agents (sodium-naphthalene and benzophenone for THF, sodium-benzophenone and calcium hydride for diethyl ether, calcium hydride for acetonitrile, magnesium and iodine for methanol, sodium for ethanol) and degassed before use. These solvents were stored in glass ampoules under argon. Monomers and initiators were acrylic acid (99%, Sigma-Aldrich), azobisisobutyronitrile (AIBN) (99%, Acros Organics). Other reagents were carbon disulfide (99%, Sigma-Aldrich), formic hydrazide (98%, Alfa Aesar), triethyl orthoformate (98%, Alfa Aesar), 3-aminopropan-1-ol (99%, Alfa Aesar), triphenylphosphine flakes (99%, Alfa Aesar), diisopropyl azodicarboxylate (94%, Alfa Aesar), hydrazine monohydrate (>98%, Alfa Aesar), phthalimide (99%, Alfa Aesar), L-ascorbic acid (99%, Alfa Aesar), 4-(dimethylamino)pyridine (99.9%, Acros Organics), aqueous ammonia (25%, Roth Chemicals), *N*,*N′*-dicyclohexylcarbodiimide (99%, Sigma-Aldrich), 2-bromo-2-methylpropionic acid (>98%, Alfa Aesar), 1-ethyl-3-(3-dimethylaminopropyl)carbodiimide (99%, Roth), triethylamine (99%, Alfa Aesar), 1-dodecanethiol (>98%, Sigma-Aldrich), *N*-hydroxysuccinimide (98%, Alfa Aesar), iron(II) tetrafluoroborate hexahydrate (99%, Sigma Aldrich). Carbon disulfide, 2,2′-azobis[2-(2-imidazolin-2-yl)propane] dihydrochloride, hydrazine monohydrate, and iron(II) tetrafluoroborate hexahydrate were stored under inert atmosphere and light exclusion at 0-4 °C.

S1.1.b. | Analytical Techniques

The molar mass distribution and dispersity of the polymers were measured using a gel permeation chromatography (GPC) system consisting of a PSS-SECcurity system (Agilent Technologies 1260 infinity), equipped with a PSS GRAM 100 Å column and a 10 µm particle size pre-column. A solution of DMAc with 0.1% LiBr was used as eluent. A sample volume of 20 µL was injected at a temperature of T = 60 °C, with a flow rate of 1 mL/minute. PMMA standards (Polymer Standard Service, Mainz) were used for calibration. NMR spectra were recorded on a Jeol ECZ 500 spectrometer. Unless otherwise stated, ^{1}H NMR measurements were carried out at a frequency of 500 MHz, while ^{13}C NMR measurements were performed at 125 MHz (corresponding to a 11.7 T magnet). All spectra were recorded at room temperature (ca. T = 20-25 °C). The chemical shifts were reported in parts per million (ppm), using the signals of the solvent residual peak of the corresponding deuterated solvents as internal standards ($CDCl_3$: ^{1}H: 7.26 ppm, ^{13}C: 77.2 ppm; acetone-d_6: ^{1}H: 2.05 ppm, ^{13}C: 29.84 ppm; DMSO-d_6: ^{1}H: 2.50 ppm, ^{13}C: 39.52 ppm; D_2O: ^{1}H: 4.79 ppm). Signal assignments were made using MestReNova software. Size and zeta potential measurements were conducted at 25 °C using a Zetasizer Nano ZS (Malvern Instruments). Each solution was diluted in Milli-Q water to a final volume of 1 mL, with the concentration adjusted to 1 mg/mL. For analysis, 700 µL were transferred into a Malvern DTS10760 cell for size and zeta potential measurements. The pH of aPDT-polymer stock solutions (5 mg/mL in water) was estimated using Universal Indicator Paper (pH 1-14; Clearline, Duscher).

S1.2 | Biological Section

S1.2.a | Reagents, Media and Devices

The related bare complex Ru(Phen)$_3^{2+}$, 2 PF_6^-, hereafter numbered **I**, was purchased from StremChemicals (CAS. 60804-75-3, StremChemicals, Newburyport, MA, USA). Stock solutions were prepared in dimethyl sulfoxide (DMSO, 99.9% purity; Sigma-Aldrich) at a concentration of 5 mg/mL. Unless otherwise stated, **I** was tested with cells or bacteria at concentrations up to 50 µg/mL, ensuring that the final DMSO content in the cultures did not exceed 1%, in line with recommendations to keep solvent levels at or below this threshold.[1,2] Base polymers (devoid of Ru) and aPDT-polymers were dispersed in water at 5 mg/mL. Saline solution (hereafter noted 1× NaCl, referring to a solution at physiological ionic strength) corresponded to 0.9% (w/v) sodium chloride in water (Versol, London, UK). Artificial sputum medium (ASM) was prepared as previously reported.[3,4]

S1.2.b | Illumination Device and Protocol

Illumination treatments were performed using a home-made illumination device (Figure S1), which we have reported earlier.[3] This custom-built experimental system was designed to perform simultaneous bidirectional illumination with two wide (310 x 310 mm) panels, each incorporating 225 14W pure blue LEDs (wavelength: 450-470 nm), facing each other. LED panels were placed on both sides of a plate that could be used to expose multi-well plates in a central exposure area (CEA). A PM160 wireless handheld power meter (Thorlabs, Bergkirchen, Germany) was used to measure light transmittance and check the homogeneity of the illumination field). Unless otherwise stated, light treatment was performed at room temperature (RT, ca. T = 20-25°C), as follows (Figure S1). Fifteen minutes after the addition of reagents, the samples to be irradiated were placed in the CEA, and then top and bottom exposure to LED light was performed, the whole procedure being carried out at room temperature (RT). A typical irradiation cycle lasted no more than 30 minutes in total, which was found to be sufficient to achieve PDT effects in most cases, without inducing any noticeable heating.[3] For some selected experiments, the commercially available W3 LED Irradiation System (Live Cell Instrument) was additionally employed to further validate results reproducibility. The treatment effects were assessed by comparing samples subjected to illumination with samples of the same composition protected from light. The above-mentioned conditions were denoted as "ON" (light-treated condition) and "OFF" (non-irradiated control), respectively.

S1.2.c | UV-Visible Absorption and Fluorescence Measurements

All hereinafter fluorescence and absorption measurements were performed using the multiplate reader Mithras2 LB 943 (Berthold Technology, Thoiry, France). For fluorescence measurements, 100 µL of reagents at the indicated concentration were introduced per well of black 96-well plates (Falcon). Measurements were done with excitation at 460 ± 15 nm and emission readings at 590 ± 22 nm. For UV-visible absorption, UV/vis transparent flat-bottom multi-well plates (Greiner Bio-One) were used, and absorption was measured from 230 to 630 nm (increment = 5 nm) at 25 °C.

S1.2.d | Electrophoretic Assays

Unless otherwise stated, the reagents were subjected to electrophoresis using 0.5 or 1.0% agarose gel prepared with TBE buffer (Fisher Scientific), stained or not with ethidium bromide (EtB, at 0.5 µg/mL). After loading the samples in the wells of the gel, electrophoresis was conducted in 1× TBE buffer for 15 minutes at 100 V. Finally, a picture of the gel was taken

using a UV trans-illuminator equipped with 8W 312 nm emitting lamps (Fire-Reader V10 Plus, UVITEC).

S1.2.e | SOSG Assays

The singlet oxygen sensor green (SOSG) fluorescent probe (Fisher Scientific, Eugene, OR, USA) was used to quantify $^1O_2$, following the manufacturer's instructions. Tests were done in 100 mM HEPES pH 7.4 with the reagents supplemented with 5 µM SOSG. Following incubation at room temperature for at least 15 minutes in the dark, fluorescence was measured with excitation at 504 ± 6 nm and emission readings at 525 ± 12 nm, before and after light treatment. Sodium azide (Sigma-Aldrich) was employed as a $^1O_2$ scavenger in some experiments to confirm singlet oxygen production.

S1.2.f | Bacterial Assays

A series of clinically-relevant *S. aureus* or *P. aeruginosa* strains were used in this study: the *S. aureus* Newman, [5] the methicillin-resistant *S. aureus* (MRSA) N315,[6] the *P. aeruginosa* (Schroeter) Migula (ATCC® 19660™, hereafter noted PA19) and the CF originating *P. aeruginosa* CH2678 (Helmholtz, Bactome collection; https://bactome.helmholtz-hzi.de/). In addition, an *E. coli* strain was included as another Gram-negative organism for some of the tests. All bacterial routine handling was conducted with Luria Bertani (LB) broth at 37 °C. Detailed information about each strain is available in the Supplementary Materials (Table S1).

To assess the interaction of aPDT-polymers with planktonic cells, bacteria were first pre-cultured overnight and subsequently subcultured the following day in 10 mL of LB medium, followed by incubation at 37 °C for 6 hours. Cultures were centrifuged at 1,000 g for 10 minutes at 25 °C, and the supernatant was discarded. Pellets were washed with 10 mL of sterile water or 0.9% NaCl, centrifuged again under the same conditions, and the supernatant was removed identically. The final pellet was resuspended in 5 mL of water or 0.9% NaCl, and optical density (OD) was measured at 600 nm. OD was adjusted to 0.6 for Gram-negative and 1.2 for Gram-positive strains, corresponding to ca. 5×10^8 CFU/mL. Subsequently, 150 µL of either water or bacterial dilution was added to 50 µL of PS, so as to reach to final concentrations of 5 µg/mL for the bare ruthenium complex **I** and 50 µg/mL for aPDT-polymers. Plates were incubated at 37 °C for 1 hour, then centrifuged at 1,000 g for 10 minutes at 25 °C. From each well, 100 µL of supernatant was carefully transferred into a black 96-well plate with a transparent bottom for fluorescence reading at 460/590 nm.

To assess the interaction of aPDT-polymers with biofilms, precultures of *P. aeruginosa* were prepared the day before the experiment. On the following day, 200 µL of inoculated LB medium were distributed into each well of a 96-well plate. The plates were incubated under static conditions at 37 °C to allow biofilm formation. After incubation, the biofilms were gently washed three times with sterile water. A volume of either the Ru compound (50 µg/mL) or the PDT-polymers (500 µg/mL) was then added to the designated wells. Samples were incubated at RT. After 30 minutes, reagents were carefully removed and another washing step, again performed three times with sterile water, followed. As controls, biofilms not exposed to any reagent in the previous step were treated with Ru or PDT-polymer and the plates were allowed to dry completely at room temperature. Fluorescence measurements (at 460 ± 15 nm and emission readings at 590 ± 22 nm) were subsequently recorded, and images were acquired under UV transillumination. Biofilms were finally stained with crystal violet, and absorbance was measured at 595 nm.

To determine the minimal inhibitory concentration (MIC), 50 µL of serial dilutions of the PS were combined with 50 µL of a bacterial inoculum, adjusted to approximately 5×10^5 CFU/mL (in accordance with EUCAST guidelines), using either water or 0.9% NaCl as diluent.

Concentrations ranging from 0.5 to 500 µg/mL for the aPDT-polymers and from 0.05 to 50 µg/mL for compound **I** were tested with bacteria suspended in water or saline. Before light exposure, 5 µL from each well of the 96-well plate were plated onto LB agar to serve as pre-treatment controls. The microplates were then irradiated for 30 minutes, followed by a second spotting of 5 µL from each well onto fresh LB agar plates. All agar plates were incubated overnight at 37 °C and subsequently photographed to document bacterial growth. In parallel, 90 µL of 2× LB medium were added to each well, and after 24 hours of incubation at 37 °C under constant shaking, bacterial growth in the wells was evaluated by measuring the absorbance at 600 nm.

For the determination of bacterial load reduction, a single colony of each bacterial strain was inoculated into 5 mL of LB medium and incubated overnight at 37 °C with shaking at 220 RPM. The following morning, 10 µL of this culture was transferred into 10 mL of fresh LB and incubated for 6 h to allow the bacteria to reach mid-exponential phase. Bacteria were then collected by centrifugation at 1,000 g for 10 minutes at 4 °C, the supernatant was discarded, and the pellet was washed with 10 mL of water or saline. After a second centrifugation under the same conditions and removal of the supernatant, the pellet was resuspended in water or saline. The optical density at 600 nm was measured, and the suspension was adjusted with water or NaCl to an OD of 1.2 for Gram-positive bacteria or 0.6 for Gram-negative bacteria. The bacteria were then mixed with the test compounds in water or saline and incubated at RT for 15-20 minutes to allow interactions to occur. Light treatment was applied once for 30 minutes. Colony-forming unit (CFU) enumeration was performed considering kinetic growth and 10-fold serial dilutions of the bacterial suspensions, as previously reported.[2] Non-irradiated bacteria were used as controls to assess any possible dark toxicity of the compounds.

For aPDT assays using biofilms, precultures of *P. aeruginosa* were prepared the day before the experiment. On the following day, 200 µL of inoculated LB medium were distributed into each well of a 96-well plate. The plates were incubated under static conditions at 37 °C to allow biofilm formation. The medium covering biofilms was carefully eliminated, and washing was repeated three times with water. Biofilms were then exposed with a given compound (either in water or saline, i.e., 0.9% NaCl) followed with or without blue LED light irradiation for 30 minutes (single continuous irradiation). Biofilms were then cultivated for several hours in the presence of LB supplemented with the redox indicator triphenyl tetrazolium chloride (TTC). In living cells, TTC is enzymatically reduced by various dehydrogenases into triphenyl formazan (TPF), resulting in a red product (TPF) from a colourless substrate (TTC). Therefore, the detection of a red colouration indicates the viability of bacteria in biofilms. The kinetic of red colouration (i.e., TTC conversion to TPF) reflects the overall metabolic activity of a given biofilm, which correlates with the number of viable bacteria present.

S1.2.g | Eukaryotic Cell Assays

The potential side-effects of aPDT-polymers were evaluated using two (one cancerous and one immortalized) human-derived epithelial cell lines (in the absence of any exposure to blue light): A549 (bronchial alveolar type II epithelial cells derived from human pulmonary carcinoma; ATCC No. CCL-185) and 16HBE 14o− (human bronchial epithelial cells; University of Vermont, Burlington, VT, USA [7]). The first was grown in Dulbecco's Modified Eagle Medium (DMEM) and the second in Minimum Essential Medium (MEM), both supplemented with 100 units/mL penicillin, 100 µg/mL streptomycin, and 10% heat-inactivated foetal bovine serum (FBS). Incubation was carried out at 37 °C, in a humidified atmosphere containing 5% CO_2. DMEM, MEM, FBS, penicillin, and streptomycin were purchased from Invitrogen. The day before the assays, cells were seeded in 96-well plates (Falcon) using 40,000 and 25,000 cells

per well for 16HBE and A549, respectively. After incubation for 24 hours, the culture medium was replaced and supplemented with a given reagent to test concentrations ranging from 0.5 μg/mL to 1 mg/mL. As controls, a range of concentrations of DMSO (solvent used for **I**) and the branched 25 kDa Polyethylenimine (bPEI, Sigma) were tested in parallel. After incubation for 24 h, the culture medium in each well was carefully removed, and cells were disrupted with the passive lysis buffer (Promega). Commercially available assays, either enzymatic or colorimetric in nature, were employed to evaluate the potential cytotoxic effects associated with the reagents under investigation: the Vialight kit (Lonza) was used to quantify the ATP content of the cells, which is a reporter of cell proliferation and viability, while the Toxilight kit (Lonza) was used to measure the activity of the enzyme adenylate kinase (AK) in the supernatant of the cells. The AK normally remains in the cytoplasm of healthy cells, so its presence in the culture medium can be used to estimate membrane injuries that cause the enzyme to leak out. The total protein content was also determined using the BCA assay kit (Interchim). Protocols were conducted as recommended by manufacturers. In addition, haemolysis assay was conducted with fresh erythrocyte suspension, derived from defibrinated rabbit blood (TCS Biosciences Ltd) washed in isotonic buffer (i.e., 1X PBS). Test samples, including controls, were added to a microplate (100 μL per well) followed by an equal volume of the red blood cells (RBC) suspension. The plate was sealed and incubated at 37 °C for one hour to allow interaction between RBC and the reagents. After incubation, the plate was centrifuged, and the supernatant was transferred to a new plate for absorbance measurement at 540 nm, which quantifies the release of haemoglobin as an indicator of cell lysis. Positive and negative controls were employed to interpret the extent of haemolysis: Triton X-100 (final 1 %) served as the positive control representing complete lysis and 1X PBS as the negative control representing no lysis, allowing normalized comparison of haemolytic activity.

S1.2.h | Heatmaps and Hierarchical Clustering Dendrograms

Heatmaps and hierarchical clustering dendrograms were generated using standardized numerical matrices derived from the physicochemical and biological parameters of the polymer library (Table S2). Prior to analysis, missing values were imputed using the mean of the corresponding variable, and categorical thresholds (e.g., values reported as “>500”) were replaced with their upper numerical limit to enable quantitative processing. All variables were then normalized using z-score scaling to ensure equal weighting across different measurement ranges. Pairwise distances between samples were computed using Euclidean distance, and hierarchical clustering was performed using Ward’s minimum variance method. Clustered heatmaps were produced by simultaneously applying hierarchical clustering to both rows (variables) and columns (samples), allowing visualization of similarity patterns across the dataset. Dendrograms were extracted directly from the hierarchical clustering results to illustrate the relationships between polymers based on their multivariate profiles.

S1.2.i | Statistical Analyses

For most assays, tests were performed in technical triplicates (n = 3), and at least two independent experiments were conducted (N ≥ 2). The results presented correspond to the mean and standard deviation (mean ± SD) for all variables. Data were compared using Student’s t-test processed with Prism software version 6.00 (GraphPad, San Diego, CA, USA). Statistically significant differences were denoted as follows: ***, p-value ≤ 0.001; **, p-value ≤ 0.01; *, p-value ≤ 0.05. Non-significant differences were noted as “ns”.

S2 | Detailed Synthesis Part

Synthesis of *cis*-[bis(1,10-phenanthroline)dichlororuthenium(II)] (**1**):

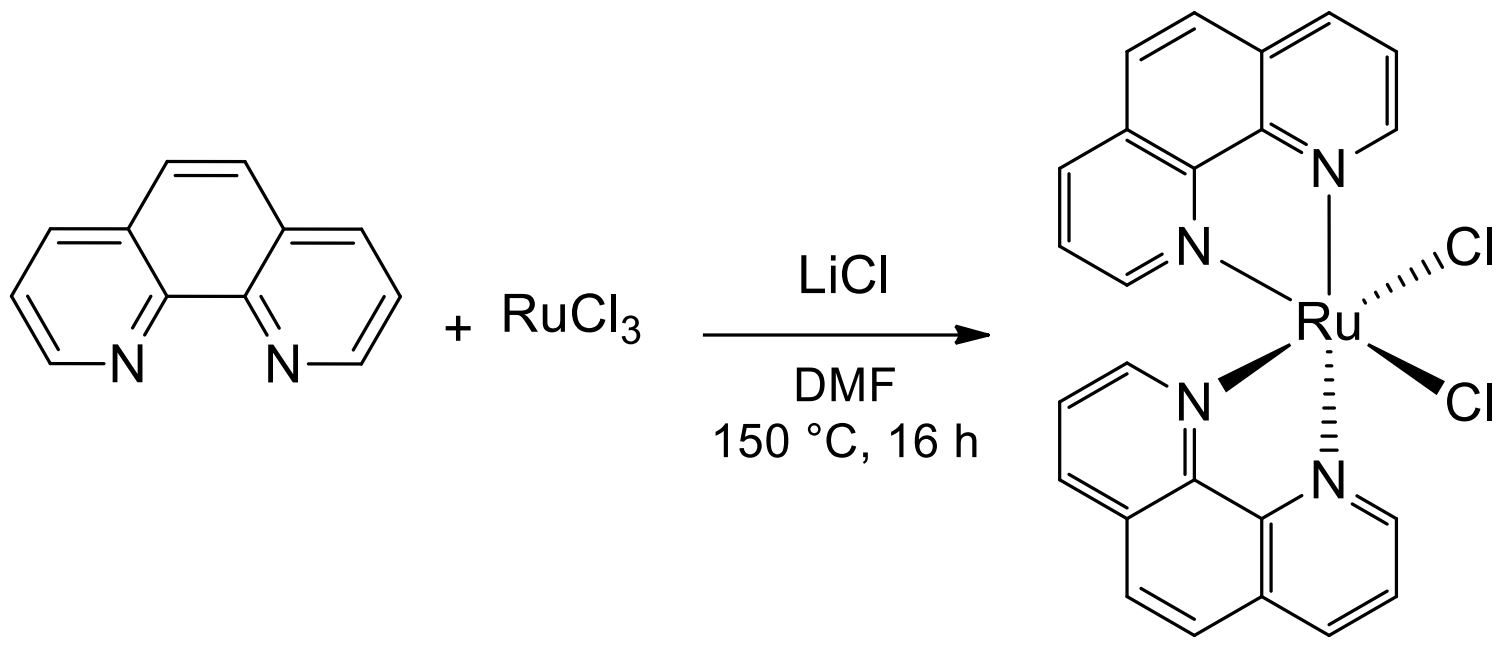

SCHEME S1 | Synthesis of **1**.

$RuCl_3 \cdot 3\,H_2O$ (1 mmol, 261 mg, 1 eq.) and 1,10-phenanthroline monohydrate (397 mg, 2 eq.) were combined, and a solution of LiCl (1.0 g) in 12 ml of dimethylformamide (DMF) was added to the mixture. The reaction was heated under reflux and then allowed to cool to room temperature (RT). The mixture was pipetted into 70 ml of water and centrifuged. The supernatant was decanted off, and the resulting dark solid was washed with 30 ml of water and centrifuged again. This washing and centrifugation step was repeated once more. The product was dried under vacuum at 60 °C, yielding a dark-violet powder. Yield: 245 mg (0.43 mmol), 43%. ^{1}H NMR (600 MHz, DMSO-d_6), δ (ppm) = 10.27 (dd, *J* = 5.2, 1.0 Hz, 2H), 8.71 (dd, *J* = 8.2, 1.0 Hz, 2H), 8.29 (d, *J* = 8.9 Hz, 2H), 8.24 (dd, *J* = 7.7, 0.6 Hz, 2H), 8.21 (dd, *J* = 8.2, 5.2 Hz, 2H), 8.13 (d, *J* = 8.9 Hz, 2H), 7.75 (dd, *J* = 5.4, 0.6 Hz, 2H), 7.33 (dd, *J* = 8.0, 5.5 Hz, 2H).

Synthesis of 2-((1,10-phenanthrolin-5-yl)thio)ethan-1-amine (**3**):

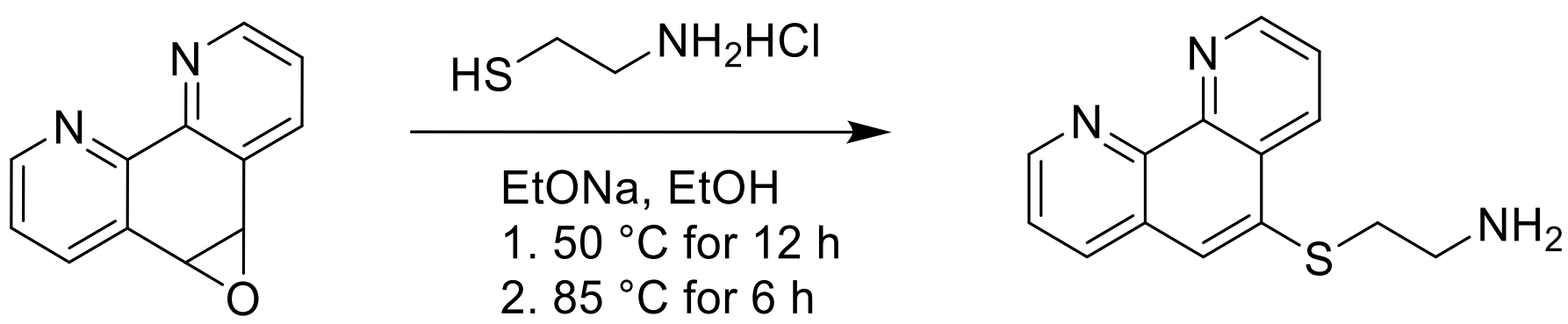

SCHEME S2 | Synthesis of **3**.

Initially, 130 mg (1.147 mmol, 3 eq.) of 2-aminoethanethiol hydrochloride was dissolved in 27 mL of a 0.1 M sodium ethoxide ethanolic solution. This solution was then added to a separate solution containing 66 mg (0.337 mmol) of Phen-epoxy in 3 mL of dry ethanol, all at room temperature. The resulting reaction mixture was stirred under an argon atmosphere at 50 °C for 15 hours. Subsequently, the mixture was heated to reflux (80 °C, 5 h) to facilitate the removal of water and to regenerate aromaticity. Upon cooling, a white solid precipitated. The solution displayed a pH of 6, and it was neutralized by the addition of a few drops of a saturated sodium bicarbonate solution. This neutralization led to more precipitation of the white solid material. The resulting solid was isolated by filtration, and the filtrate (solvent) was then evaporated. To the residue of the solid obtained from the filtrate, 20 ml chloroform was added, and it was washed three times with 50 ml water. The organic phase was dried over sodium sulphate, filtered and the solvent was evaporated to yield a yellow viscous oil. After 48 h, some white material was accumulated, and this is probably remaining salts which will not affect the next step, that's why a further purification was unnecessary. Yield: 81 mg, 93%. ^{1}H NMR (500 MHz, $CDCl_3$), δ ppm: δ 9.21 (dd, *J* = 4.3, 1.6 Hz, 1H), 9.14 (dd, *J* = 4.3, 1.7 Hz, 1H), 8.82 (dd,

J = 8.3, 1.6 Hz, 1H), 8.16 (dd, *J* = 8.1, 1.7 Hz, 1H), 7.84 (s, 1H), 7.70 (dd, *J* = 8.3, 4.3 Hz, 1H), 7.62 (dd, *J* = 8.1, 4.3 Hz, 1H), 3.16 (t, *J* = 6.3 Hz, 2H), 3.00 (t, *J* = 6.3 Hz, 2H).

Synthesis of N^1-(1,10-phenanthrolin-5-yl)ethane-1,2-diamine (**4**):

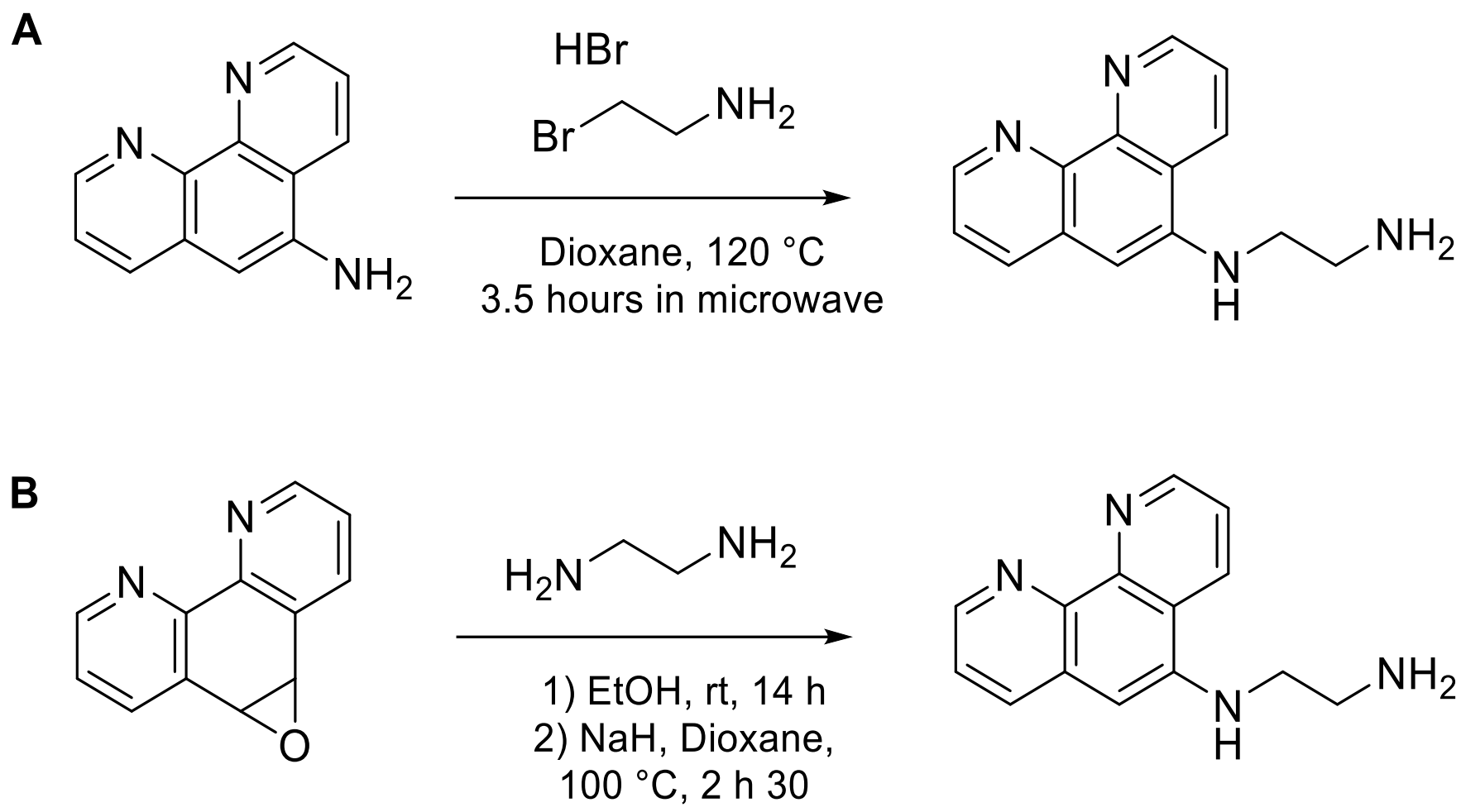

SCHEME S3 | Synthesis of **4**.

Method A: 5-Amino-1,10-phenanthroline (100 mg, 0.512 mmol) and 2-bromoethylamine hydrobromide (114 mg, 0.563 mmol, 1.15 eq.) were dissolved in approximately 10 ml of dioxane. The reactants were stirred at room temperature for 15 minutes under an argon atmosphere. The reaction vessel was sealed and heated in a microwave at 120 °C for 3.5 hours, resulting in the precipitation of a brown solid. The solvent was removed under reduced pressure, and the crude product was added to 4 ml of water. The pH of the solution was adjusted to 10-11 using sodium hydroxide solution (1 M). The solvent was then removed under vacuum, yielding a highly viscous red oil. The crude product was purified using column chromatography on alumina, starting with DCM:MeOH (9:1) to eluate the unreacted 5-amino-1,10-phenanthroline then with DCM:MeOH:NH_2 (13:7:1) to eluate the remaining product. Thin-layer chromatography (TLC) indicated the presence of some unreacted starting material, necessitating a second column chromatography using the same eluents. A total of 100 mg (0.420 mmol, 82%) of the product was isolated.

Method B: 500 mg (2.55 mmol) of 5,6-epoxy-1,10-phenanthroline are dissolved in 40 mL of EtOH, then 6.7 mL (100 mmol, 40 eq.) of ethylenediamine are added. The reaction is stirred at room temperature for 14 h. The solvent and the ethylenediamine are evaporated until obtention of a beige powder, corresponding to the pure alcohol intermediate. This product is then suspended in 20 mL of 1,4-dioxane, and 250 mg (10.4 mmol) of NaH are added. The suspension is heated at 100 °C for 2 h. 50 mg (2.1 mmol) of supplementary NaH are added, and the reaction is maintained 30 more minutes. The reaction medium is cooled to 40 °C, 25 mL of MeOH are added under stirring to dissolve the oily product, and the solvents are evaporated. The crude is dissolved in a minimum of CH_2Cl_2/MeOH 9:1 and purified on a short column chromatography (CH_2Cl_2/MeOH 9:1 to eliminate the impurities, then CH_2Cl_2/MeOH 1:1 to eluate the product). The oily product is dissolved in 35 mL of water, and then lyophilized to give 482 mg (2.02 mmol, 79%) of hygroscopic yellow powder. The product is stored under argon.

Both method yield the same product. ^{1}H NMR (500 MHz, DMSO-d_6), δ ppm: δ 9.02 (dd, *J* = 4.2, 1.4 Hz, 1H), 8.71 (dd, *J* = 8.5, 1.5 Hz, 1H), 8.69 (dd, *J* = 4.2, 1.6 Hz, 1H), 8.06 (dd, *J* = 8.1, 1.6 Hz, 1H), 7.66 (dd, *J* = 8.5, 4.2 Hz, 1H), 7.51 (dd, *J* = 8.1, 4.2 Hz, 1H), 6.70 (s, 1H),

6.65 (t, *J* = 5.2 Hz, 1H, -NH-), 3.50 (dd, *J* = 5.8, 5.2 Hz, 2H, -NH-C$\underline{H}_2$-), 3.17 (t, *J* = 5.8 Hz, 2H, -C$\underline{H}_2$-NH_2).

Synthesis of *tert*-butyl (2-((1,10-phenanthrolin-5-yl)amino)-2-oxoethyl)carbamate (**5**):

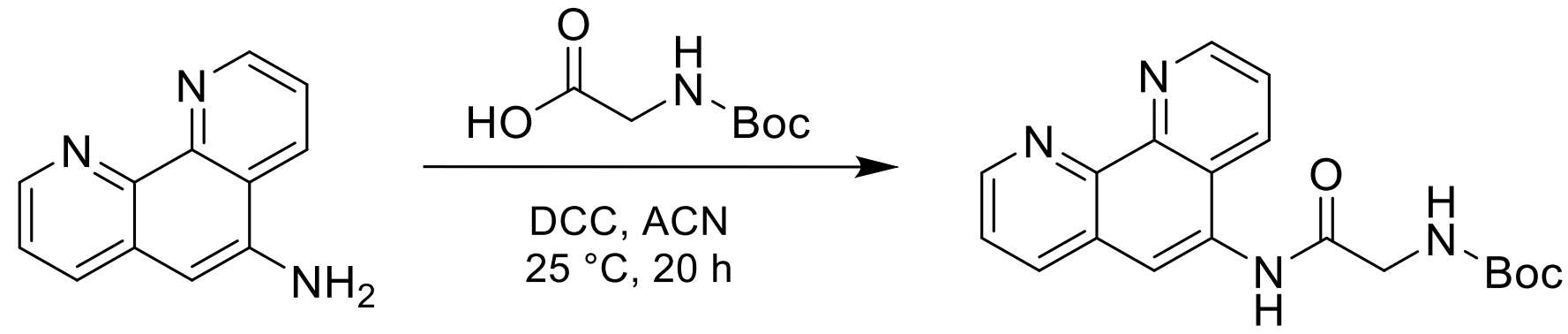

SCHEME S4 | Synthesis of **5**.

A solution of commercial *N*-(*tert*-butoxycarbonyl)glycine (1.31 g, 7.5 mmol) and *N*,*N'*-dicyclohexylcarbodiimide (0.8 g, 3.9 mmol) in acetonitrile (15 ml) was stirred at room temperature for 2 h and filtered to remove the *N*,*N'*-dicyclohexylurea precipitate. The resulting activated acid was used without further purification and added to a suspension of 5-amino-1,10-phenanthroline (150 mg, 0.775 mmol) in 15 ml of acetonitrile. Upon addition of the activated acid, 5-amino-1,10-phenanthroline dissolved completely, and the mixture was stirred at room temperature for 20 h under an argon atmosphere. The solvent was then removed under reduced pressure, the remaining solid was dissolved in dichloromethane and washed 3 times with water, then with a basic solution ($NaHCO_3$), then decanted, dried over $MgSO_4$ and the solvent was evaporated to yield a yellow powder. Yield of the crude product: 85%. ^{1}H NMR (500 MHz, DMSO-d_6), δ ppm: δ 10.16 (s, 1H, Ar-NH-), 9.12 (dd, *J* = 4.2, 1.5 Hz, 1H), 9.04 (dd, *J* = 4.3, 1.7 Hz, 1H), 8.62 (dd, *J* = 8.4, 1.0 Hz, 1H), 8.46 (dd, *J* = 8.1, 1.6 Hz, 1H), 8.13 (s, 1H), 7.80 (dd, *J* = 8.4, 4.2 Hz, 1H), 7.74 (dd, *J* = 8.1, 4.3 Hz, 1H), 7.21 (t, *J* = 5.8 Hz, 1H, -NH-Boc), 3.94 (d, *J* = 6.0 Hz, 2H, -CH_2-), 1.43 (s, 9H, 3x-C$\underline{H}_3$).

Synthesis of complexes **6-9:**

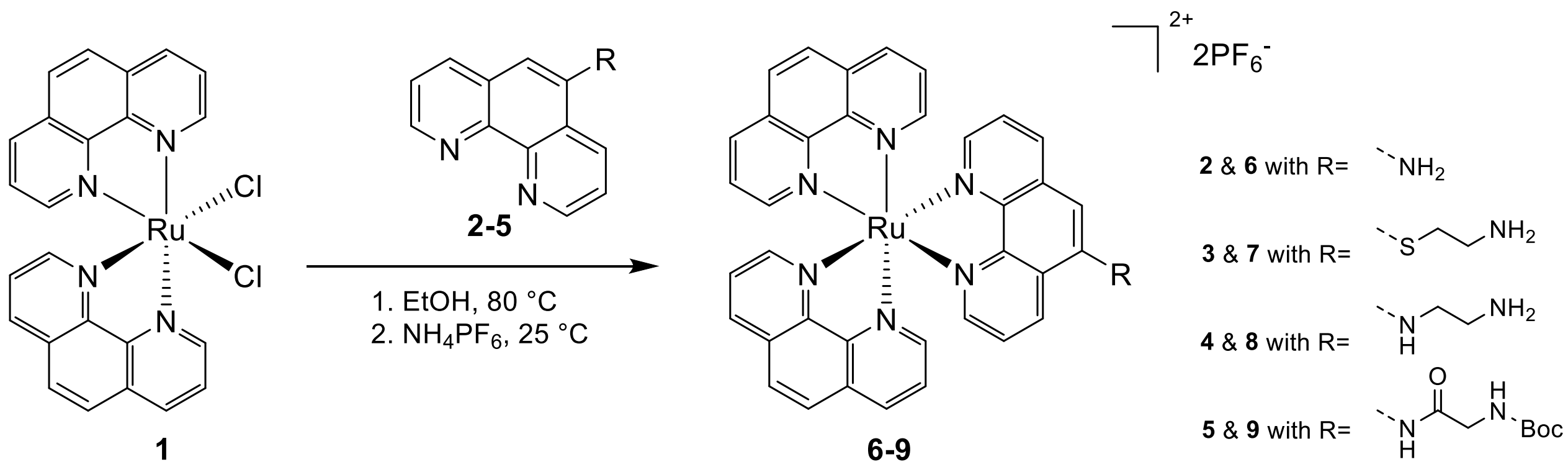

SCHEME S5 | Synthesis of **6**-**9**.

General procedure: In a 25 ml two-necked flask under an argon atmosphere, 1.2 eq. of **2-5** were added to a solution of **1** dissolved in 4-10 ml of ethanol. The mixture was heated at reflux overnight. After cooling to room temperature, a saturated aqueous solution of NH_4PF_6 was added. The coloured precipitate was filtered, washed with ethanol and water, and dried to yield an orange-red powder. Yield: 50-80%.

^{1}H NMR (500 MHz, acetone-d_6), δ ppm of **6**: 8.92 (dd, *J* = 8.6, 1.0 Hz, 1H), 8.79 (dd, *J* = 8.4, 1.3 Hz, 2H), 8.77 (dd, *J* = 8.5, 1.3 Hz, 1H), 8.77 (dd, *J* = 8.4, 1.3 Hz, 1H), 8.42 (dd, *J* = 5.2, 1.2 Hz, 1H), 8.41-8.40 (m, 4H), 8.39 (dd, *J* = 5.2, 1.3 Hz, 2H), 8.35 (dd, *J* = 5.2, 1.2 Hz, 2H),

8.32 (dd, *J* = 8.4, 1.1 Hz, 1H), 7.92 (dd, *J* = 5.2, 1.1 Hz, 1H), 7.85 (dd, *J* = 8.3, 5.3 Hz, 1H), 7.83 (dd, *J* = 8.3, 5.3 Hz, 1H), 7.79 (dd, *J* = 8.2, 5.3 Hz, 1H), 7.78 (dd, *J* = 8.2, 5.3 Hz, 1H), 7.74 (dd, *J* = 8.5, 5.2 Hz, 1H), 7.52 (dd, *J* = 8.4, 5.2 Hz, 1H), 7.30 (s, 1H), 6.38 (br. s, 2H).

^{1}H NMR (500 MHz, acetone-*d*$_6$), δ ppm of **7**: 8.92 (dd, *J* = 8.6, 1.2 Hz, 1H), 8.80-8.77 (m, 4H), 8.67 (dd, *J* = 8.3, 1.2 Hz, 1H), 8.43 (dd, *J* = 5.4, 1.2 Hz, 1H), 8.42 (dd, *J* = 5.3, 1.2 Hz, 1H), 8.41 (dd, *J* = 5.3, 1.2 Hz, 1H), 8.41-8.40 (m, 4H), 8.40 (s, 1H), 8.39 (dd, *J* = 5.3, 1.2 Hz, 1H), 8.37 (dd, *J* = 5.3, 1.2 Hz, 1H), 8.28 (dd, *J* = 5.2, 1.2 Hz, 1H), 7.82 (dd, *J* = 8.6, 5.3 Hz, 1H), 7.82 (dd, *J* = 8.0, 5.2 Hz, 1H), 7.81 (dd, *J* = 8.3, 5.2 Hz, 1H), 7.80 (dd, *J* = 8.3, 5.2 Hz, 1H), 7.79 (dd, *J* = 8.3, 5.2 Hz, 1H), 7.74 (dd, *J* = 8.3, 5.2 Hz, 1H), 3.66 (t, *J* = 6.6 Hz, 2H, -C<u>H</u>$_2$-NH$_2$), 3.58 (dt, *J* = 6.6, 1.0 Hz, 2H, -S-C<u>H</u>$_2$-).

^{1}H NMR (400 MHz, acetone-*d*$_6$,), δ (ppm) of **8**: 8.86 (dd, *J* = 8.6, 1.0 Hz, 1H), 8.82 (dd, *J* = 8.3, 1.2 Hz, 1H), 8.81 (dd, *J* = 8.3, 1.2 Hz, 1H), 8.79 (dd, *J* = 8.3, 1.4 Hz, 1H), 8.78 (dd, *J* = 8.3, 1.4 Hz, 1H), 8.43-8.42 (m, 4H), 8.42 (dd, *J* = 5.3, 1.2 Hz, 2H), 8.41 (dd, *J* = 8.4, 1.2 Hz, 1H), 8.39 (dd, *J* = 8.1, 1.1 Hz, 1H), 8.37 (dd, *J* = 5.3, 1.2 Hz, 1H), 8.37 (dd, *J* = 5.2, 1.1 Hz, 1H), 7.94 (dd, *J* = 5.2, 1.1 Hz, 1H), 7.88 (dd, *J* = 8.2, 5.2 Hz, 1H), 7.85 (dd, *J* = 8.2, 5.2 Hz, 1H), 7.81 (dd, *J* = 8.2, 5.2 Hz, 1H), 7.80 (dd, *J* = 8.2, 5.3 Hz, 1H), 7.73 (dd, *J* = 8.6, 5.3 Hz, 1H), 7.56 (dd, *J* = 8.3, 5.3 Hz, 1H), 7.30 (br. s, 1H), 6.75 (t, *J* = 5.3 Hz, 1H), 4.05-4.01 (m, 2H), 3.92 (q, *J* = 5.3 Hz, 2H).

^{1}H NMR (500 MHz, acetone-*d*$_6$), δ ppm of **9**: 9.92 (s, 1H, Ar-N<u>H</u>-), 8.91 (dd, *J* = 8.5, 0.9 Hz, 1H), 8.80-8.77 (m, 4H), 8.73-8.71 (m, 1H), 8.71 (s, 1H), 8.43 (dd, *J* = 5.2, 1.0 Hz, 1H), 8.42 (dd, *J* = 5.2, 1.2 Hz, 1H), 8.41 (dd, *J* = 5.2, 1.2 Hz, 1H), 8.41 (s, 4H), 8.39 (dd, *J* = 5.2, 1.2 Hz, 1H), 8.38 (dd, *J* = 5.2, 1.2 Hz, 1H), 8.30 (dd, *J* = 5.2, 1.2 Hz, 1H), 7.81 (dd, *J* = 8.3, 5.2 Hz, 1H), 7.81 (dd, *J* = 8.3, 5.2 Hz, 1H), 7.80 (dd, *J* = 8.2, 5.3 Hz, 1H), 7.80 (dd, *J* = 8.4, 5.2 Hz, 1H), 7.80 (dd, *J* = 8.4, 5.2 Hz, 1H), 7.75 (dd, *J* = 8.3, 5.3 Hz, 1H), 6.64 (br. s, 1H, -N<u>H</u>-Boc), 4.11 (d, *J* = 5.8 Hz, 2H, -C<u>H</u>$_2$-), 1.46 (s, 9H, 3x-C<u>H</u>$_3$).

<u>Synthesis of **10-12**:</u>

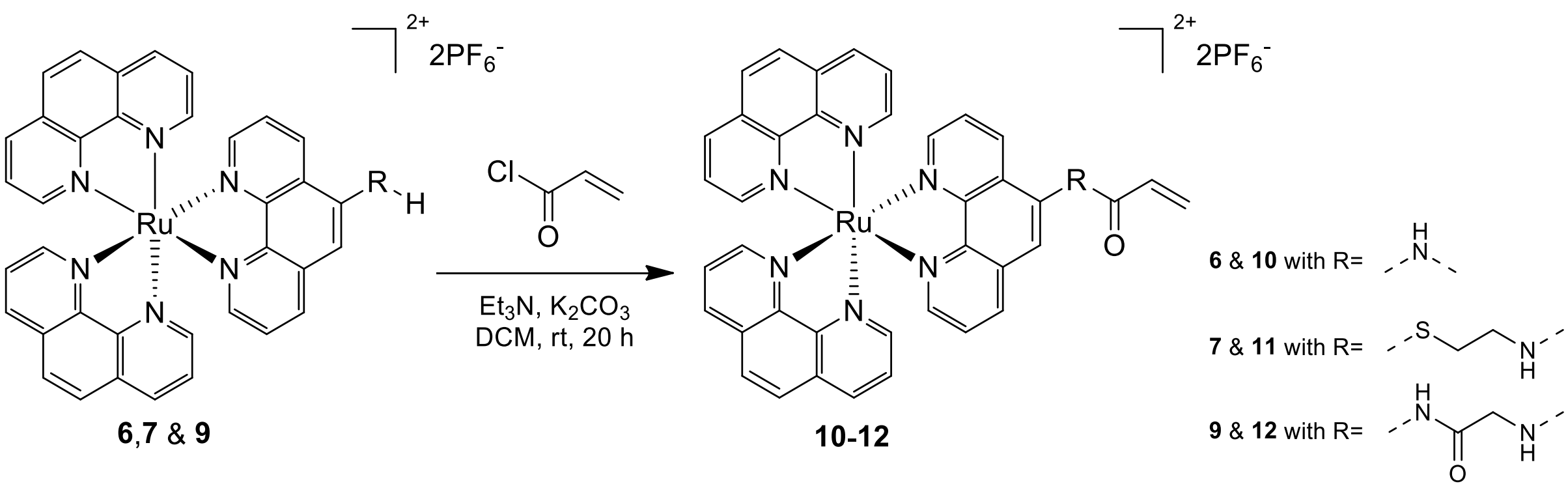

SCHEME S6 | General scheme for the synthesis of **10-12**.

<u>General procedure:</u> 1 equivalent of (**6**, **7** or **9**) was dissolved in 10 mL of dichloromethane. In case of **9**, the crude product was dissolved in DCM. The mixture was stirred under HCl gas at room temperature for 2 days, with occasional addition of more HCl gas. After two days, a dark red solid formed at the bottom of the flask. The DCM was decanted, and the flask was washed once more with DCM. The product was used without further purification.

For all compounds (**6**, **7** or HCl-treated **9**), triethylamine (2 eq.) addition was followed by a gradual dropwise addition of acryloyl chloride (1.5 eq.) diluted in DCM. After 3 h stirring at room temperature, few drops of acetone were added to enhance solubility. Potassium carbonate (2 eq.) was added as an inorganic base. After 20 h, the solvents were evaporated,

and the residue was dissolved in DCM. The organic phase was washed three times with water, dried over $MgSO_4$, filtered, and evaporated to obtain the product as a brown or red powder. Yields: 50-90 %.

^{1}H NMR (500 MHz, acetone-*d*$_6$), δ ppm of **10**: 9.96 (s, 1H, -NH-), 8.99 (dd, *J* = 8.6, 1.1 Hz, 1H), 8.87 (s, 1H), 8.80-8.77 (m, 4H), 8.72 (dd, *J* = 8.3, 1.2 Hz, 1H), 8.44 (dd, *J* = 5.3, 1.0 Hz, 1H), 8.43 (dd, *J* = 5.3, 1.3 Hz, 1H), 8.41 (dd, *J* = 5.1, 1.3 Hz, 1H), 8.41 (s, 4H), 8.39 (dd, *J* = 5.3, 1.3 Hz, 1H), 8.38 (dd, *J* = 5.2, 1.3 Hz, 1H), 8.30 (dd, *J* = 5.3, 1.3 Hz, 1H), 7.82 (dd, *J* = 8.3, 5.3 Hz, 1H), 7.80 (dd, *J* = 8.3, 5.3 Hz, 1H), 7.80 (dd, *J* = 8.3, 5.2 Hz, 1H), 7.80 (dd, *J* = 8.3, 5.3 Hz, 1H), 7.75 (dd, *J* = 8.3, 5.3 Hz, 1H), 7.75 (dd, *J* = 6.3, 3.3 Hz, 0.5H), 7.66 (dd, *J* = 6.3, 3.3 Hz, 0.5H), 6.78 (dd, *J* = 16.9, 10.2 Hz, 1H, -CH=), 6.51 (dd, *J* = 16.9, 1.8 Hz, 1H, =CH_2), 5.90 (dd, *J* = 10.2, 1.8 Hz, 1H).

^{1}H-NMR (500 MHz, acetone-*d*$_6$), δ ppm of **11**: 8.87 (dd, *J* = 8.6 Hz, 1.2 Hz, 1H), 8.79 (m, 4H), 8.65 (dd, *J* = 8.4, 1.2 Hz, 1H), 8.64 (s, 1H), 8.43 (dd, *J* = 5.2, 1.2 Hz, 1H), 8.43 (dd, *J* = 5.2, 1.2 Hz, 1H), 8.43 (dd, *J* = 5.2, 1.2 Hz, 1H), 8.41 (m, 4H), 8.39 (dd, *J* = 5.2, 1.2 Hz, 1H), 8.38 (dd, *J* = 5.2, 1.2 Hz, 1H), 8.30 (dd, *J* = 5.3, 1.2 Hz, 1H), 7.82 (dd, *J* =8.4, 5.3 Hz, 1H), 7.82 (dd, *J* = 8.5, 5.3 Hz, 1H), 7.81 (dd, *J* = 8.1, 5.2 Hz, 1H), 7.80 (dd, *J* = 8.6, 5.2 Hz, 1H), 7.80 (dd, *J* = 8.3, 5.2 Hz, 1H), 7.76 (dd, *J* = 8.3, 5.2 Hz, 1H), 7.76 (br. t, *J* = 7.4 Hz, 1H, -NH-), 6.26 (dd, *J* = 17.0, 9.6 Hz, 1H, -CH=), 6.20 (dd, *J* = 17.0, 2.6 Hz, 1H, =CH_2), 5.58 (dd, *J* = 9.6, 2.6 Hz, 1H, =CH_2), 3.68 (m, 2H), 3.52 (t, *J* = 7.0 ppm, 2H).

^{1}H-NMR (500 MHz, acetone-*d*$_6$), δ ppm of **12**: 10.23 (s, 1H, -NH-Ar), 8.97 (d, *J* = 8.4 Hz, 1H), 8.78 (dd, *J* = 8.3, 1.2 Hz, 4H), 8.73 (s, 1H), 8.68 (d, *J* = 8.4 Hz, 1H), 8.43-8.40 (m, 3H), 8.40 (s, 4H), 8.39 (dd, *J* = 5.3, 1.2 Hz, 1H), 8.37 (dd, *J* = 5.3, 1.2 Hz, 1H), 8.28 (dd, *J* = 5.3, 1.0 Hz, 1H), 8.14 (t, *J* = 5.0 Hz, 1H, NH-CO), 7.83-7.78 (m, 5H), 7.73 (dd, *J* = 8.3, 5.3 Hz, 1H), 6.44 (dd, *J* = 17.0, 10.2 Hz, 1H, -CH=), 6.31 (dd, *J* = 17.0, 2.0 Hz, 1H, =CH_2), 5.70 (dd, *J* = 10.2, 2.0 Hz, 1H, =CH_2), 4.31 (t, *J* = 5.0 Hz, 2H, -CH_2NH-).

Synthesis of copolymer **PFPA-*co*-Surf-EH3**:

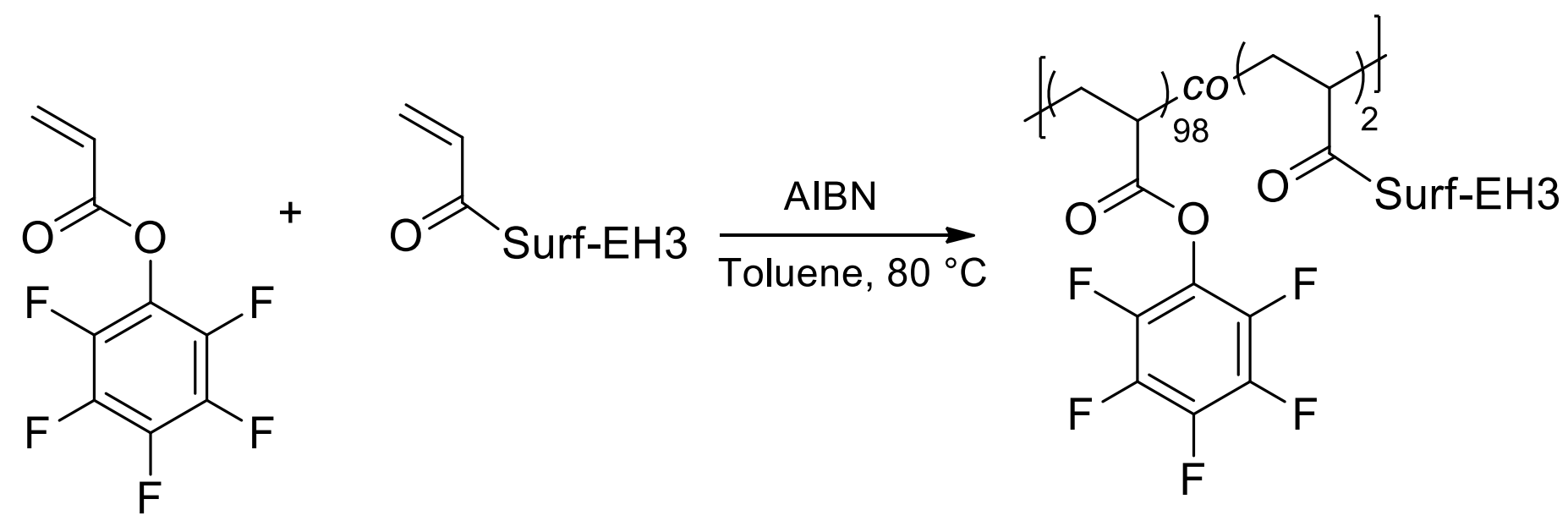

SCHEME S7 | Copolymer PFPA-*co*-Surf-EH3.

2.9 mL (17.6 mmol) of pentafuorophenyl acrylate, 200 mg (0.36 mmol) of Surf-EH3A and 30 mg (183 µmol) of azobisisobutyronitrile were dissolved in 30 mL of toluene. The solution was then purged with argon for 10 minutes and inserted into a preheated (80 °C) oil bath. The polymerisation was terminated after 48 hours by purging with air and cooling to room temperature (20 °C). The polymer was first precipitated once in *n*-heptane, redissolved in THF and reprecipitated in *n*-heptane. After this a liquid-solid extraction in *n*-heptane was performed and after centrifugation in *n*-heptane the residual solvent was removed under vacuum resulting in a yield of 3.7 g (85 %). ^{1}H NMR (500 MHz, $CDCl_3$), δ ppm: δ 3.69 – 3.24 (m, surfactant), 3.08 (br s, 1H of the backbone), 2.56 – 2.04 (br, m, 2H of the backbone), 1.13 (m, surfactant), 0.92 – 0.78 (m, surfactant). GPC: *Mn*: 20 kDa, *Đ*: 1.5.

Synthesis of **13:**

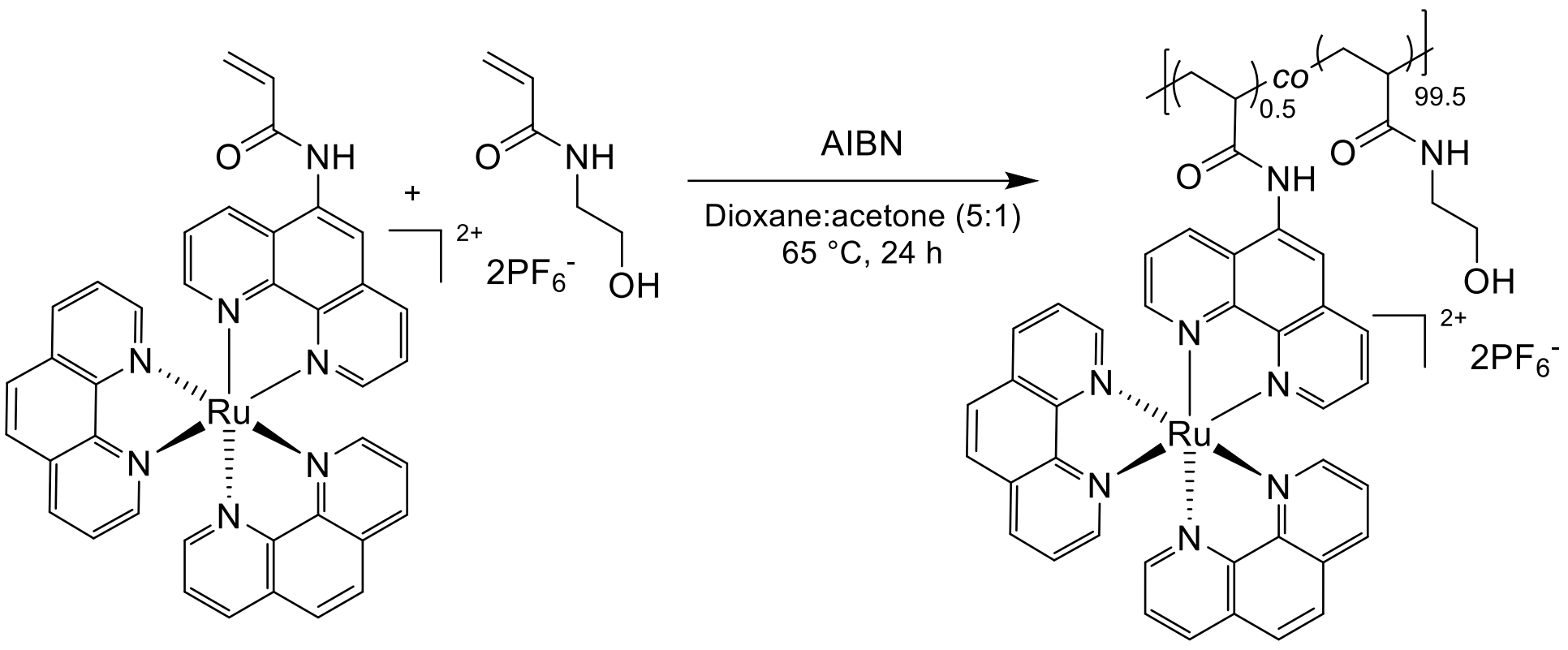

SCHEME S8 | Synthesis of **13**.

50 mg (0.06 mmol) of complex **10** and 181 mg (1.57 mmol) of *N*-hydroxyethyl acrylamide were dissolved in 6 mL of a mixture of dioxane and acetone (5/1). The polymerization mixture was degassed by argon purge before azobisisobutyronitrile (2 mg, 12 µmol) was added under argon stream. The polymerization was started in a preheated oil bath at 65 °C under reflux. To avoid the precipitation of polyHEAAm during the reaction, 0.5 ml of DMSO was added. Nevertheless, the polymer partially precipitated but it was carried on and quenched after 24 hours by air under ice cooling. Most of the solvents were evaporated and 1 ml of DMSO was added to dissolve the precipitated polymer. The polymer was then precipitated in ethyl acetate, isolated, dissolved in water and dialyzed against distilled water in 2 kDa bags for three days. The clear solution was freeze-dried to obtain 77 mg (40%) of a solid yellow polymer. ^{1}H NMR (500 MHz, D_2O), δ ppm: δ 8.78 – 7.43 (br, m, aromatic protons of [Ru]), 3.90 – 3.01 (br, 4H, m, -CH_2CH_2- of HEAAm), 2.35 – 1.25 (br, 3H, m, backbone).

Synthesis of **14**:

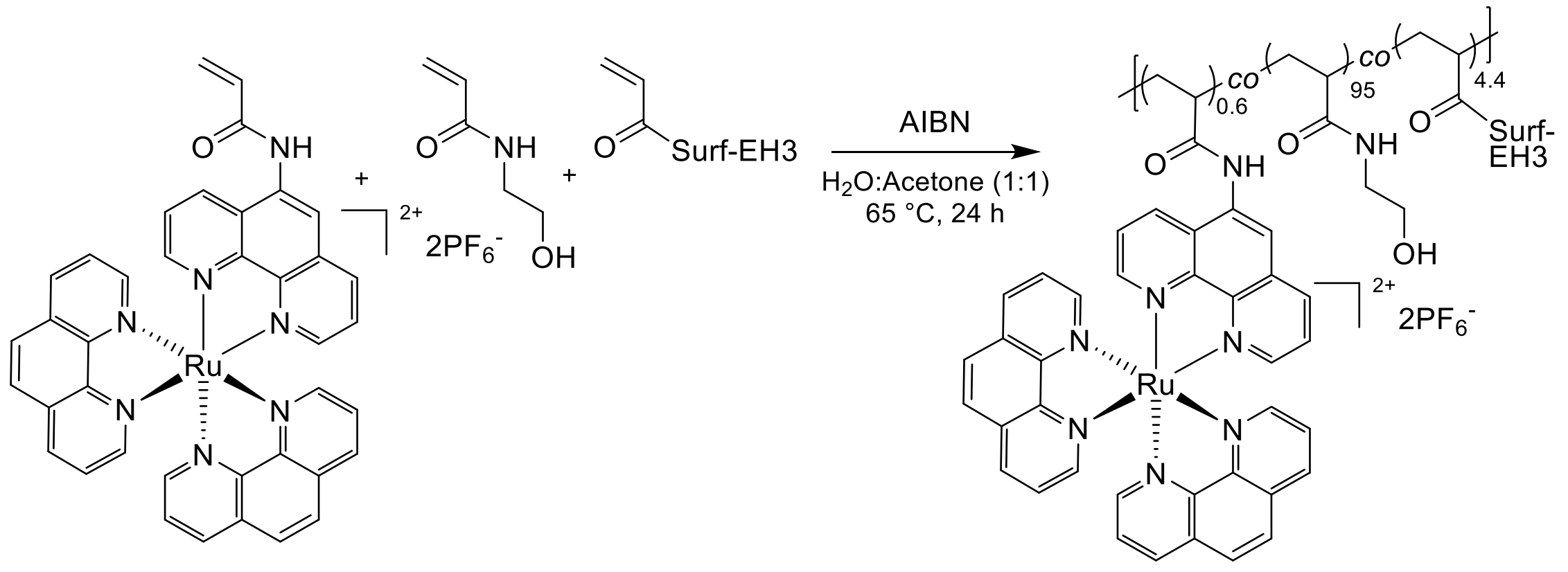

SCHEME S9 | Synthesis of **14**.

33 mg (0.038 mmol) of complex **10** 137 mg (1.19 mmol) of *N*-hydroxyethyl acrylamide, and 18 mg (0.033 mmol) of Surf-EH3A were dissolved in 4 mL of a mixture of water and acetone (1/1). The polymerization mixture was degassed by argon purge, before azobisisobutyronitrile (2 mg, 12 µmol) was added under argon stream. The polymerization was started in a preheated oil bath at 65 °C under reflux. Since polyHEAAm is insoluble in acetone, it started precipitating during the reaction. Nevertheless, the polymerization was carried on and quenched after 24 hours by air under ice cooling. The solvents were evaporated, and diethyl ether was added to extract the unreacted monomers. After evaporation, the polymer was dissolved in water and dialyzed against distilled water in 2 kDa bags for three days. The

solution was turbid and not completely soluble. The sample was freeze-dried to obtain 78 mg (43%) of a solid yellow polymer. ^{1}H NMR (400 MHz, DMSO-d_6), δ ppm: 8.87 – 7.34 (br, m, aromatic protons of [Ru] and -NH- of HEAAm), 5.13 – 4.70 (br, m, -OH of HEAAm), 3.55 – 2.9 (br, m, -CH_2CH_2- of HEAAm), 2.21 – 0.68 (br, m, backbone and surfactant).

Synthesis of **15**:

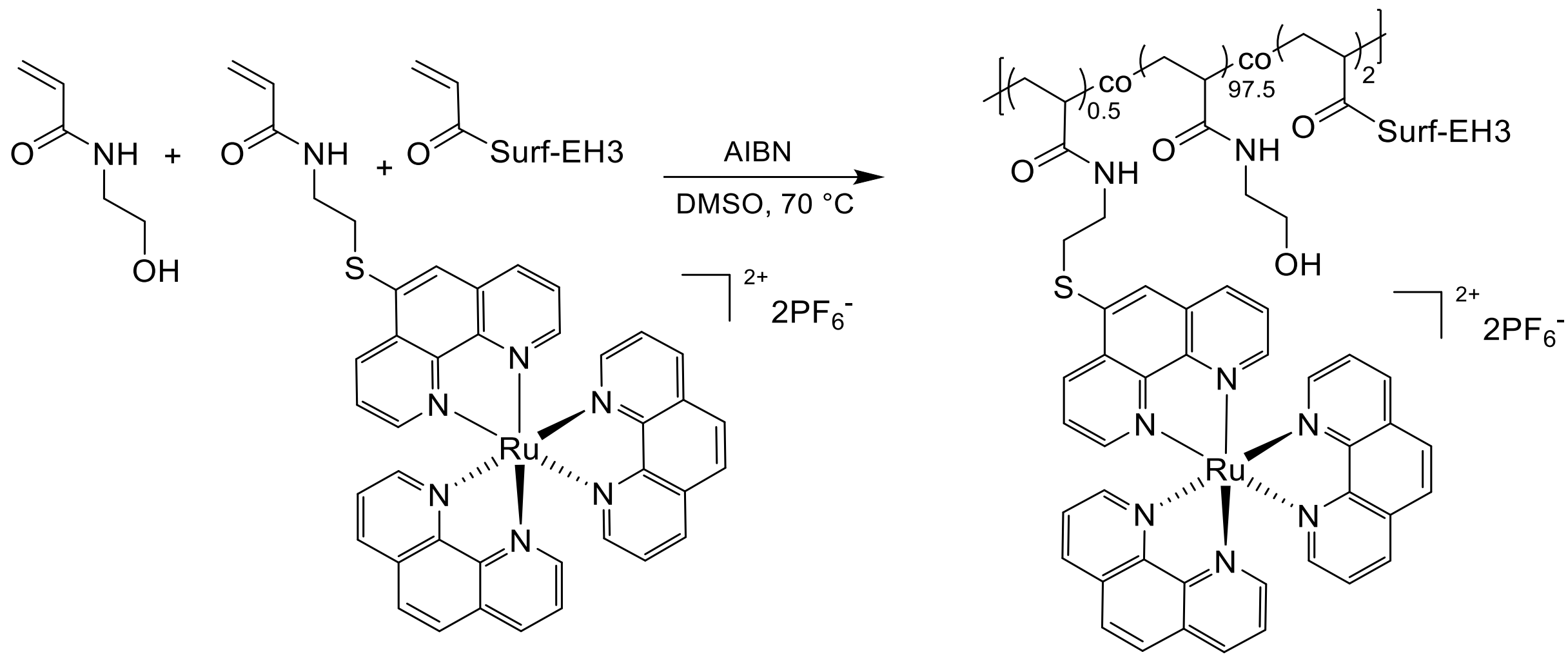

SCHEME S10 | Synthesis of **15**.

144 mg (1.25 mmol) of *N*-hydroxyethyl acrylamide, 14 mg (0.026 mmol, 2 mol%) of Surf-EH3A, 28 mg (0.026 mmol, 2 mol%) of complex **11**, and 2.1 mg (0.013 mmol, 1 mol%) of azobisisobutyronitrile were dissolved in 1 mL of DMSO to give a total volume with the DMSO from the stock solutions of around 2.2 mL. To remove any oxygen, three freeze-pump-thaw cycles were carried out. The solution was heated to 70 °C overnight. Afterwards the polymer was precipitated in ice cold EtOAc (220 mL) and centrifuged. The polymer was redissolved in water and dialyzed against distilled water in 1 kDa bags for two days and afterwards freeze-dried to yield 122 mg (65%) of an orange polymer. ^{1}H NMR (500 MHz, DMSO-d_6), δ ppm: δ 8.89 – 8.31 (m, aromatic protons), 7.82 – 7.30 (m, amide protons of HEAAm), 4.90 (m, hydroxyl protons of HEAAm), 3.55 – 2.83 (m, ethyl protons of HEAAm), 2.09 – 1.24 (m, backbone), 1.11 – 0.72 (m, surfactant).

Synthesis of **16**:

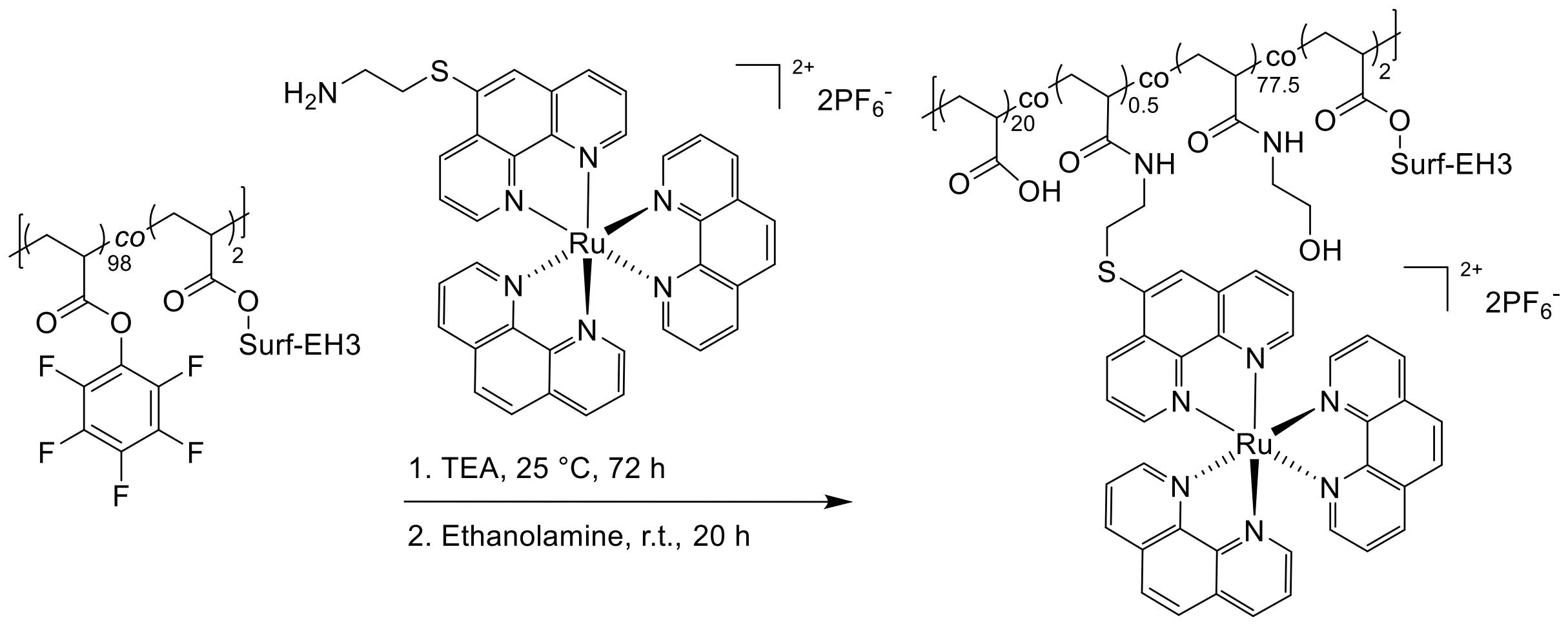

SCHEME S11 | PPM reaction of **16**.

To prepare the reaction mixture, 30 mg (0.029 mmol) of **7** was dissolved in 1.5 mL of *N,N*-dimethylformamide (DMF). In a separate flask, 134 mg (0.56 mmol) of pPFPA-*co*-Surf-EH3 was also dissolved in 2 mL of DMF. Subsequently, the first solution was combined with the second one, and 70 mg (0.67 mmol) of triethylamine was introduced into the resulting mixture. The reaction mixture was then stirred at room temperature for 3 days. Following this initial period, 102 mg of ethanol amine (1.68 mmol, 3 eq.) was added to the mixture, which was then stirred for an additional 20 hours. The solvent was subsequently removed, and the remaining substance was dissolved in water. This solution was dialyzed against distilled water using 2 kDa molecular weight cutoff dialysis bags for 2 days. After the dialysis, the solution was freeze-dried to obtain 72 mg of the final product. ^{1}H NMR (500 MHz, DMSO-d_6), δ ppm: δ 8.84 – 7.46 (br, m, aromatic protons of [Ru]), 3.67 – 2.64 (br, m, -CH2CH2- of HEAAm), 2.3 – 0.7 (br, m, backbone & surfactant).

Synthesis of **17**:

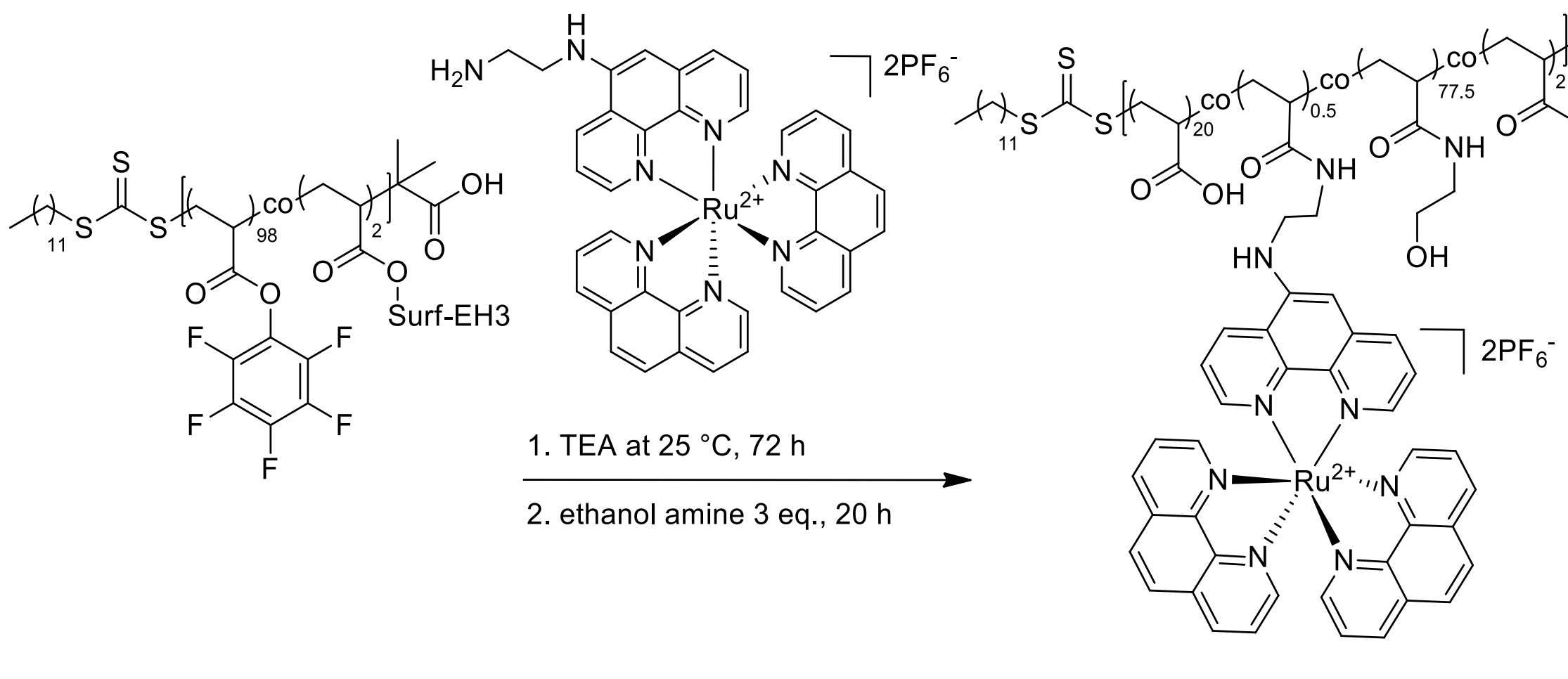

SCHEME S12 | Synthesis of **17**.

To prepare the reaction mixture, 25 mg of **8** (0.04 mmol) was dissolved in 1.5 mL of *N,N*-dimethylformamide (DMF). In a separate flask, 102 mg of pPFPA-*co*-SurfEH3A (0.42 mmol) and 52 mg DMAP (1 mol eq.) were dissolved in 2 mL of DMF. Subsequently, the first solution was combined with the second one, and 70 mg of triethylamine (TEA) (0.67 mmol) was introduced into the resulting mixture. The reaction mixture was then stirred at 40 °C for a duration of 2 days. Following this initial period, 0.075 ml of ethanol amine (3 mol eq.) was added to the mixture, which was then stirred for an additional 24 hours at room temperature.

The solvent was subsequently removed, and the remaining substance was dissolved in water. This solution was dialyzed using 2 kDa molar mass cutoff dialysis bags for a period of 2 days. After the dialysis, the solution was freeze-dried to obtain the final product. Yield: 39 mg. ^{1}H-NMR (500 MHz, DMSO-d_6), δ ppm: δ 8.84 – 7.6 (br, m, aromatic protons of [Ru]), 3.9 – 3.11 (br, m, -CH_2CH_2- of HEAAm overlapped with water peak at, ~3.3), 2.3 – 0.7 (br, m, backbone & surfactant).

Synthesis of **18**:

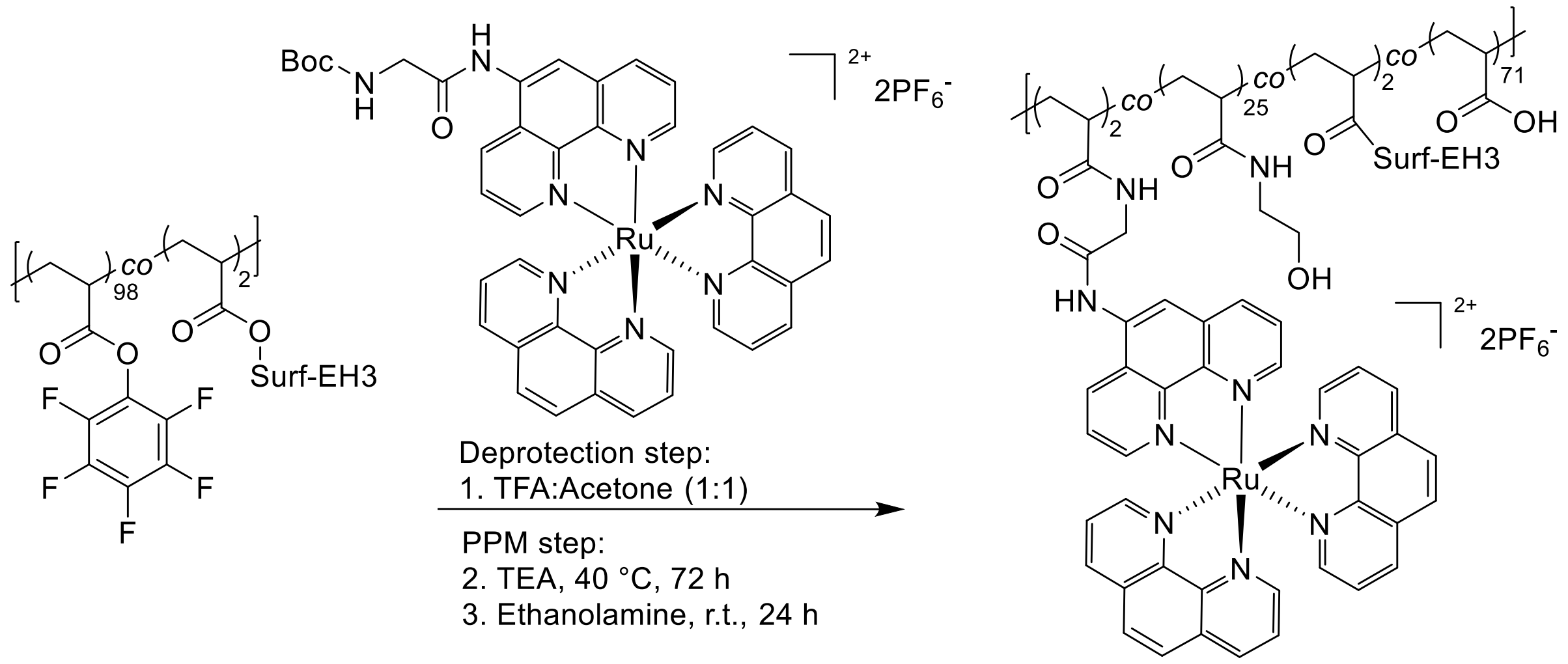

SCHEME S13 | Deprotection of Boc-group and subsequent PPM reaction of **18**.

In a 25 ml round-bottom flask, 54 mg (0.04 mmol) of compound **9** was dissolved in 2 ml of acetone. To this solution, 2 ml of trifluoroacetic acid was added, and the mixture was stirred at room temperature overnight under a nitrogen atmosphere. After this period, the solvent was removed, and the product was used directly without further purification for the next step. Next, 45 mg (0.045 mmol) of the product obtained from the previous step was dissolved in 2 ml of *N*,*N*-dimethylformamide (DMF). In a separate flask, 200 mg of pPFPA-*co*-Surf-EH3 (0.85 mmol) was also dissolved in 2 ml of DMF. The solution containing the product was then combined with the solution of pPFPA-*co*-Surf-EH3, and 130 mg of triethylamine (TEA) (0.67 mmol) was introduced into the resulting mixture. The reaction mixture was stirred at 40 °C for a duration of 3 days. During this time, the solution became turbid, and some materials appeared to be insoluble, in contrast to the initial clear red solution. Following this initial reaction period, 155 mg of ethanol amine (2.55 mmol, 3 equivalents) was added to the mixture at room temperature, and stirring was continued for an additional 24 hours. After the reaction was complete, the polymer was precipitated in acetone and centrifuged to yield an orange solid substance. This solid was then dissolved in water and dialyzed against distilled water using 2 kDa molecular weight cutoff dialysis bags for a period of 3 days. Finally, the dialyzed solution was freeze-dried to obtain 70 mg of a fluffy orange solid. ^{1}H NMR (500 MHz, D_2O), δ ppm: 8.79 – 7.32 (br, m, aromatic protons of [Ru]), 3.79 – 3.24 (br, m, -CH_2CH_2- of ethanol amine), 2.61 – 0.91 (br, m, backbone and surfactant). ^{19}F-NMR (500 MHz, D_2O), δ ppm: No signals.

Synthesis of **19**:

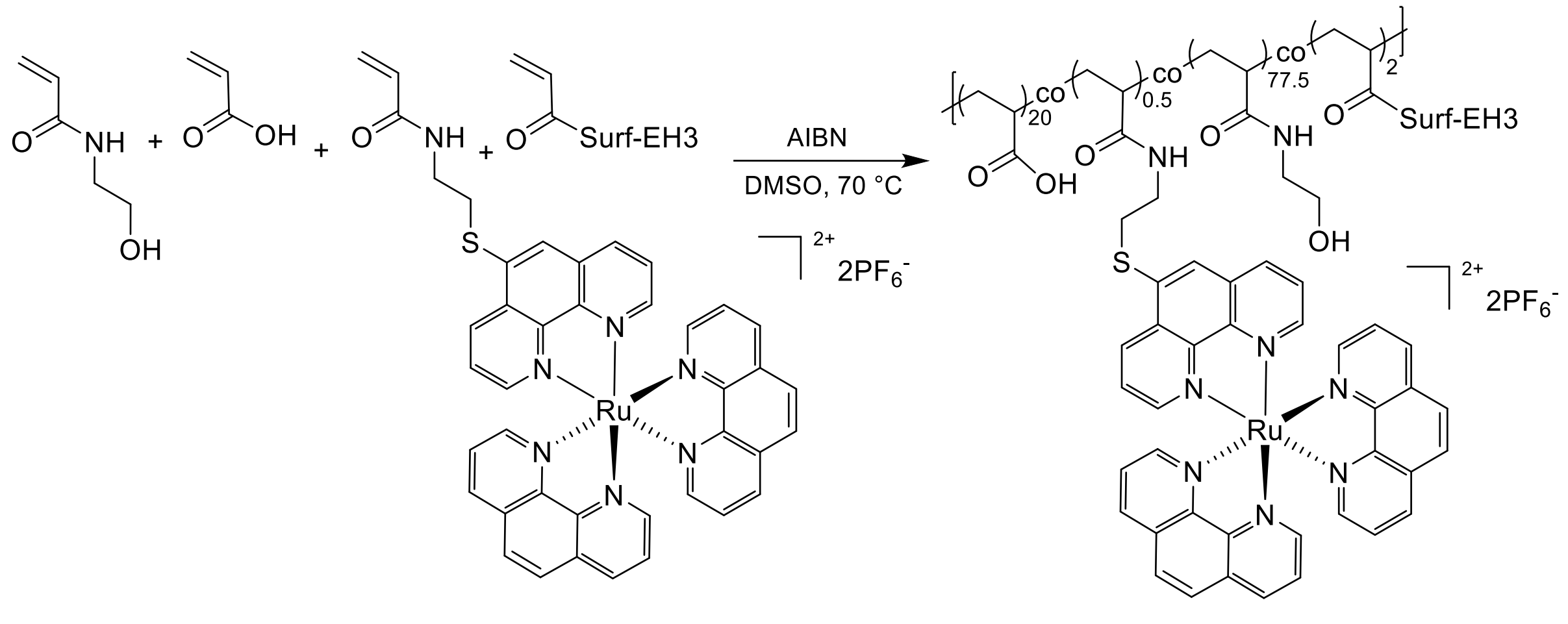

SCHEME S14 | Synthesis of **19**.

76 mg (0.66 mmol) of *N*-hydroxyethyl acrylamide, 12.7 mg (0.18 mmol, 20 mol%) of acrylic acid, 9.3 mg (0.017 mmol, 2 mol%) of Surf-EH3A, 18.7 mg (0.017 mmol, 2 mol%) of complex **11**, and 1.4 mg (0.009 mmol, 1 mol%) of azobisisobutyronitrile were dissolved in 0.3 mL of DMSO to give a total volume with the DMSO from the stock solutions of around 1 mL. To remove any oxygen three freeze-pump-thaw cycles were carried out. The solution was heated to 70 °C for one day. Afterwards the polymer was precipitated in ice cold EtOAc (220 mL) and centrifuged. The polymer was redissolved in water (2-3 mL), dialyzed against distilled water in 1 kDa bags for 2 days, and freeze-dried to yield 117 mg (99%) of an orange polymer. ^{1}H-NMR (500 MHz, DMSO-d_6), δ ppm: δ 8.84 – 8.25 (m, aromatic protons), 7.69 (b, amide protons of HEAAm), 4.24 – 3.99 (m, hydroxyl protons of HEAAm), 3.65 – 2.84 (m, ethyl protons of HEAAm), 2.27 – 0.76 (m, backbone & surfactant).

<u>Unsuccessful synthesis of PPM, path 2:</u>

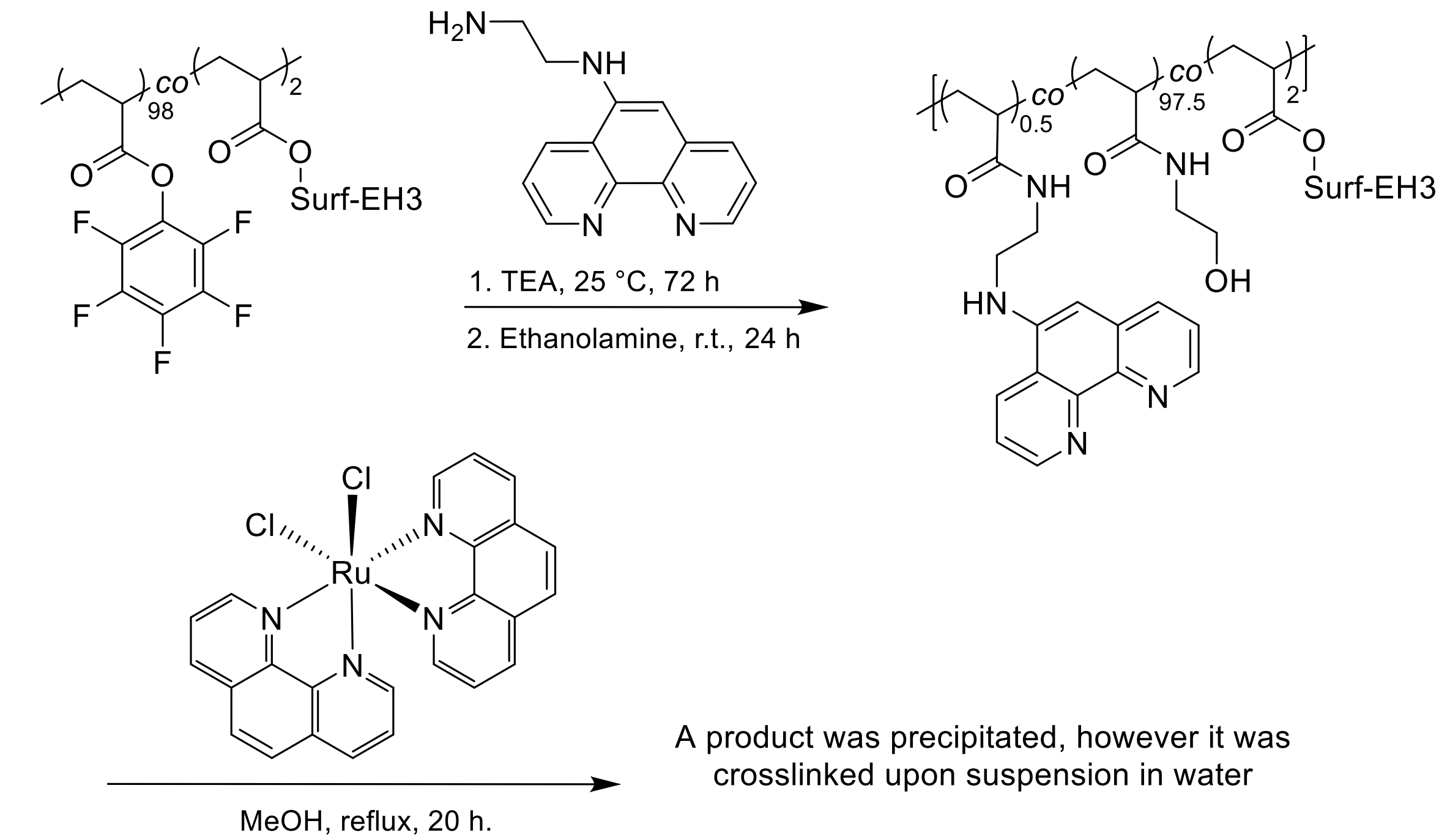

SCHEME S15 | Unsuccessful PPM path 2.

General procedure: PFPA-*co*-SurfEH3 was dissolved in THF (2 mL). N-(1,10-phenanthrolin-5-yl)ethane-1,2-diamine (4) (7 mol%, 0.029 mmol) was dissolved in MeOH (~ 1.5 mL), and 4-

(dimethylamino)pyridine (1 mol eq.) was added. The mixture was stirred at 35 °C for 24 h. It was then cooled to room temperature (~ 20 °C) before monoethanolamine (0.1 mL, 2 mol eq.) was added. After a further 24 hours, the solvent was removed under reduced pressure. The polymer was redissolved in a few mL of water, dialyzed in 1 kDa bags for 48 hours, and freeze-dried. For the second step, the obtained polymer was dissolved in methanol. To this solution, **1** (5 mol eq. relative to the ligand content in the polymer) was added, and the reaction mixture was refluxed overnight. Afterwards, the solvent was removed under reduced pressure. The polymer was redissolved in a few mL of water, dialyzed for 48 hours, and freeze-dried. After this process, the polymer became insoluble in both organic and aqueous solvents, suggesting possible crosslinking. However, this is an assumption that requires confirmation. The reaction was repeated 4 times, consistently yielding the same outcome.

S3 | Supplementary NMR Spectra

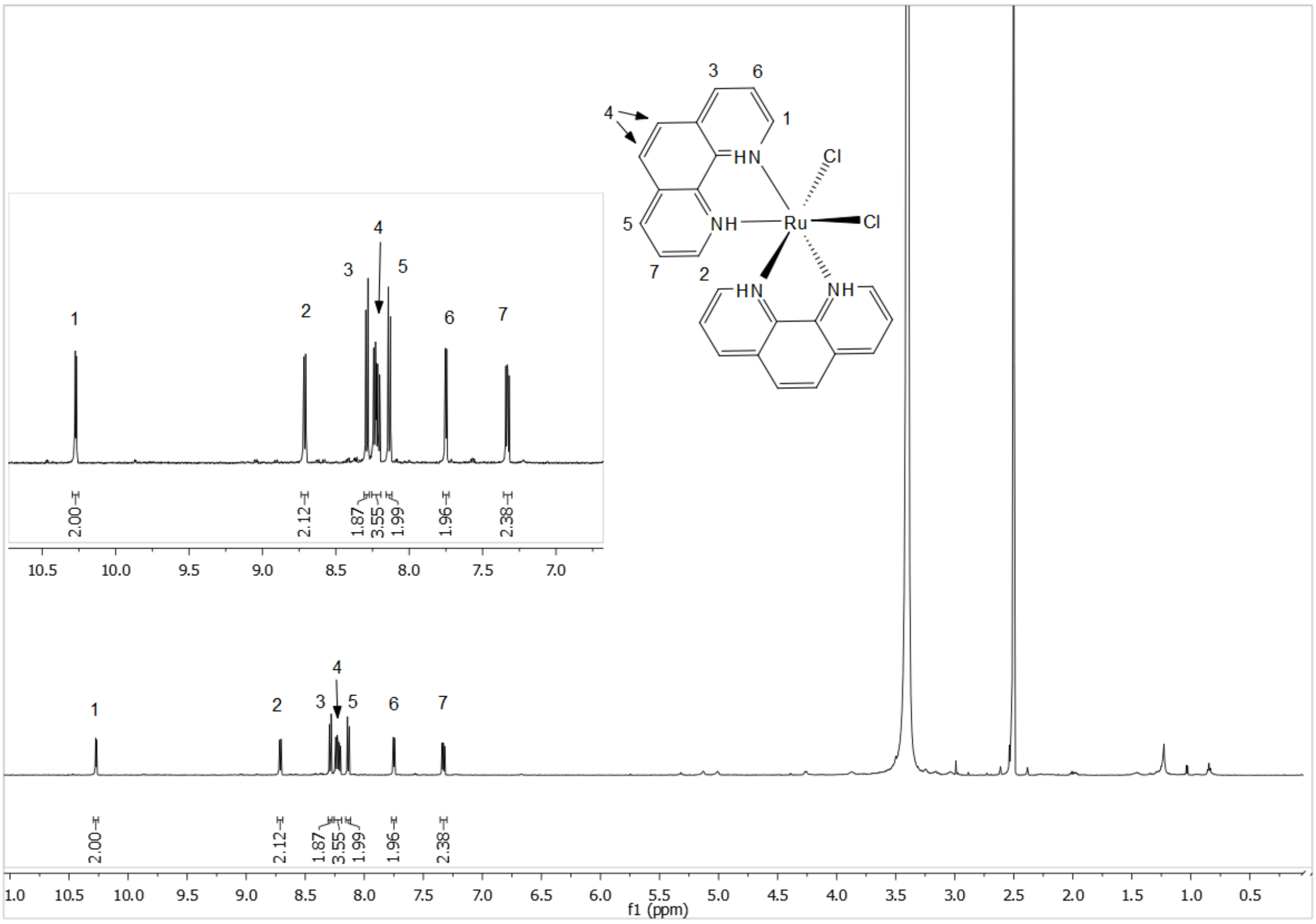

NMR SPECTRUM S116 | Compound **1**.

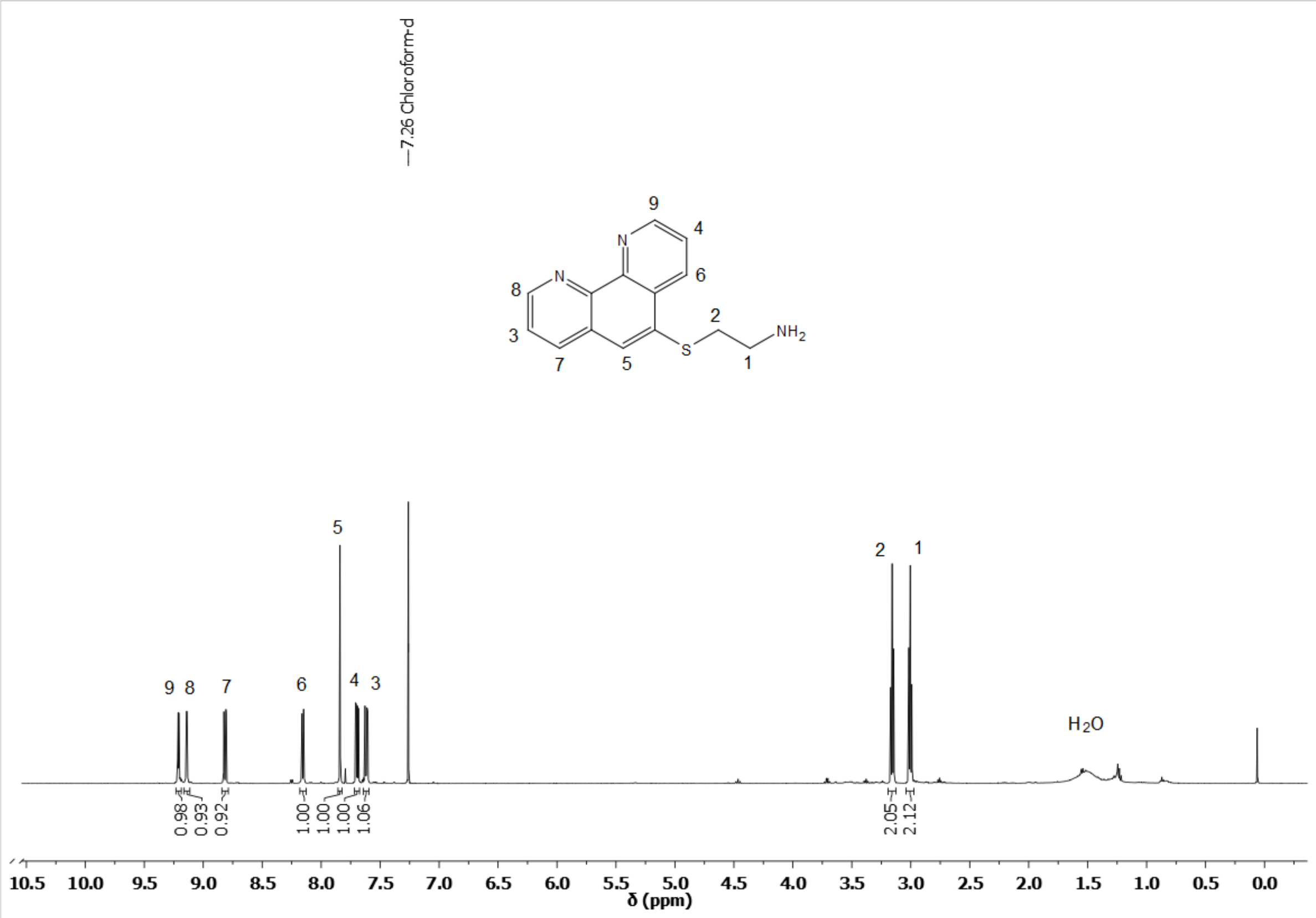

NMR SPECTRUM SI217 | Compound **3**.

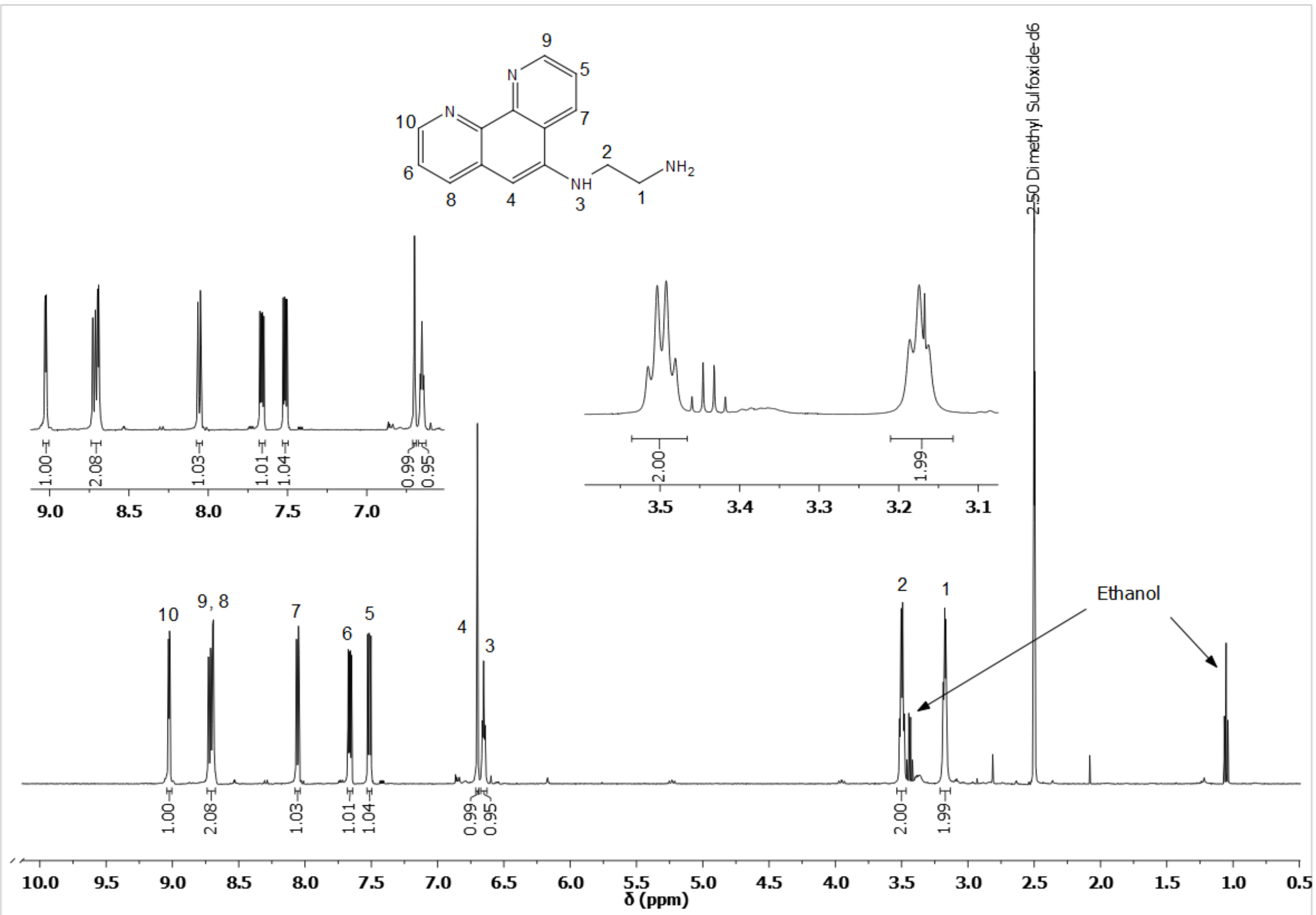

NMR SPECTRUM S318 | Compound **4**.

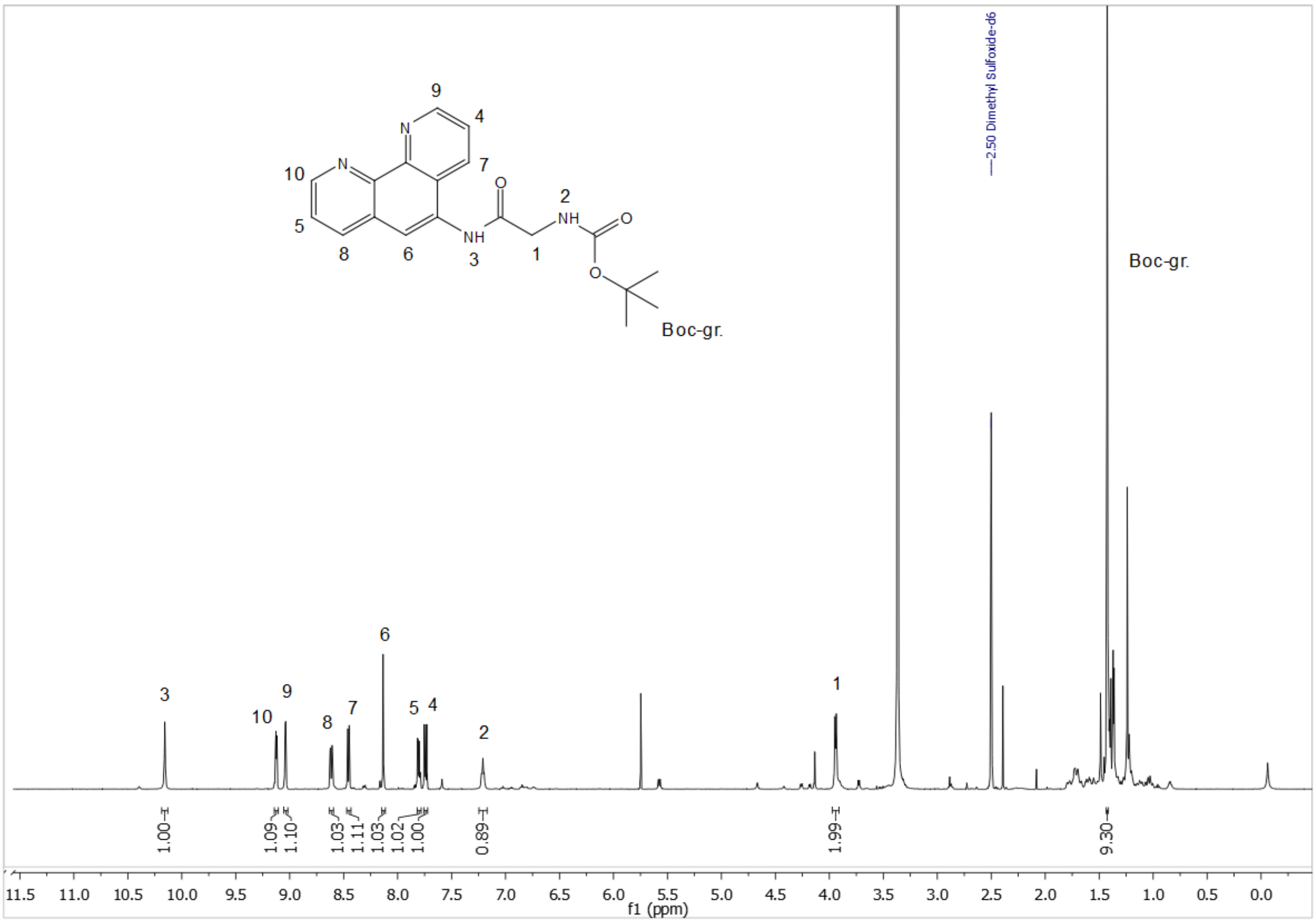

NMR SPECTRUM S419 | Compound **5**.

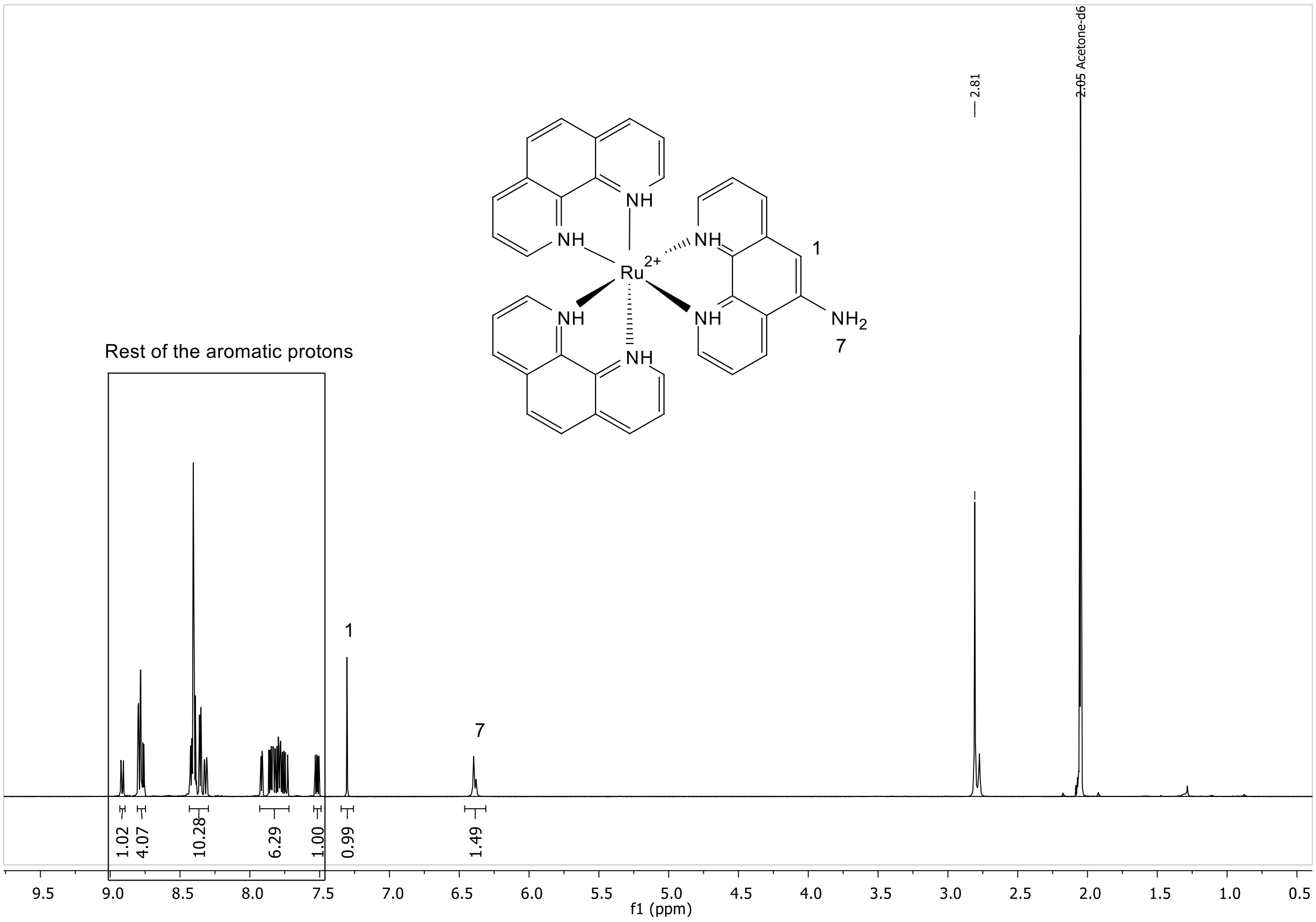

NMR SPECTRUM S520 | Compound **6**.

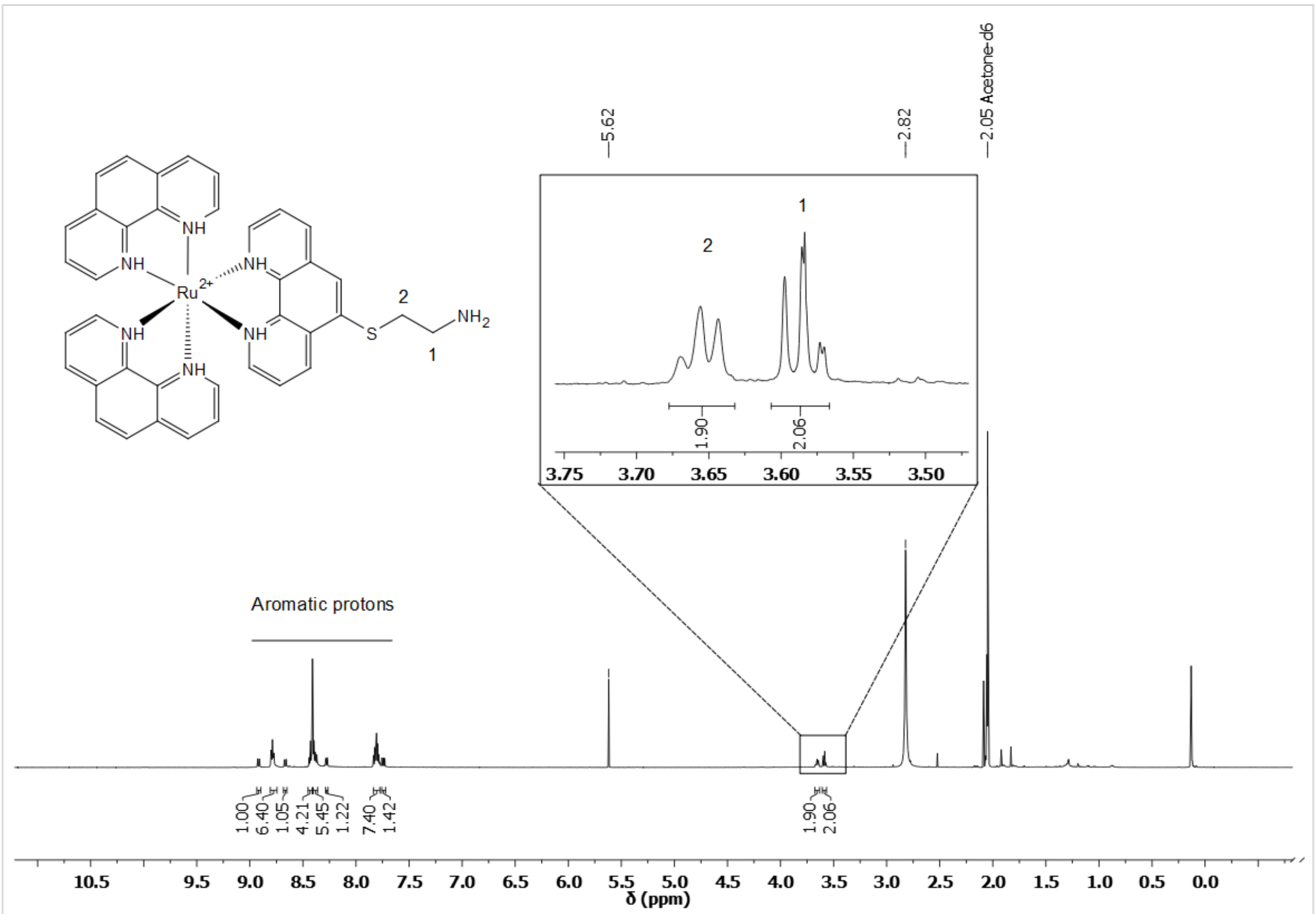

NMR SPECTRUM S621 | Compound **7**.

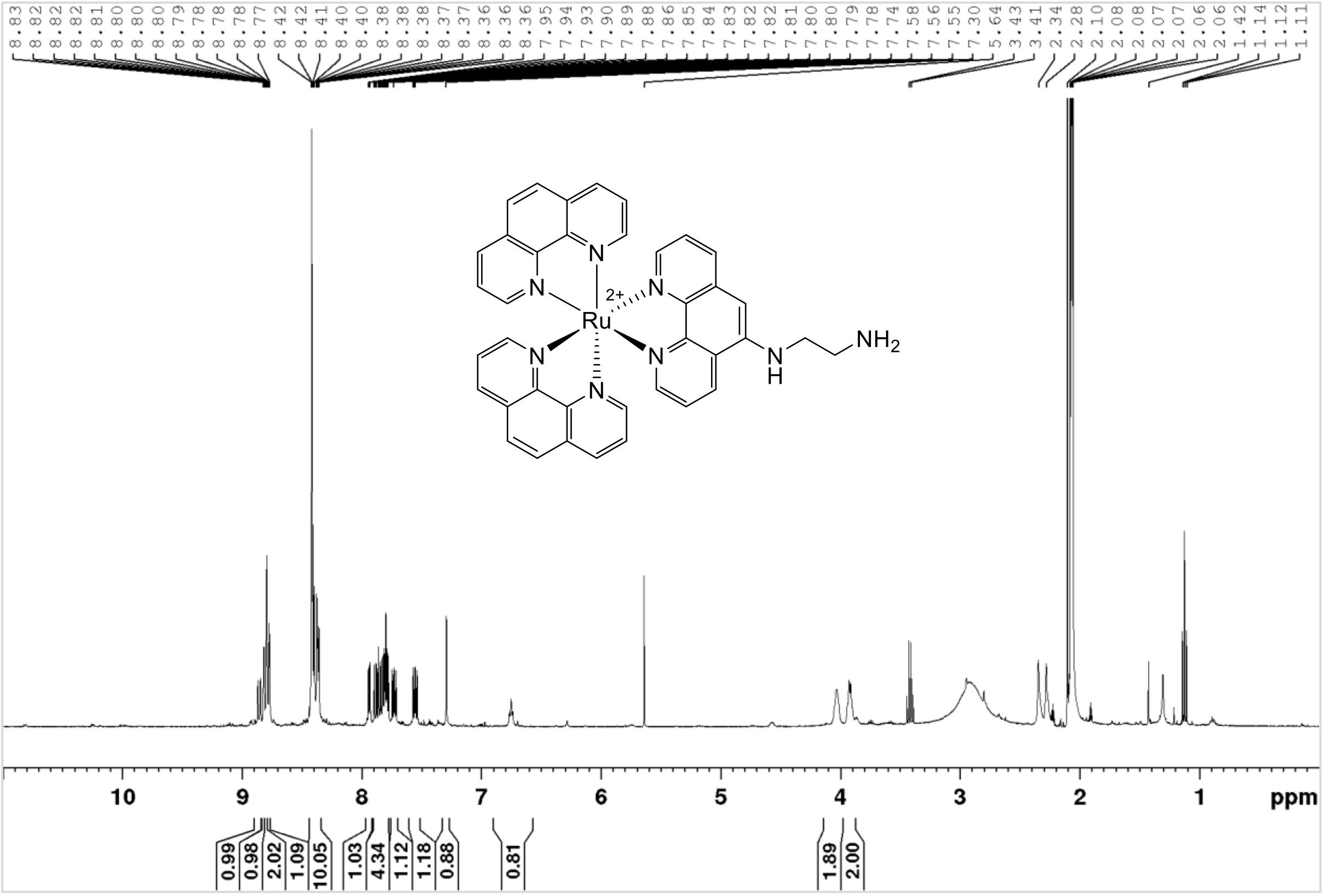

NMR SPECTRUM S722 | Compound **8**.

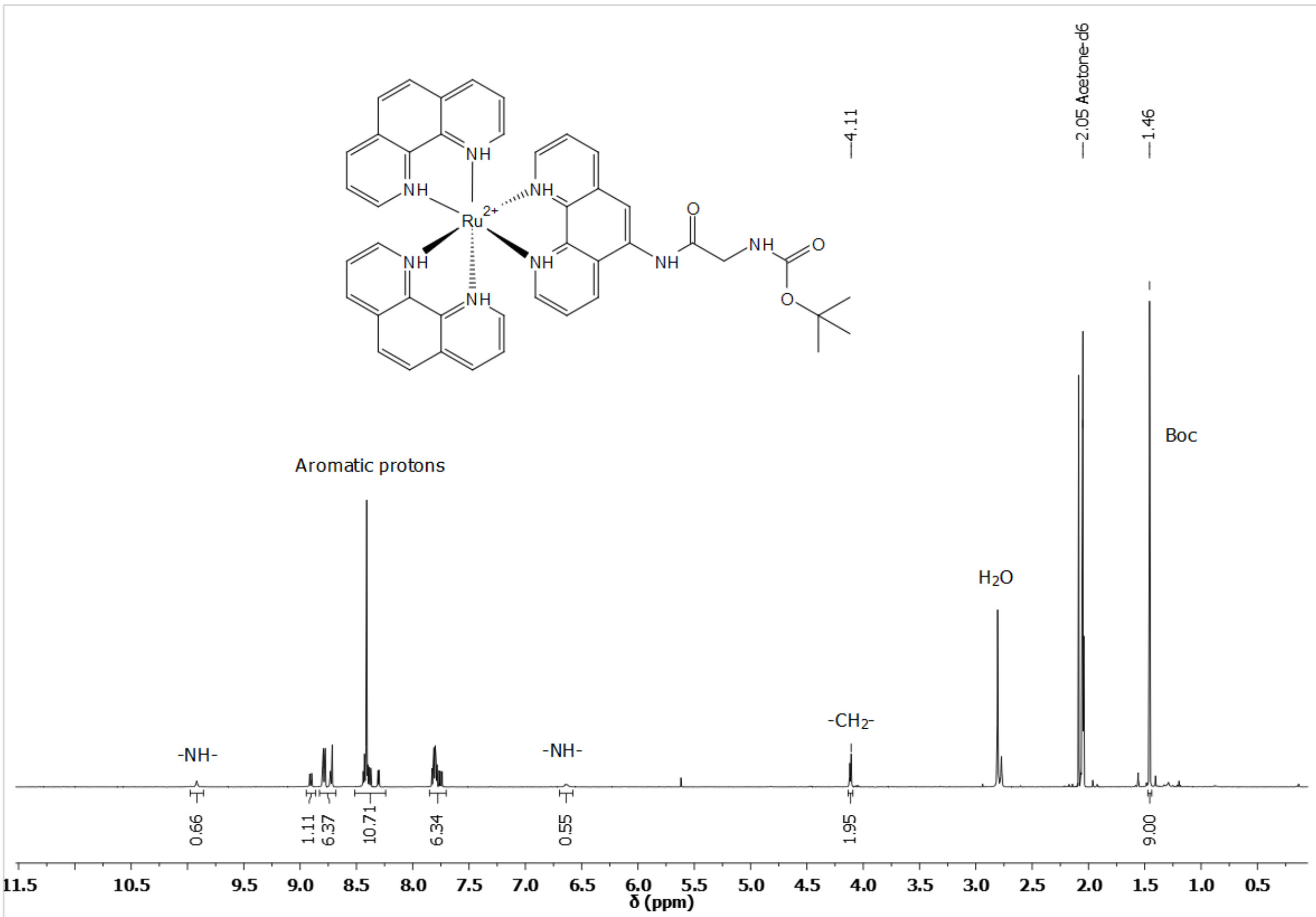

NMR SPECTRUM S823 | Compound **9**.

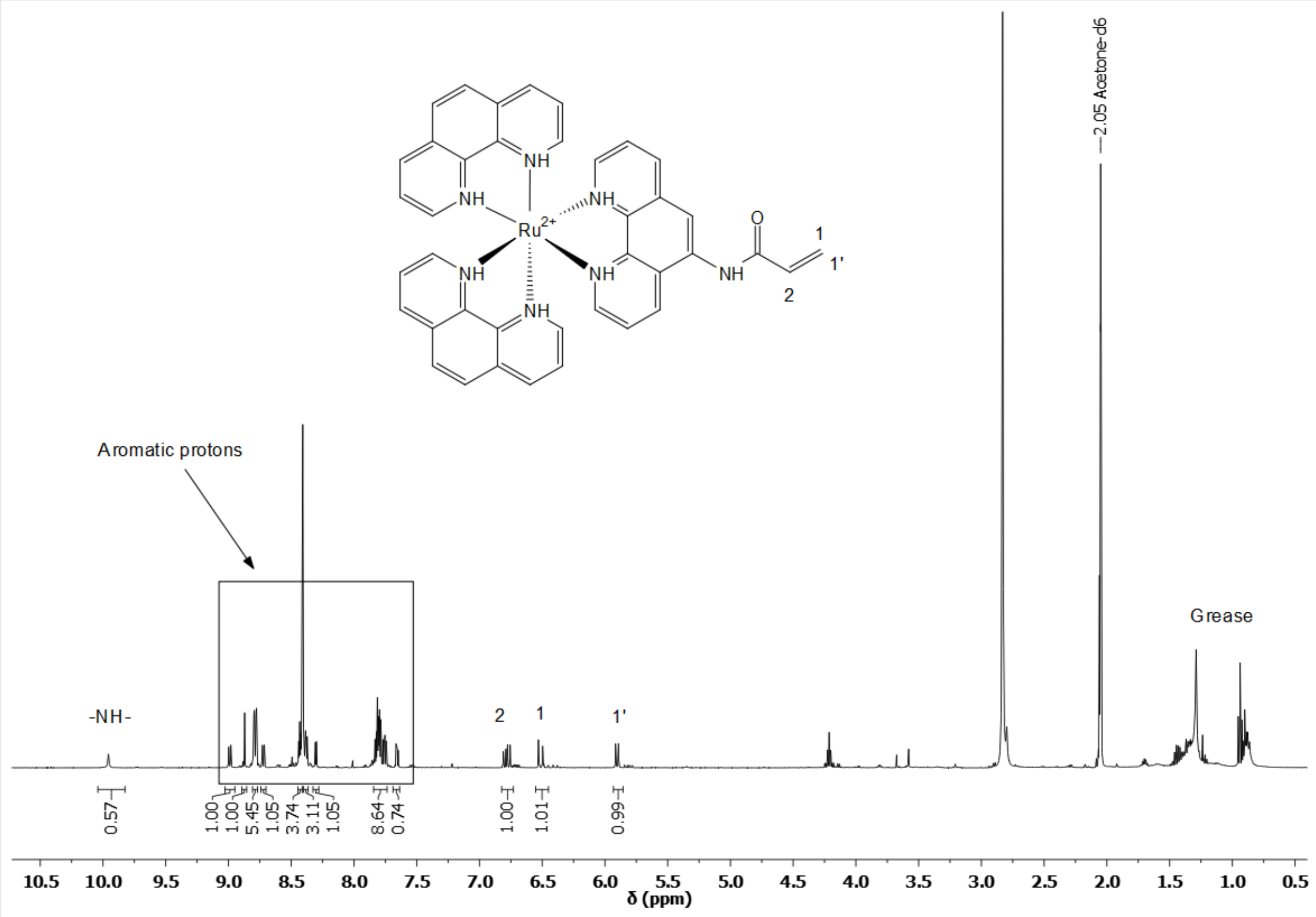

NMR SPECTRUM S924 | Compound **10**.

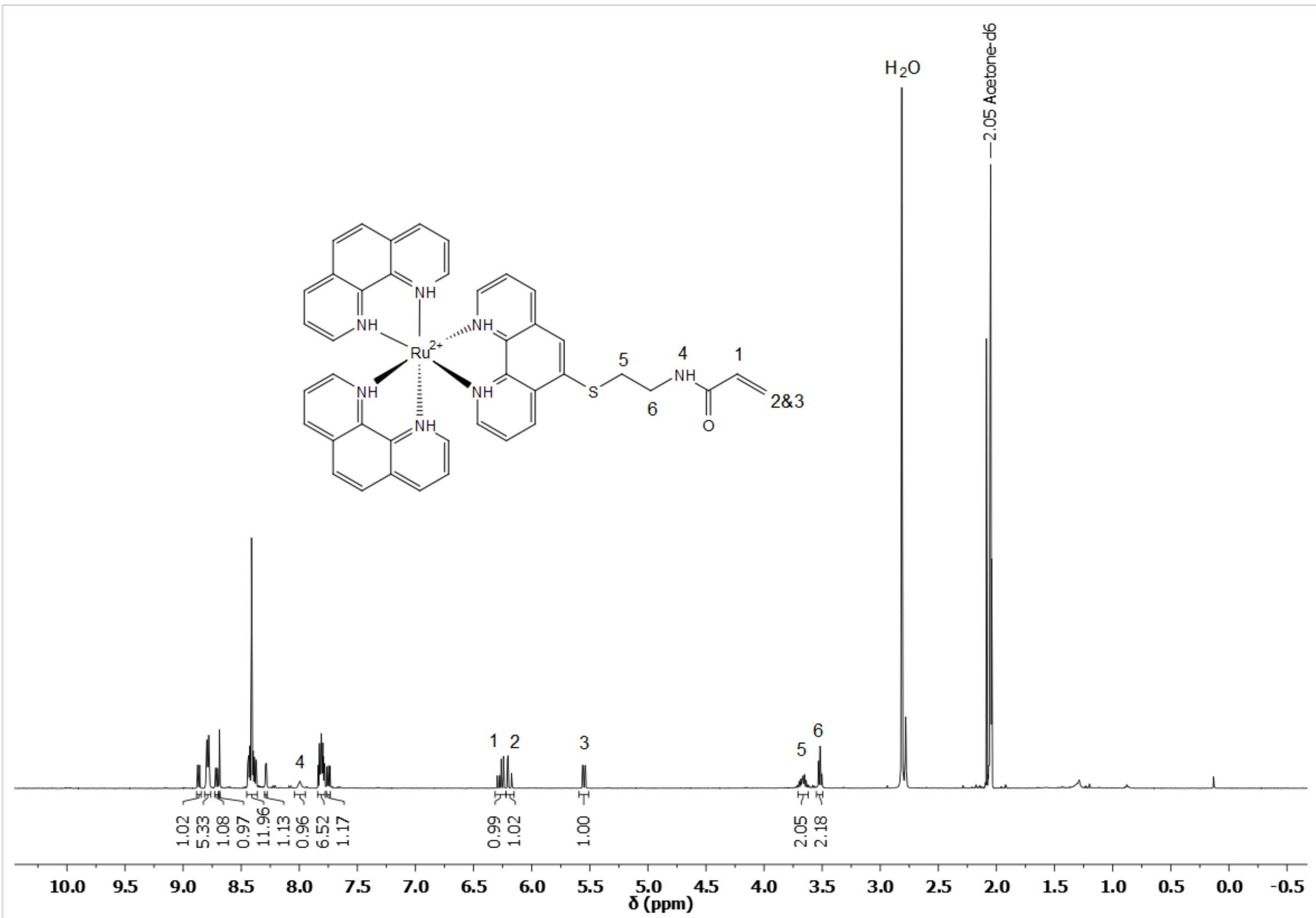

NMR SPECTRUM S1025 | Compound **11**.

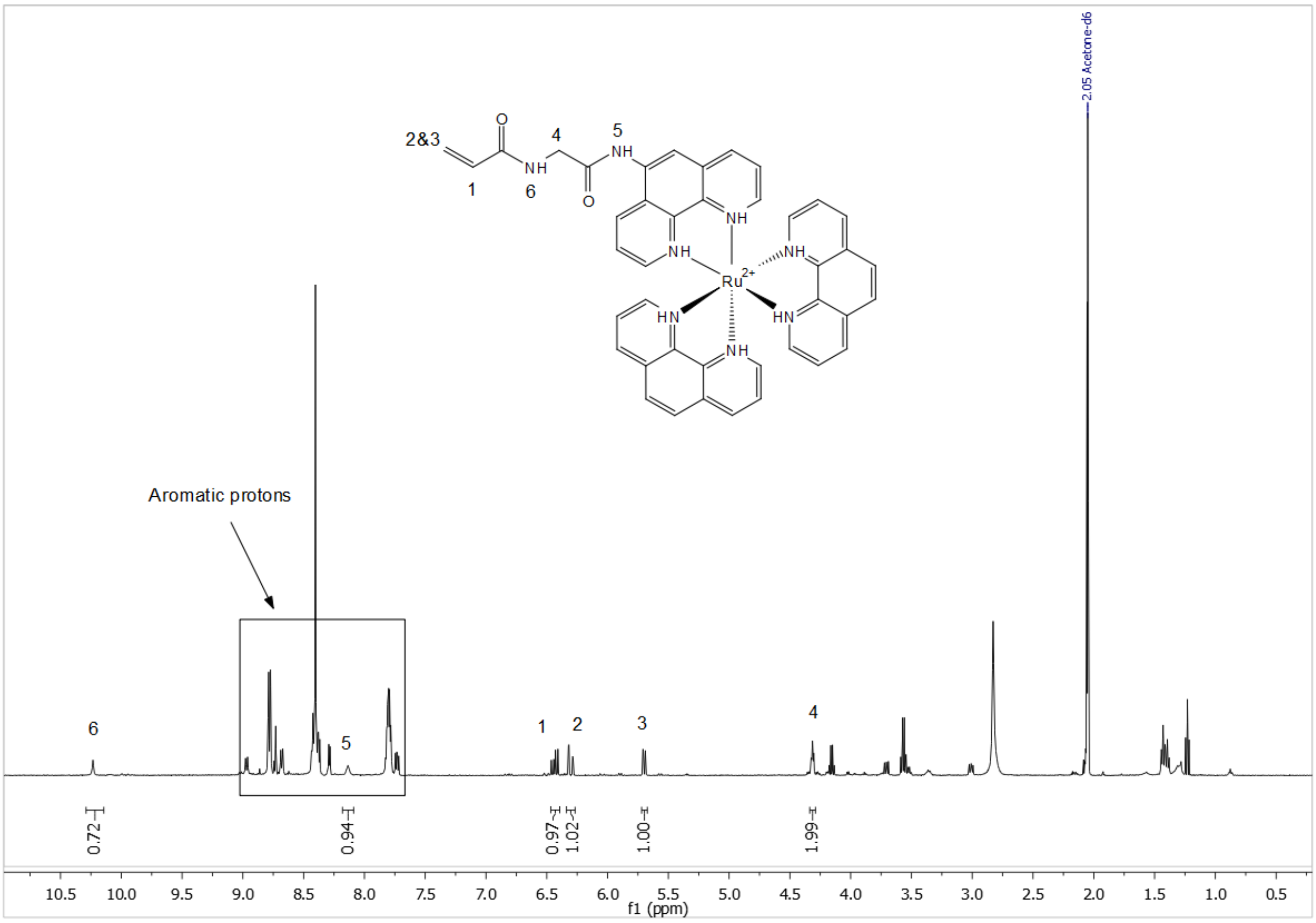

NMR SPECTRUM S1126 | Compound **12**.

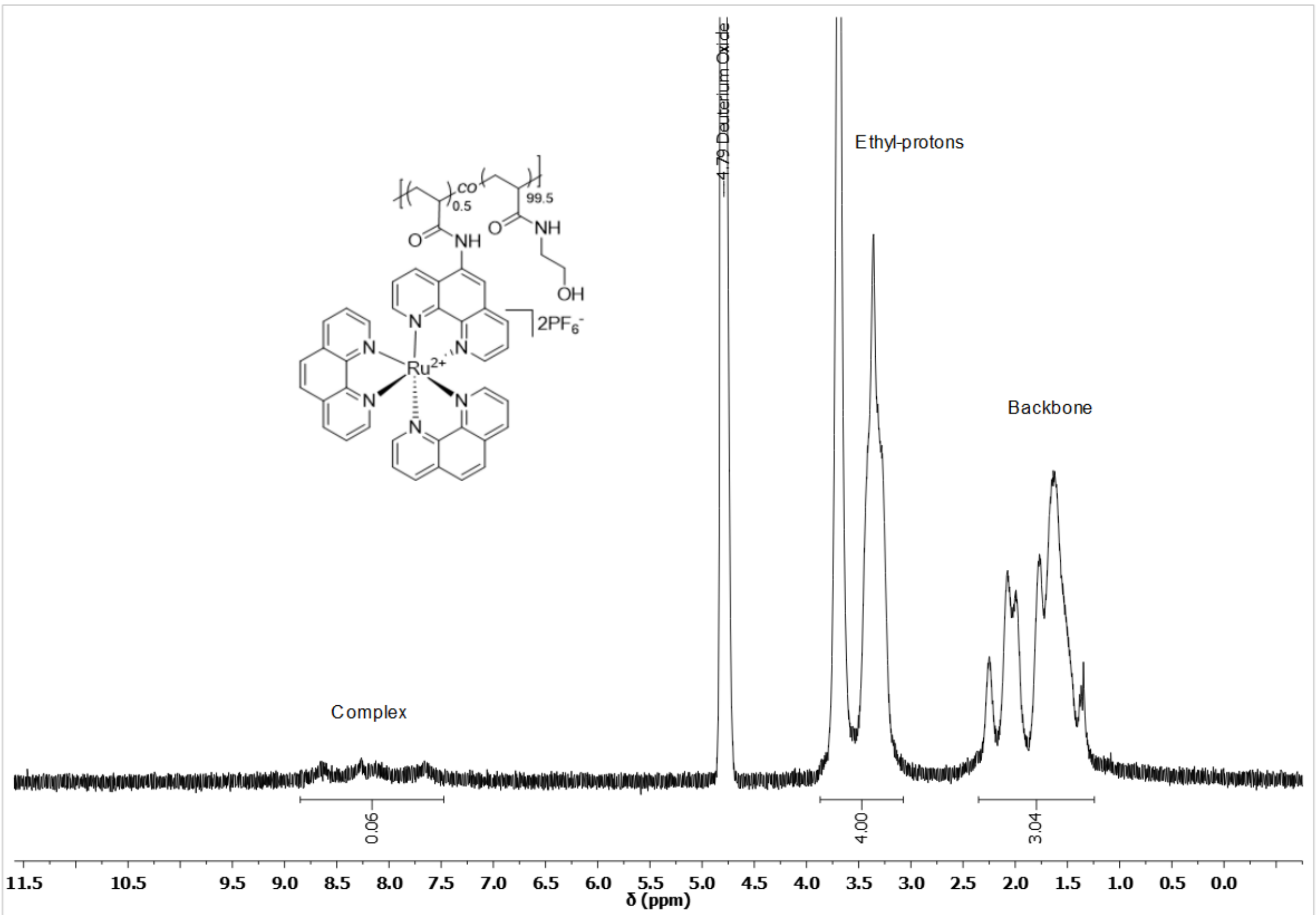

NMR SPECTRUM S1227 | Compound **13**.

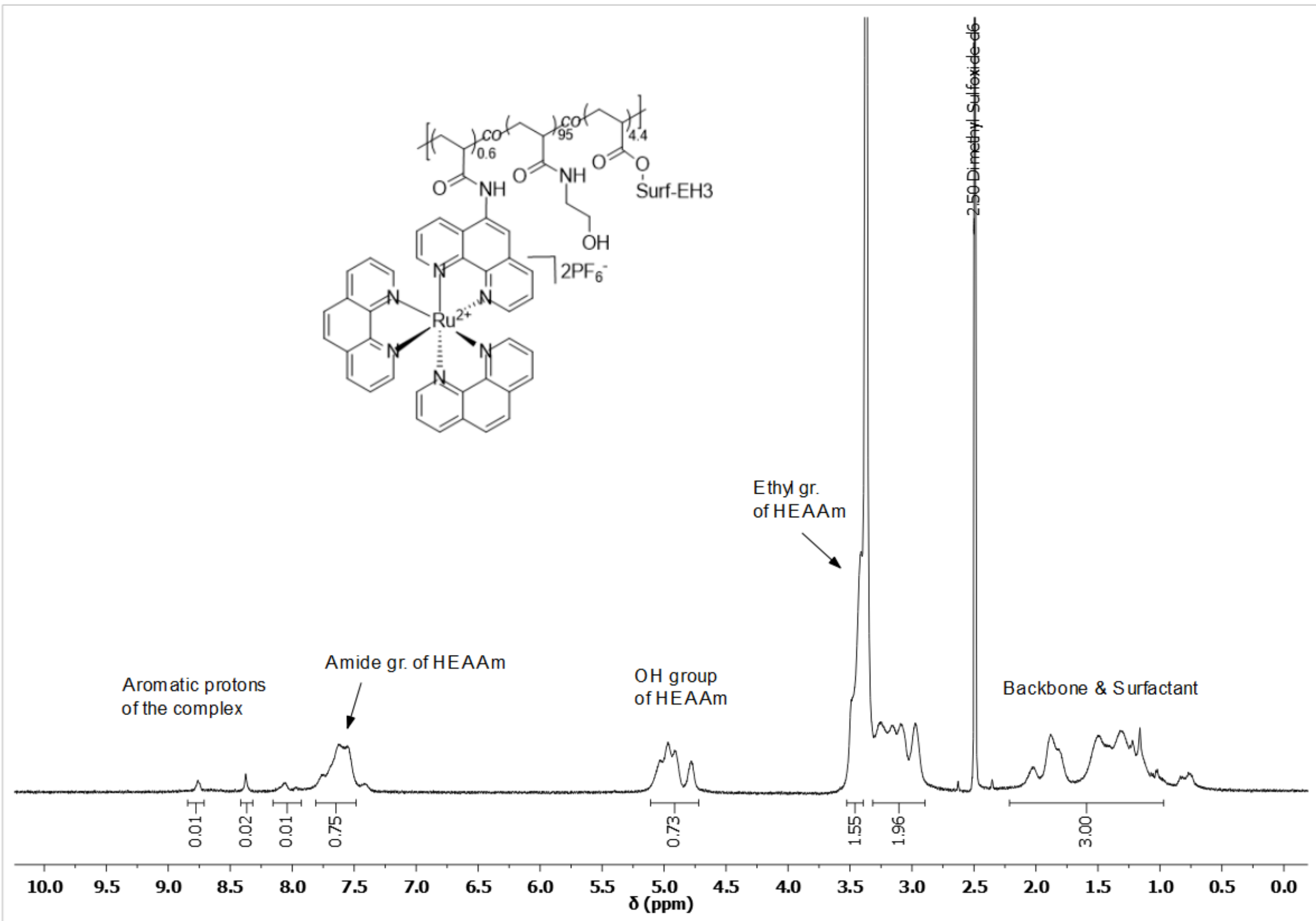

NMR SPECTRUM S1328 | Compound **14**.

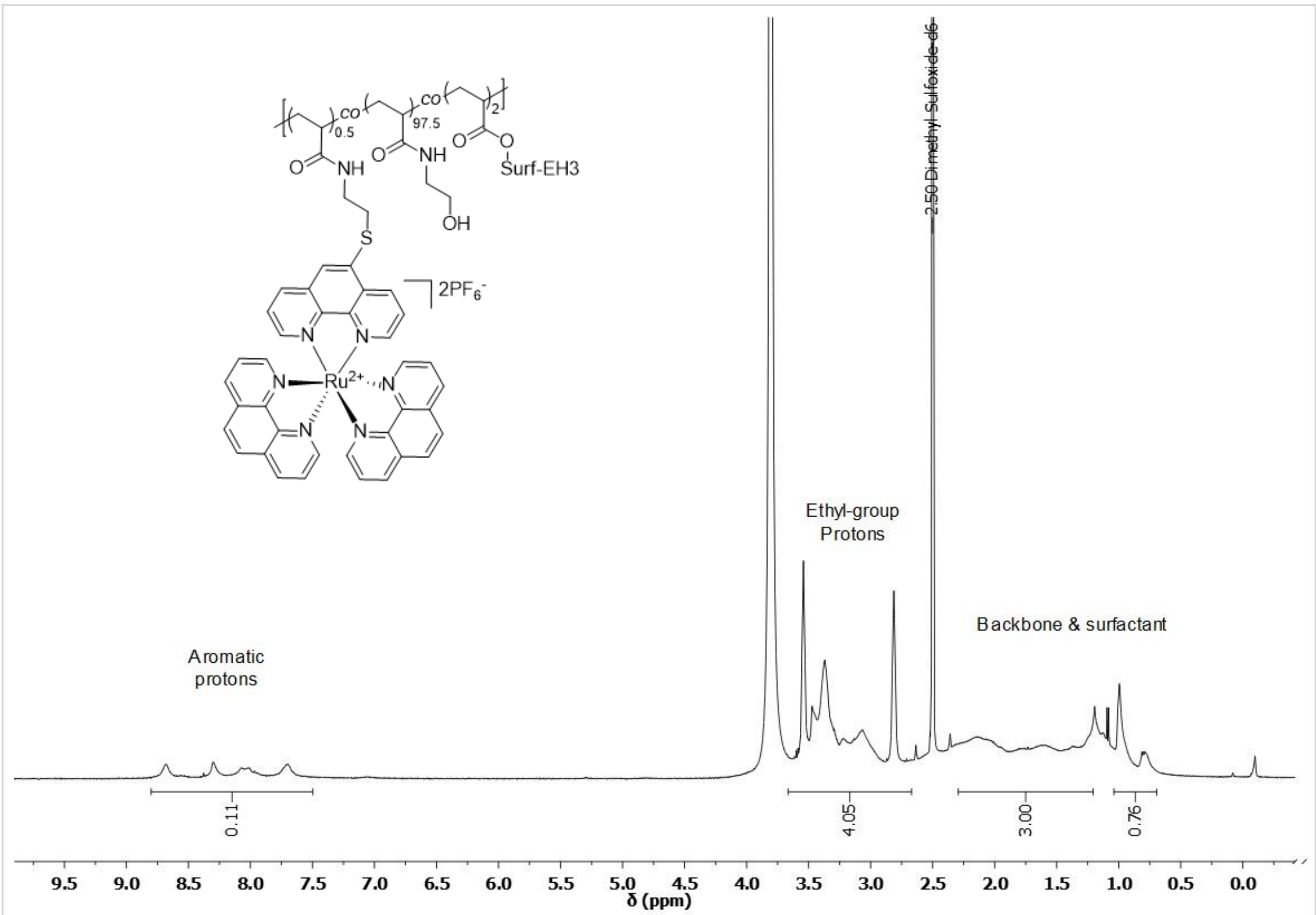

NMR SPECTRUM S1429 | Compound **15**.

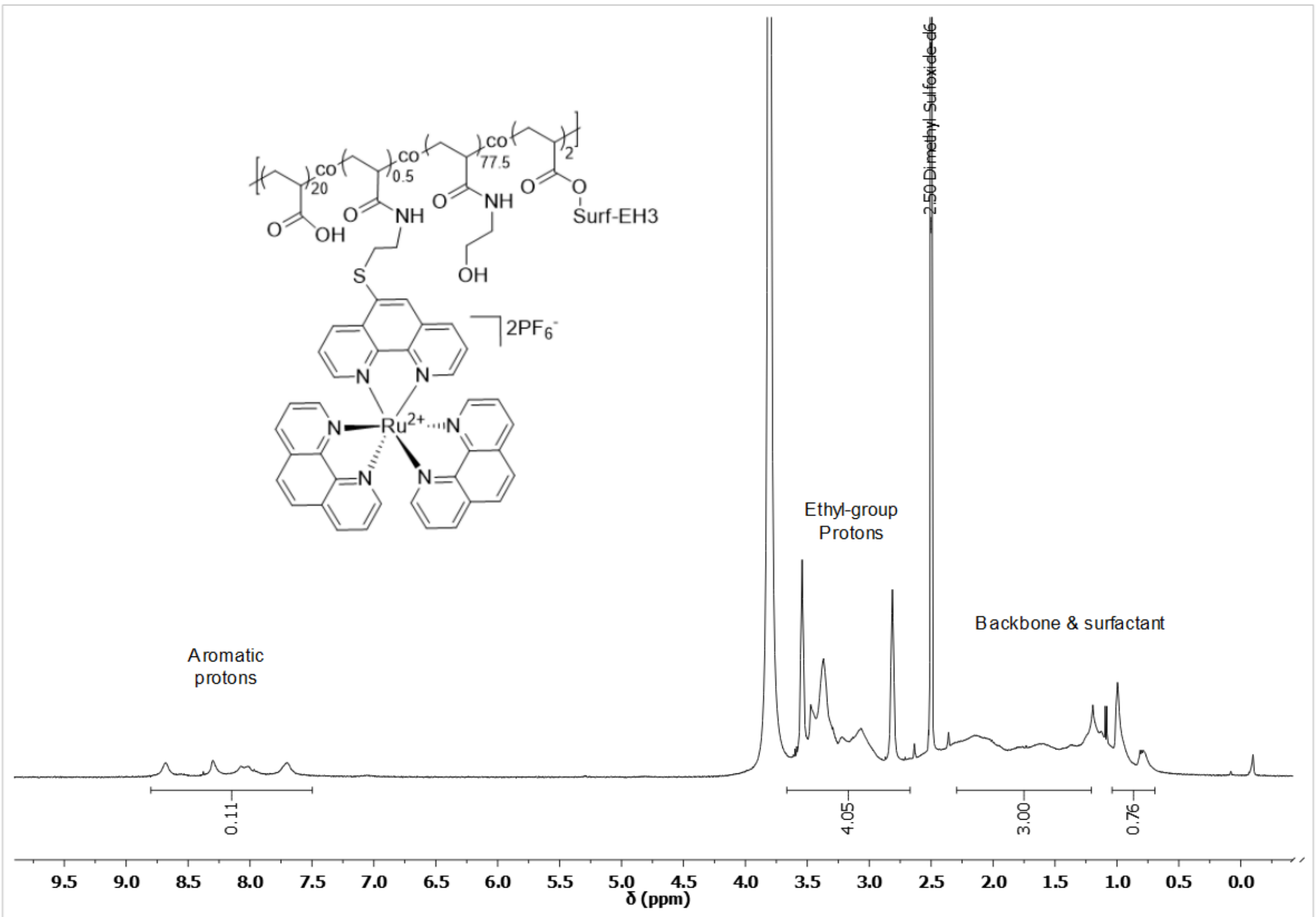

NMR SPECTRUM S1530 | Compound **16**.

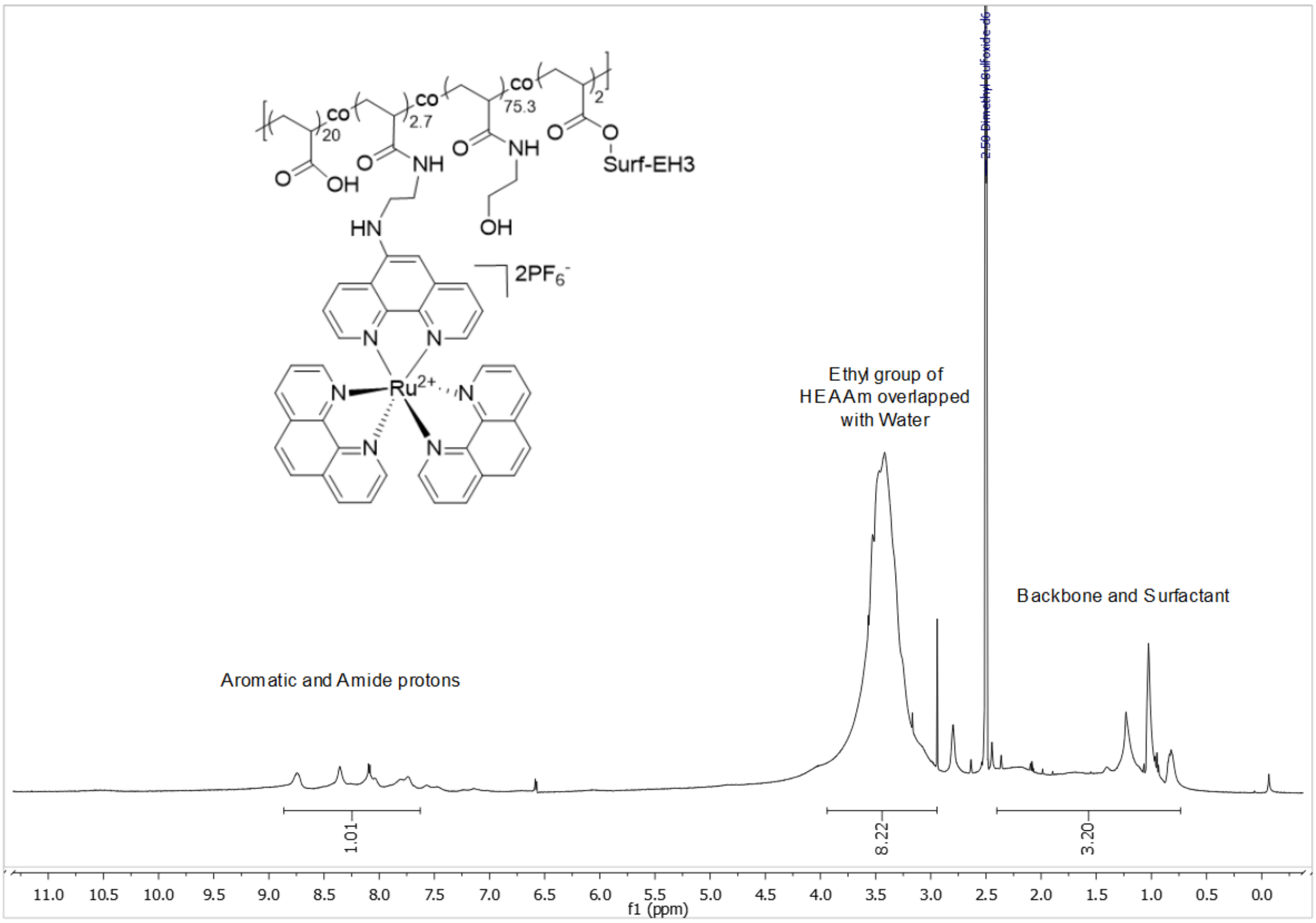

NMR SPECTRUM S1631 | Compound **17**.

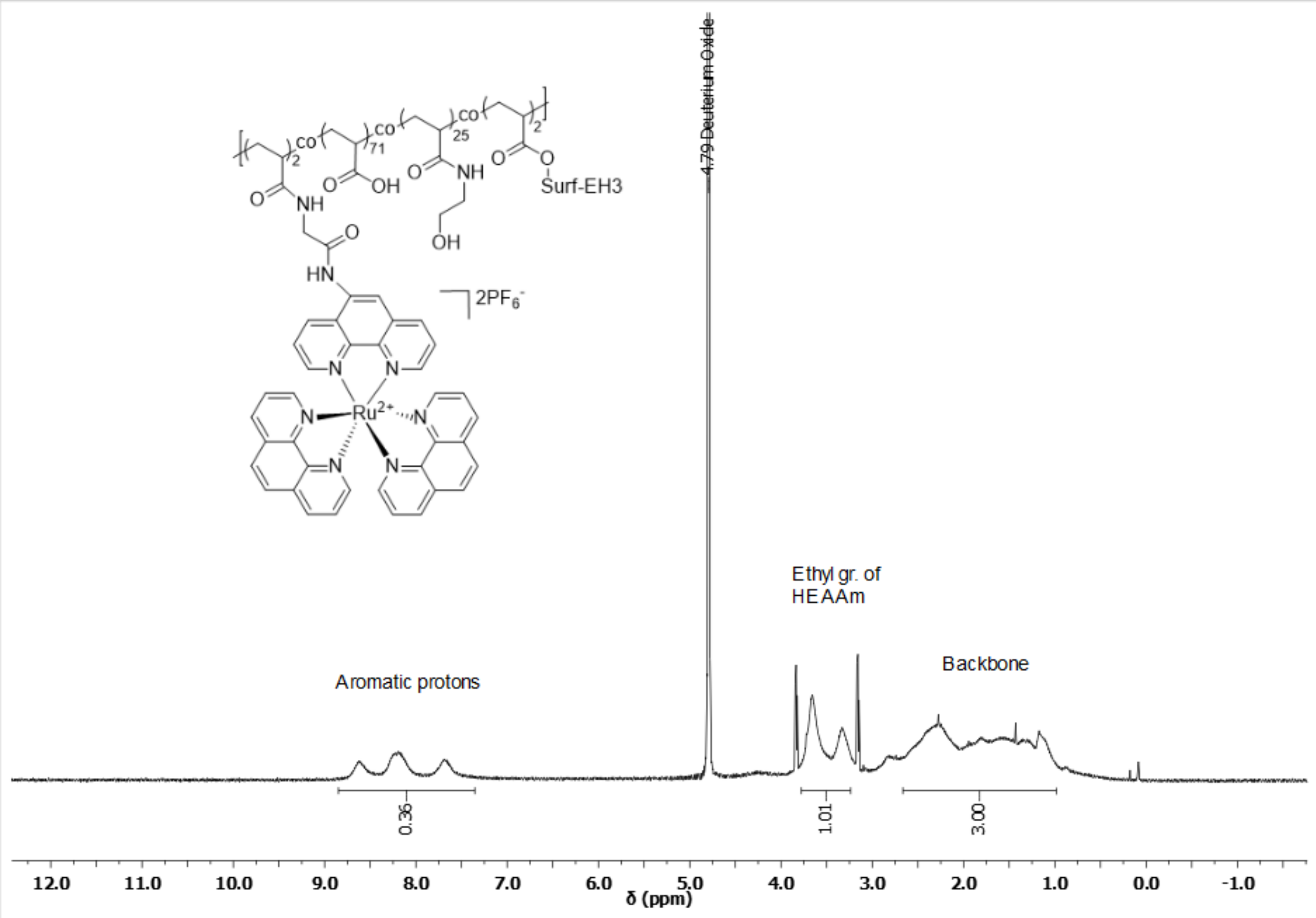

NMR SPECTRUM S1732 | Compound **18**.

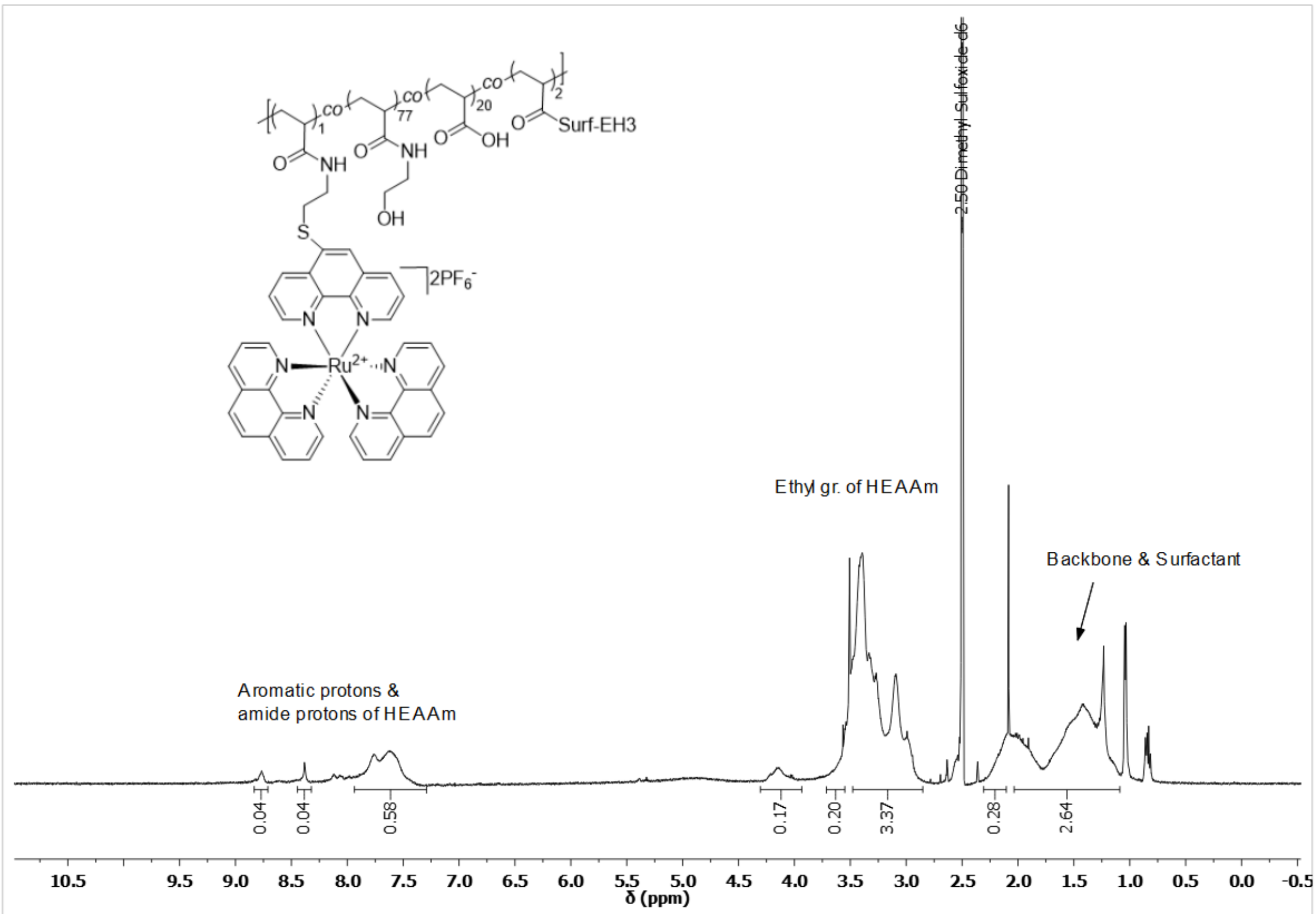

NMR SPECTRUM S1833 | Compound **19**.

A) ^{19}F NMR before PPM

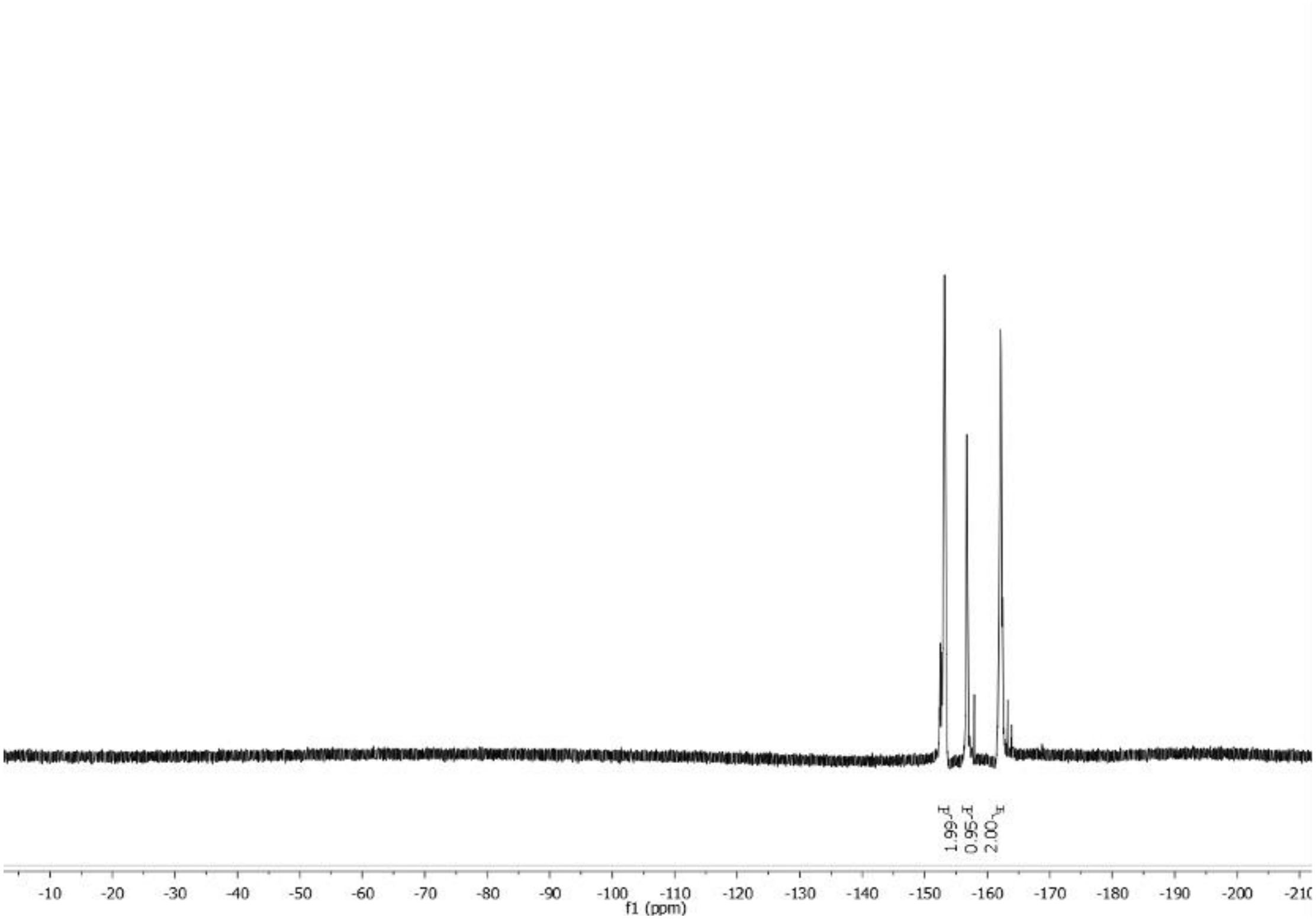

B) ^{19}F NMR after PPM

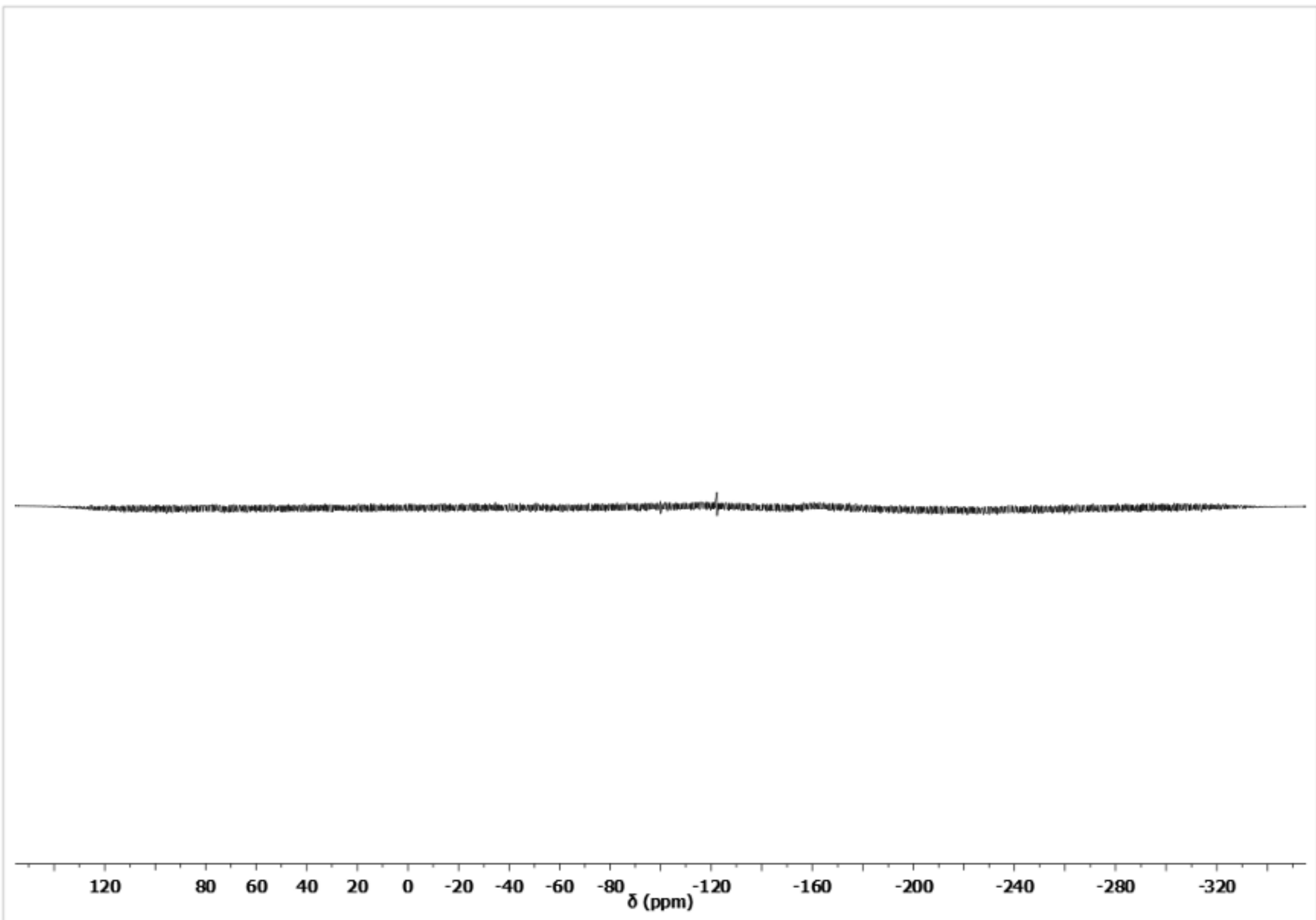

NMR SPECTRUM S1934 | ^{19}F NMR before and after PPM. Importantly, the signals that were clearly visible before PPM (**Panel A**) are no longer detectable after PPM (**Panel B**).

S4 | Supplementary GPC data

Translation:

Probe: Sample
Integration von: Integration start / Integration from
Integration bis: Integration stop / Integration to
Kalibration: Calibration
MHK - A (Kal.): Mark-Houwink-Kuhn (MHK) parameter - A (Calibration)
Int.Stand.-K: Internal Standard Concentration (Int. Std. Conc.)
Pumpe: Pump
Konzentration: Concentration
Säule 1: Column 1
Detektor 1: Detector 1
Detektor 2: Detector 2
Detektor 3: Detector 3
Operateur: Operator / Analyst

Eluent: Eluent / Mobile Phase
MHK - K (Kal.): Mark-Houwink-Kuhn (MHK) parameter - K (Calibration)
Int.Stand.-M: Internal Standard Mass / Amount (Int. Std. Amt.)
Flußrate: Flow Rate
Injektvolumen: Injection Volume
Temperatur: Temperature
Versatz: Offset / Delay
Messintervall: Measurement Interval / Sampling Rate

Probe: Sample
Basislinie von: Baseline of
Integration von: Integration of
Kalibration: Calibration
MHK - A (Kal.): Mark-Houwink-Kuhn (MHK) parameter - A (Calibration)
Int.Stand.-K: Internal Standard Concentration (Int. Std. Conc.)
Pumpe: Pump
Konzentration: Concentration
Säule 1: Column 1
Detektor 1: Detector 1
Detektor 2: Detector 2
Detektor 3: Detector 3
Operateur: Operator

Methode: Method
bis: to / until (used for time or range intervals, e.g., "0 to 10 min")
Eluent: Eluent / Mobile Phase
MHK - K (Kal.): Mark-Houwink-Kuhn (MHK) parameter - K (Calibration)
Flußrate: Flow Rate
Injektvolumen: Injection Volume
Temperatur: Temperature
Versatz: Offset / Delay (often refers to detector offset or signal shift)
Messintervall: Measurement Interval / Sampling Rate

Correspondence used:

Laboratory journal code	Manuscript polymer numbering
BAA 109	**13**
BAA 105	**14**
FRJ 100	**15**
BAA 113	**16**
BAA 095	**17**
BAA 118	**18**
FRJ 101	**19**

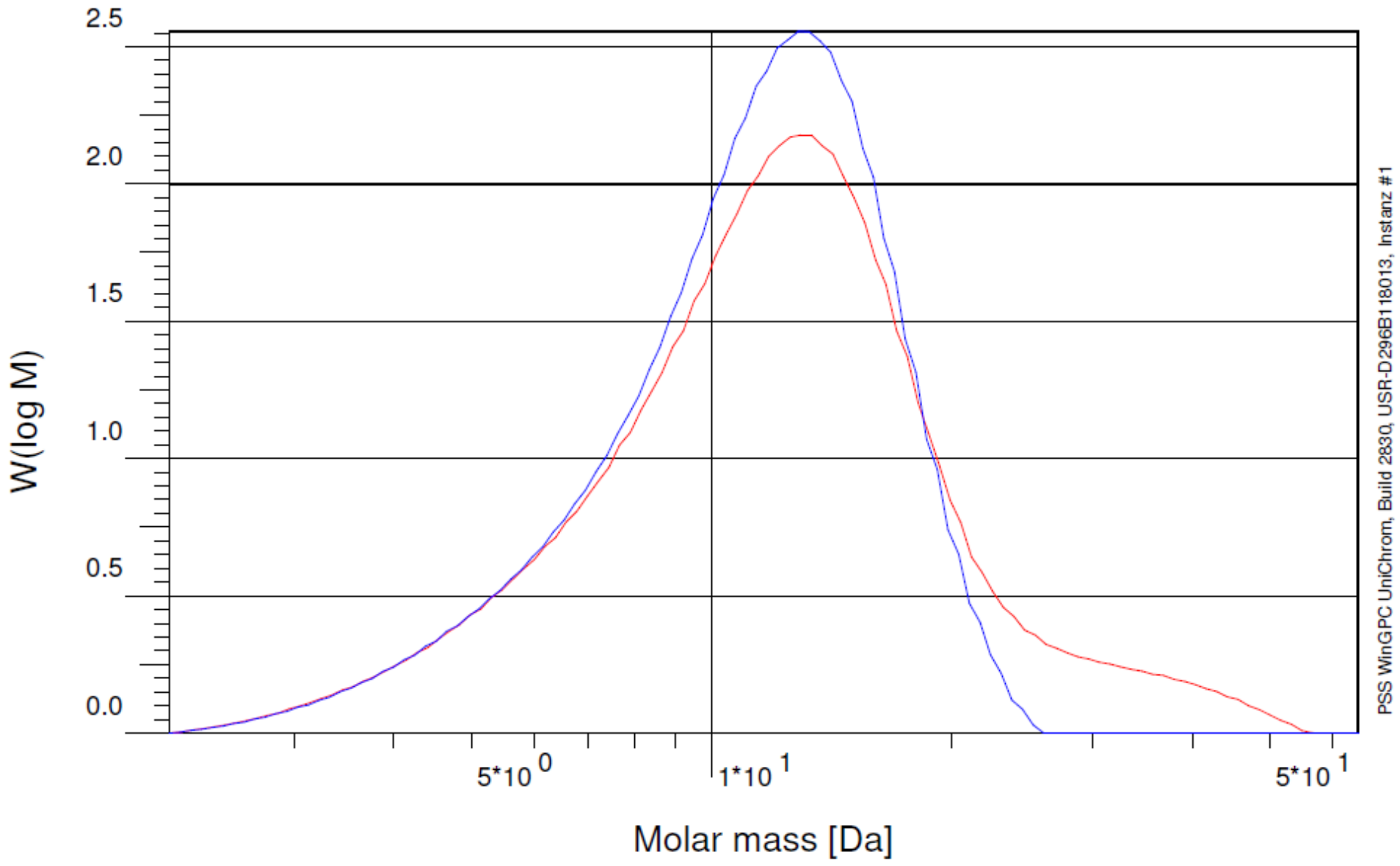

**Probe :**	Vial 21: FRJ-Ru(S) - 1		
**Integration von :**	Donnerstag 06.08.26 04:44:35		14.664 ml
**Integration bis :**	Donnerstag 06.08.26 04:46:27		16.523 ml
**Kalibration :**	120326_GRAM linM DMAcPMMA.cal	**Eluent :**	DMAc + LiBr 0.1
**MHK - A (Kal.):**	0.000E+0	**MHK - K (Kal.):**	1.000E+0 ml/g
**Int.Stand.-K :**	13.727 ml	**Int.Stand.-M :**	------- ml
**Pumpe :**	PSS SECcurity	**Flußrate :**	1.000 ml/min
**Konzentration :**	0.000 g/l	**Injektvolumen :**	20.000 ul
**Säule 1 :**	PSS GRAM LinM	**Temperatur :**	60.000 °C
**Detektor 1 :**	I1: IsoPump 1, Pressure	**Versatz :**	0.000 ml
**Detektor 2 :**	I1: RID 1, RI Signal	**Versatz :**	0.170 ml
**Detektor 3 :**	I1: VWD 1, Signal A	**Versatz :**	0.000 ml
**Operateur :**	usr	**Messintervall :**	1.000 sec

	**I1: RID 1, RI Signal**	**Unsicherheit [%]**	**I1: VWD 1, Signal A**	**Unsicherheit [%]**	
**Mn :**	1.0255e1	n/v	9.7998e0	50.10	g/mol
**Mw :**	1.3365e1	n/v	1.1671e1	50.10	g/mol
**Mz :**	1.7439e1	n/v	1.3244e1	50.10	g/mol
**Mv :**	0.000000	n/v	0.000000	50.10	g/mol
**D :**	1.3032e0	n/v	1.1910e0	70.85	
**[n]:**	0.000000	n/v	0.000000	0.00	ml/g
**Vp :**	1.5535e1	n/v	1.5538e1	3.16	ml
**Mp :**	1.2934e1	n/v	1.2854e1	50.10	g/mol
**FI :**	2.0726e3	n/v	4.6427e2	3.16	ml*V
**< 2**	0.00	n/v	0.00	3.16	
**w% :**	100.00	n/v	100.00	3.16	
**> 64**	0.00	n/v	0.00	3.16	

**Projekt :**	C:\GPC-Daten\2026\March.LDX	**Kostenstelle :**	
**Datum :**	Donnerstag 06.08.26 08:10:13	**Zeichen :**	

GPC data S135

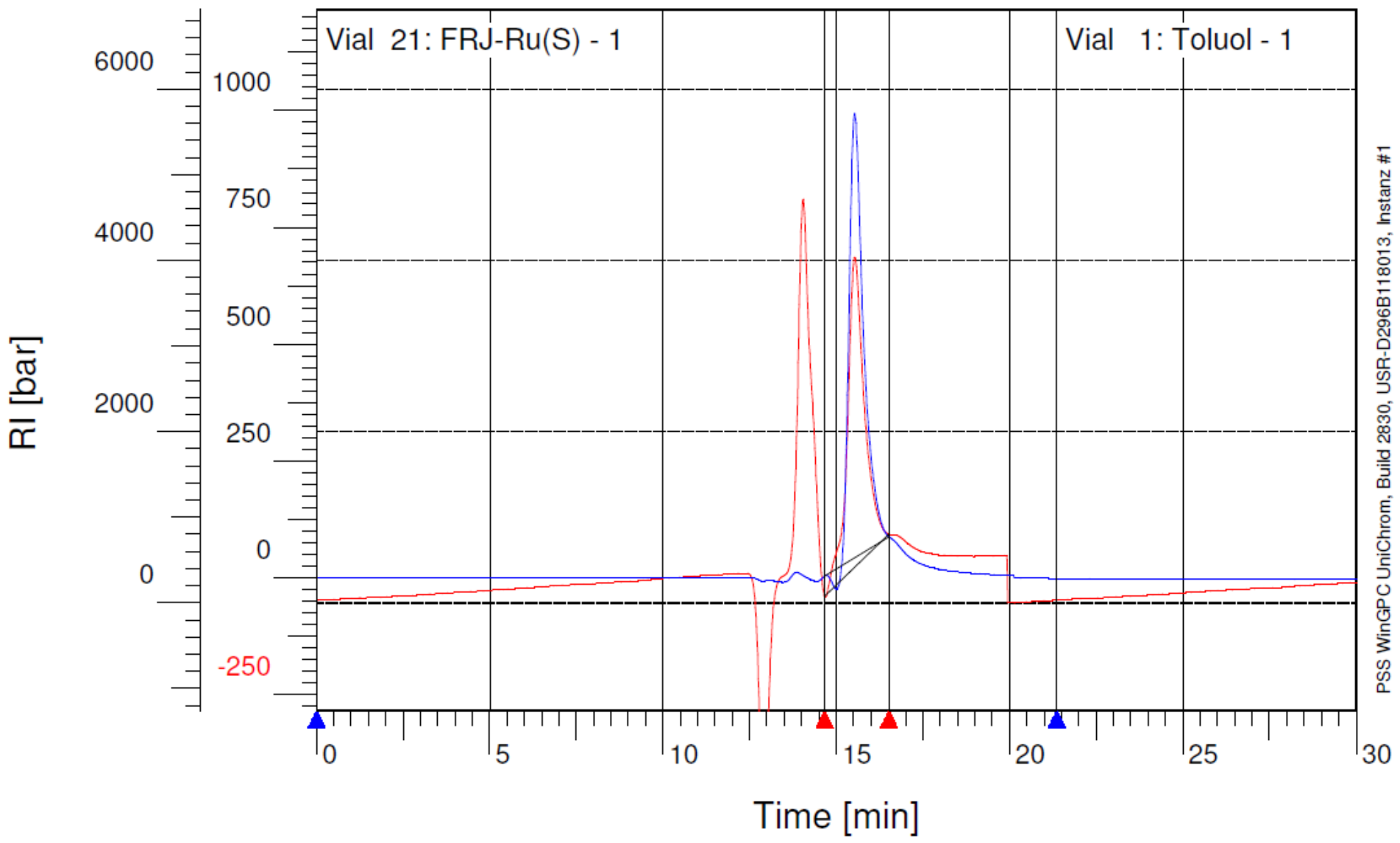

**Probe :**	Vial 21: FRJ-Ru(S) - 1	**Methode :**	C:\wingpc_8#1\Methoden 2025\Kalibr.07012025 (
**Basislinie von :**	14.664 ml	**bis :**	16.523 ml
**Integration von :**	14.664 ml	**bis :**	16.523 ml
**Kalibration :**	120326_GRAM linM DMAcPMMA.cal	**Eluent :**	DMAc + LiBr 0.1
**MHK - A (Kal.):**	0.000E+0	**MHK - K (Kal.):**	1.000E+0 ml/g
**Int.Stand.-K :**	13.727 ml	**Int.Stand.-M :**	------- ml
**Pumpe :**	PSS SECcurity	**Flußrate :**	1.000 ml/min
**Konzentration :**	0.000 g/l	**Injektvolumen :**	20.000 ul
**Säule 1 :**	PSS GRAM LinM	**Temperatur :**	60.000 °C
**Detektor 1 :**	I1: IsoPump 1, Pressure	**Versatz :**	0.000 ml
**Detektor 2 :**	I1: RID 1, RI Signal	**Versatz :**	0.170 ml
**Detektor 3 :**	I1: VWD 1, Signal A	**Versatz :**	0.000 ml
**Operateur :**	usr	**Messintervall :**	1.000 sec

**Projekt :**	C:\GPC-Daten\2026\March.LDX	**Kostenstelle :**	
**Datum :**	Dienstag 11.08.26 11:51:39	**Zeichen :**	

GPC data S236

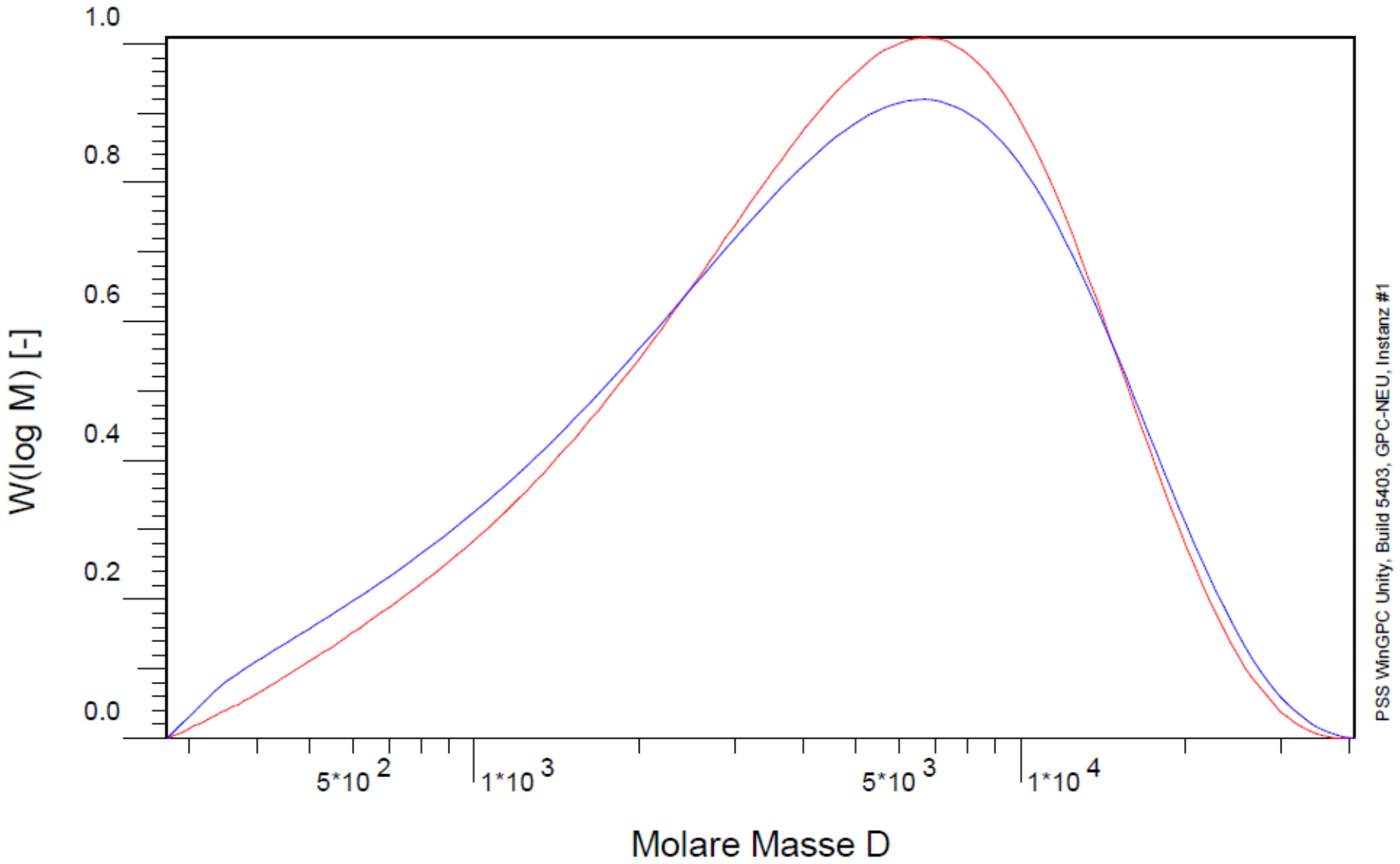

**Probe :**	Vial 72: BAA095		
**Integration von :**	Sonntag 02/28/93 12:00:08		9.526 ml
**Integration bis :**	Sonntag 02/28/93 12:02:49		12.206 ml
**Kalibration :**	15122023_NovemaMax_LinM_water_KCl_Form…Pullulan_A-RI…	**Eluent :**	Water+15%ACN+NaNO3
**MHK - A (Kal.):**	0.000E+0	**MHK - K (Kal.):**	1.000E+0 ml/g
**Int.Stand.-K :**	12.040 ml	**Int.Stand.-M :**	12.040 ml
**Pumpe :**	Agilent 1100	**Flußrate :**	1.000 ml/min
**Konzentration :**	1.000 g/l	**Injektvolumen :**	50.000 ul
**Säule 1 :**	PSS Suprema analytical 1 000A	**Temperatur :**	30.000 C
**Detektor 1 :**	Shodex RI71	**Versatz :**	0.067 ml
**Detektor 2 :**	Agilent DAD 280	**Versatz :**	0.000 ml
**Operateur :**	Admin	**Messintervall :**	1.000 sec

	**Shodex RI71**	**Agilent DAD 280**	
**Mn :**	2.8449e3	2.5268e3	g/mol
**Mw :**	6.4610e3	6.4176e3	g/mol
**Mz :**	1.0756e4	1.1325e4	g/mol
**Mv :**	0.000000	0.000000	g/mol
**D :**	2.2711e0	2.5398e0	
**[n]:**	0.000000	0.000000	ml/g
**Vp :**	1.0487e1	1.0504e1	ml
**Mp :**	6.6641e3	6.4415e3	g/mol
**FI :**	8.996e-3	2.121e-1	ml*V
**< 272**	0.00	0.00	
**w% :**	100.00	100.00	
**> 40814**	0.00	0.00	

**Projekt :**	C:\pss_GPC\GPC-Daten\2024\February.LDX	**Kostenstelle :**	
**Datum :**	Dienstag 08/11/26 12:56:37	**Zeichen :**	

Created with novaPDF Printer (www.novaPDF.com). Please register to remove this message.

GPC data S337

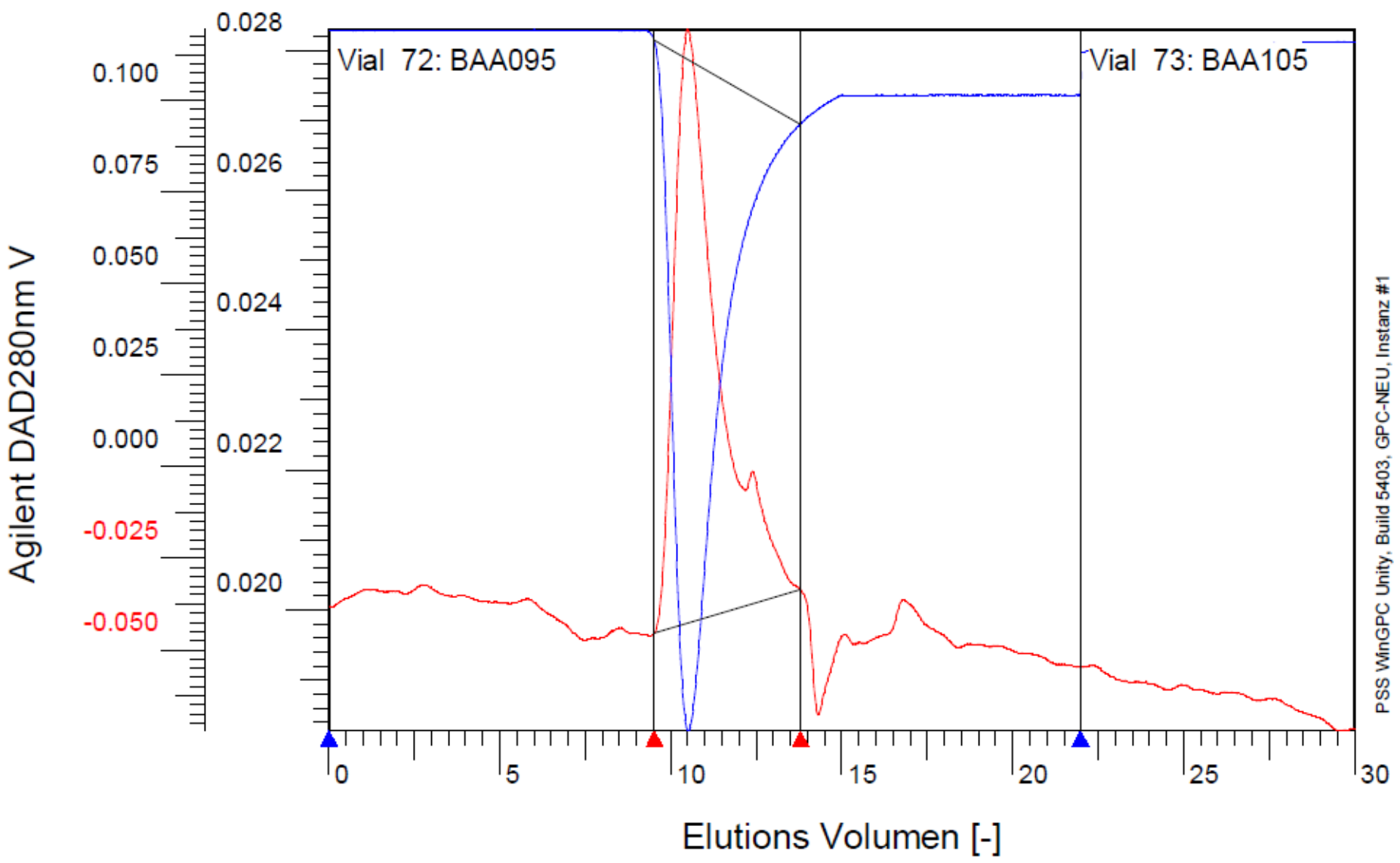

**Probe :**	Vial 72: BAA095	**Methode :**	C:\pss_GPC\Methoden\Methoden Agilent 1100\Water_
**Basislinie von :**	9.526 ml	**bis :**	13.801 ml
**Integration von :**	9.526 ml	**bis :**	13.801 ml
**Kalibration :**	15122023_NovemaMax_LinM_water_KCl_FormBased Pullulan_A-RID.CAL	**Eluent :**	Water+15%ACN+NaNO3
**MHK - A (Kal.):**	0.000E+0	**MHK - K (Kal.):**	1.000E+0 ml/g
**Int.Stand.-K :**	12.040 ml	**Int.Stand.-M :**	12.040 ml
**Pumpe :**	Agilent 1100	**Flußrate :**	1.000 ml/min
**Konzentration :**	1.000 g/l	**Injektvolumen :**	50.000 ul
**Säule 1 :**	PSS Suprema analytical 1 000A	**Temperatur :**	30.000 C
**Detektor 1 :**	Shodex RI71	**Versatz :**	0.067 ml
**Detektor 2 :**	Agilent DAD 280	**Versatz :**	0.000 ml
**Operateur :**	Admin	**Messintervall :**	1.000 sec

**Projekt :**	C:\pss_GPC\GPC-Daten\2024\February.LDX	**Kostenstelle :**	
**Datum :**	Dienstag 08/11/26 12:53:33	**Zeichen :**	

Created with novaPDF Printer (www.novaPDF.com). Please register to remove this message.

GPC data S438

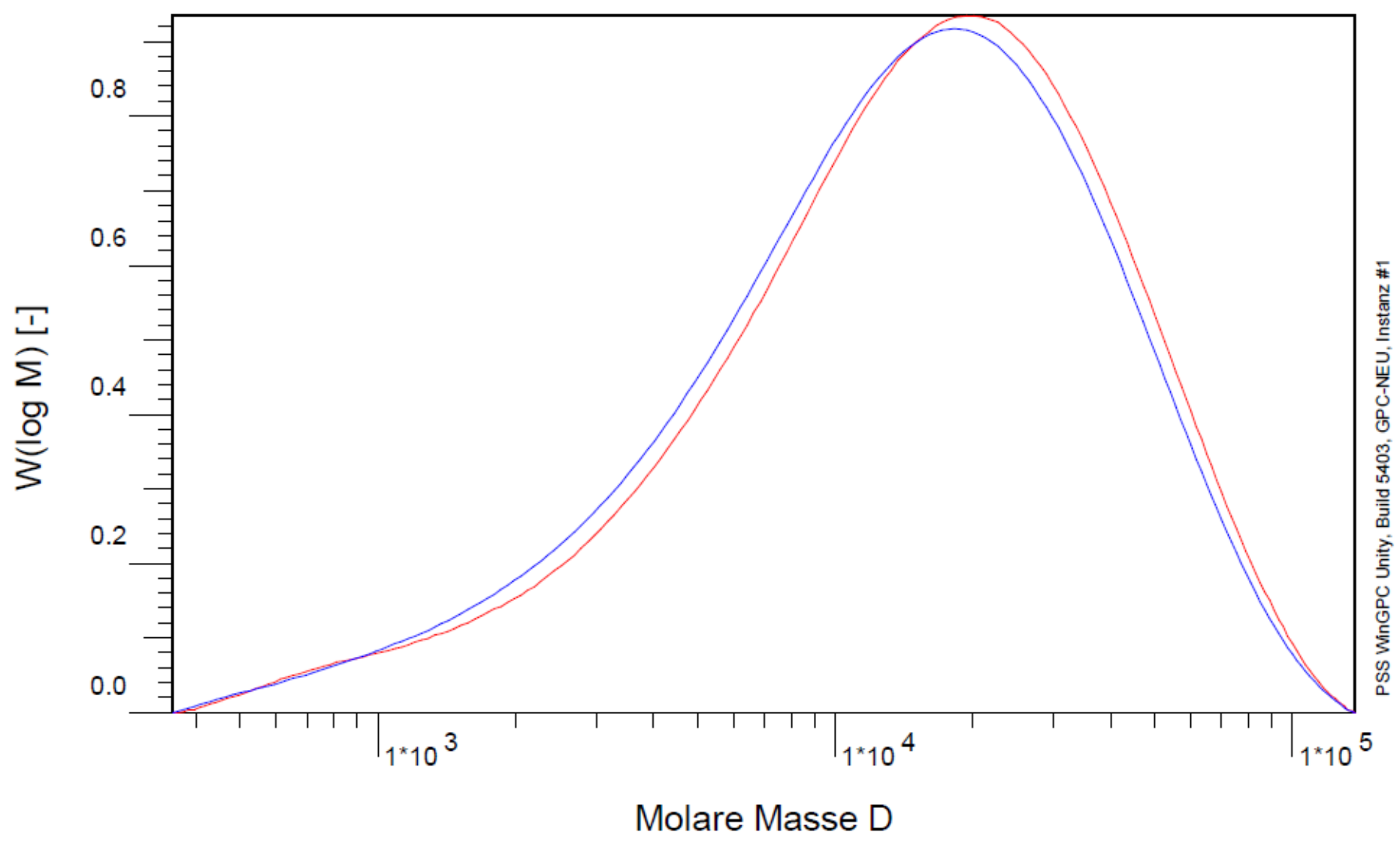

**Probe :**	Vial 73: BAA105		
**Integration von :**	Sonntag 02/28/93 12:21:17		8.696 ml
**Integration bis :**	Sonntag 02/28/93 12:24:36		12.015 ml
**Kalibration :**	15122023_NovemaMax_LinM_water_KCl_FormPullulan_A-RI	**Eluent :**	Water+15%ACN+NaNO3
**MHK - A (Kal.):**	0.000E+0	**MHK - K (Kal.):**	1.000E+0 ml/g
**Int.Stand.-K :**	12.040 ml	**Int.Stand.-M :**	12.040 ml
**Pumpe :**	Agilent 1100	**Flußrate :**	1.000 ml/min
**Konzentration :**	1.000 g/l	**Injektvolumen :**	50.000 ul
**Säule 1 :**	PSS Suprema analytical 1 000A	**Temperatur :**	30.000 C
**Detektor 1 :**	Shodex RI71	**Versatz :**	0.067 ml
**Detektor 2 :**	Agilent DAD 280	**Versatz :**	0.000 ml
**Operateur :**	Admin	**Messintervall :**	1.000 sec

	**Shodex RI71**	**Agilent DAD 280**	
**Mn :**	7.2998e3	6.9068e3	g/mol
**Mw :**	2.1843e4	2.0602e4	g/mol
**Mz :**	3.9578e4	3.8150e4	g/mol
**Mv :**	0.000000	0.000000	g/mol
**D :**	2.9923e0	2.9829e0	
**[n]:**	0.000000	0.000000	ml/g
**Vp :**	9.9723e0	1.0023e1	ml
**Mp :**	1.8242e4	1.6575e4	g/mol
**FI :**	7.711e-3	7.259e-2	ml*V
**< 354**	0.00	0.00	
**w% :**	100.00	100.00	
**> 136525**	0.00	0.00	

**Projekt :**	C:\pss_GPC\GPC-Daten\2024\February.LDX	**Kostenstelle :**	
**Datum :**	Dienstag 08/11/26 12:58:52	**Zeichen :**	

Created with novaPDF Printer (www.novaPDF.com). Please register to remove this message.

GPC data S539

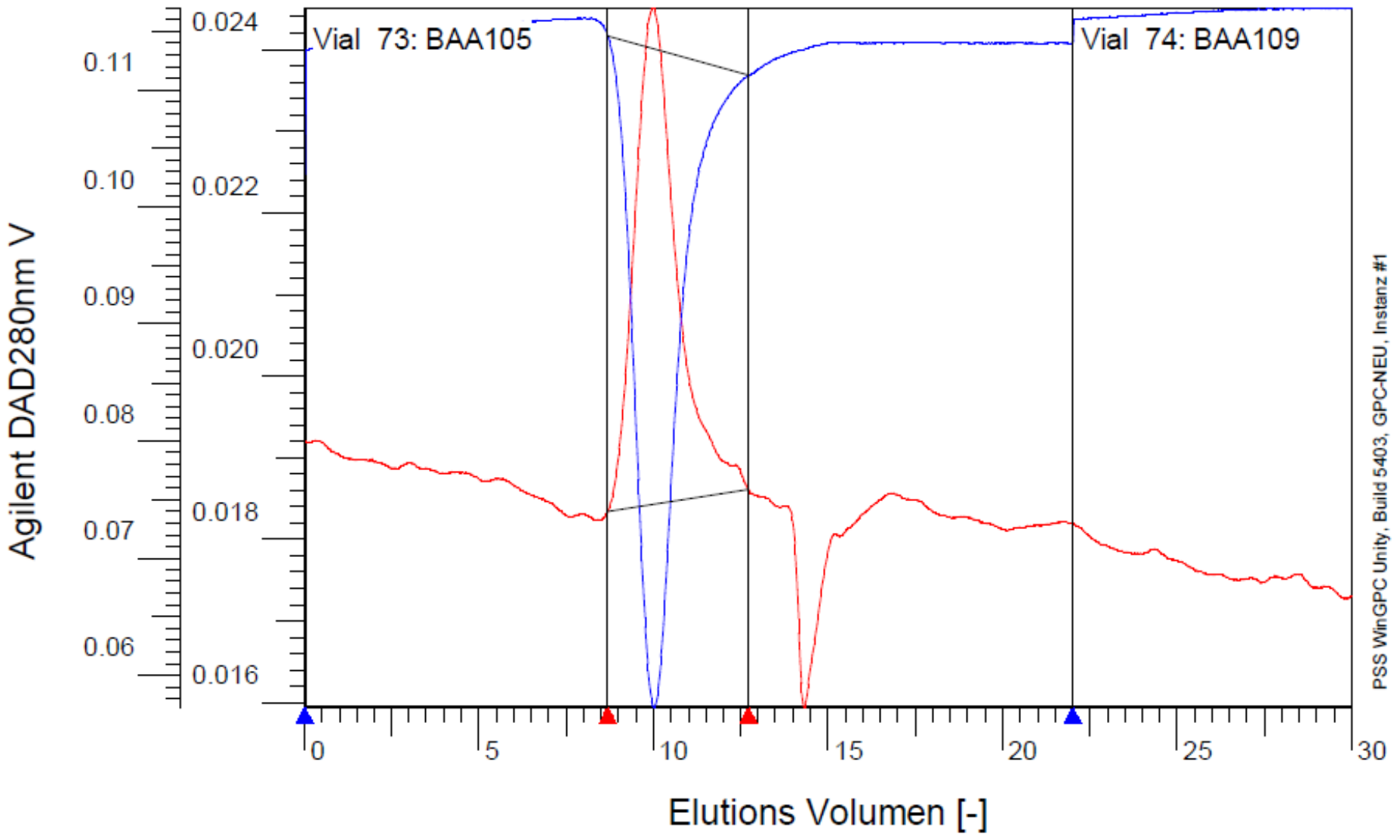

**Probe :**	Vial 73: BAA105	**Methode :**	C:\pss_GPC\Methoden\Methoden Agilent 1100\Water_
**Basislinie von :**	8.696 ml	**bis :**	12.726 ml
**Integration von :**	8.696 ml	**bis :**	12.726 ml
**Kalibration :**	15122023_NovemaMax_LinM_water_KCl_FormBaseIt Pullulan_A-RI	**Eluent :**	Water+15%ACN+NaNO3
**MHK - A (Kal.):**	0.000E+0	**MHK - K (Kal.):**	1.000E+0 ml/g
**Int.Stand.-K :**	12.040 ml	**Int.Stand.-M :**	12.040 ml
**Pumpe :**	Agilent 1100	**Flußrate :**	1.000 ml/min
**Konzentration :**	1.000 g/l	**Injektvolumen :**	50.000 ul
**Säule 1 :**	PSS Suprema analytical 1 000A	**Temperatur :**	30.000 C
**Detektor 1 :**	Shodex RI71	**Versatz :**	0.067 ml
**Detektor 2 :**	Agilent DAD 280	**Versatz :**	0.000 ml
**Operateur :**	Admin	**Messintervall :**	1.000 sec

**Projekt :**	C:\pss_GPC\GPC-Daten\2024\February.LDX	**Kostenstelle :**	
**Datum :**	Dienstag 08/11/26 12:57:16	**Zeichen :**	

Created with novaPDF Printer (www.novaPDF.com). Please register to remove this message.

GPC data S640

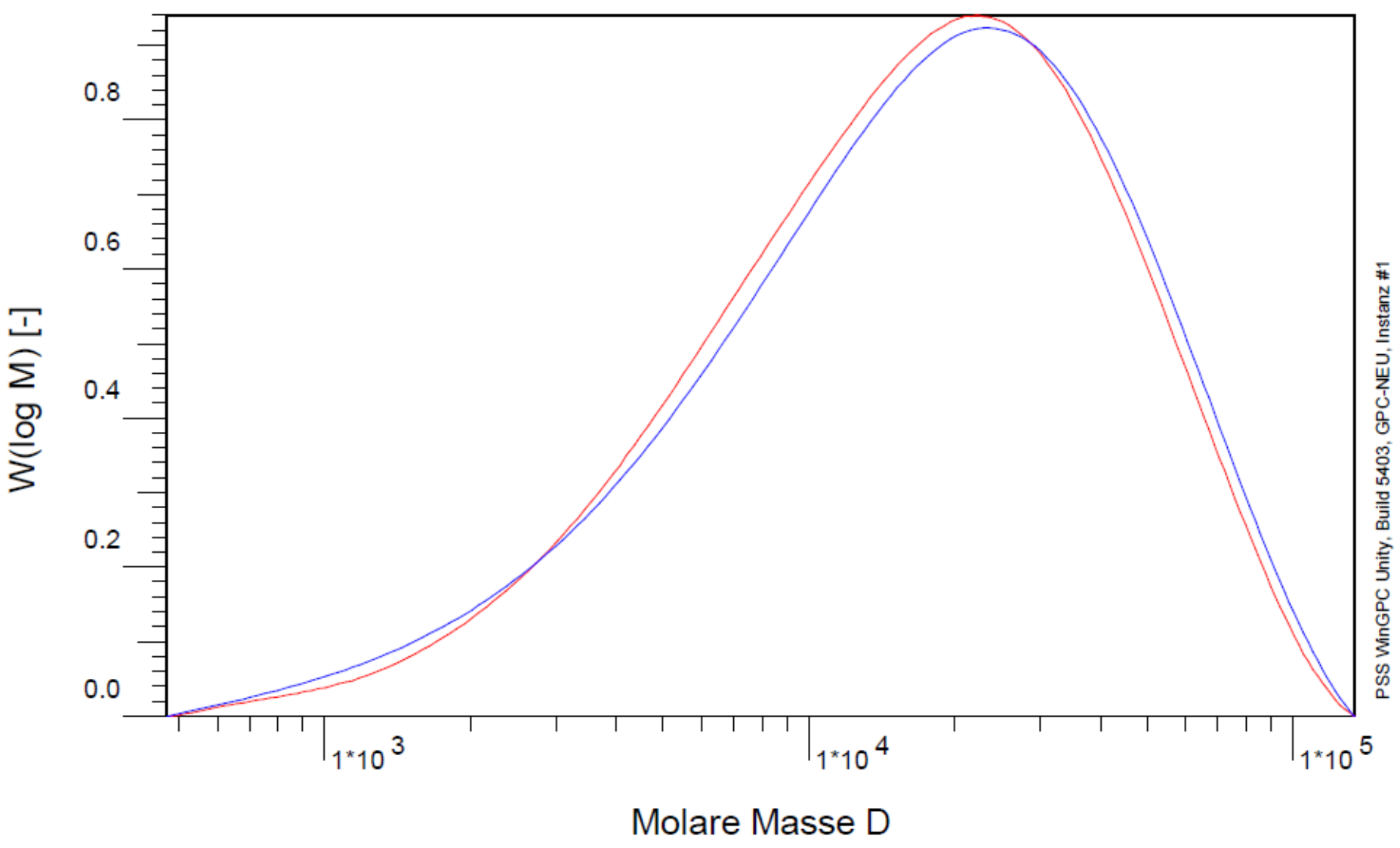

**Probe :**	Vial 75: BAA113		
**Integration von :**	Sonntag 02/28/93 13:05:20		8.712 ml
**Integration bis :**	Sonntag 02/28/93 13:08:27		11.838 ml
**Kalibration :**	15122023_NovemaMax_LinM_water_KCl_FormBased_Pullulan_A-RID.CAL	**Eluent :**	Wasser+15%ACN+NaNO3
**MHK - A (Kal.):**	0.000E+0	**MHK - K (Kal.):**	1.000E+0 ml/g
**Int.Stand.-K :**	12.040 ml	**Int.Stand.-M :**	12.040 ml
**Pumpe :**	Agilent 1100	**Flußrate :**	1.000 ml/min
**Konzentration :**	1.000 g/l	**Injektvolumen :**	50.000 ul
**Säule 1 :**	PSS Suprema analytical 1 000A	**Temperatur :**	30.000 C
**Detektor 1 :**	Shodex RI71	**Versatz :**	0.067 ml
**Detektor 2 :**	Agilent DAD 280	**Versatz :**	0.000 ml
**Operateur :**	Admin	**Messintervall :**	1.000 sec

	**Shodex RI71**	**Agilent DAD 280**	
**Mn :**	9.1311e3	8.8567e3	g/mol
**Mw :**	2.3502e4	2.4538e4	g/mol
**Mz :**	4.1322e4	4.3442e4	g/mol
**Mv :**	0.000000	0.000000	g/mol
**D :**	2.5738e0	2.7706e0	
**[n]:**	0.000000	0.000000	ml/g
**Vp :**	9.9162e0	9.8998e0	ml
**Mp :**	2.0280e4	2.0910e4	g/mol
**FI :**	1.358e-2	2.008e-1	ml*V
**< 470**	0.00	0.00	
**w% :**	100.00	100.00	
**> 133894**	0.00	0.00	

**Projekt :**	C:\pss_GPC\GPC-Daten\2024\February.LDX	**Kostenstelle :**	
**Datum :**	Dienstag 08/11/26 13:01:01	**Zeichen :**	

Created with novaPDF Printer (www.novaPDF.com). Please register to remove this message.

GPC data S741

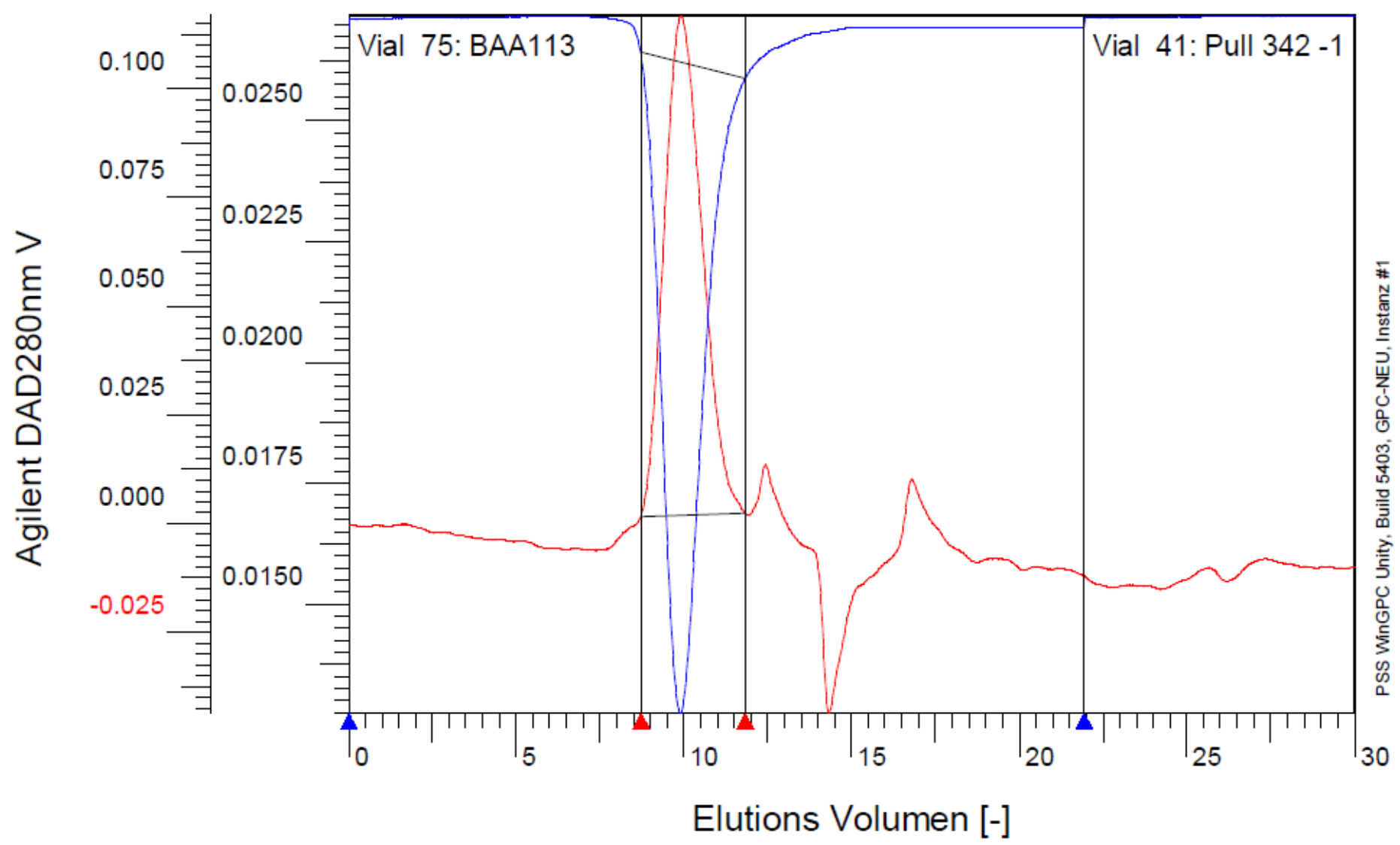

**Probe :**	Vial 75: BAA113	**Methode :**	C:\pss_GPC\Methoden\Methoden Agilent 1100\Water_
**Basislinie von :**	8.712 ml	**bis :**	11.838 ml
**Integration von :**	8.712 ml	**bis :**	11.838 ml
**Kalibration :**	15122023_NovemaMax_LinM_water_KCl_Form	**Eluent :**	Water+15%ACN+NaNO3
**MHK - A (Kal.):**	0.000E+0	**MHK - K (Kal.):**	1.000E+0 ml/g
**Int.Stand.-K :**	12.040 ml	**Int.Stand.-M :**	12.040 ml
**Pumpe :**	Agilent 1100	**Flußrate :**	1.000 ml/min
**Konzentration :**	1.000 g/l	**Injektvolumen :**	50.000 ul
**Säule 1 :**	PSS Suprema analytical 1 000A	**Temperatur :**	30.000 C
**Detektor 1 :**	Shodex RI71	**Versatz :**	0.067 ml
**Detektor 2 :**	Agilent DAD 280	**Versatz :**	0.000 ml
**Operateur :**	Admin	**Messintervall :**	1.000 sec

**Projekt :**	C:\pss_GPC\GPC-Daten\2024\February.LDX	**Kostenstelle :**	
**Datum :**	Dienstag 08/11/26 13:00:40	**Zeichen :**	

Created with novaPDF Printer (www.novaPDF.com). Please register to remove this message.

GPC data S842

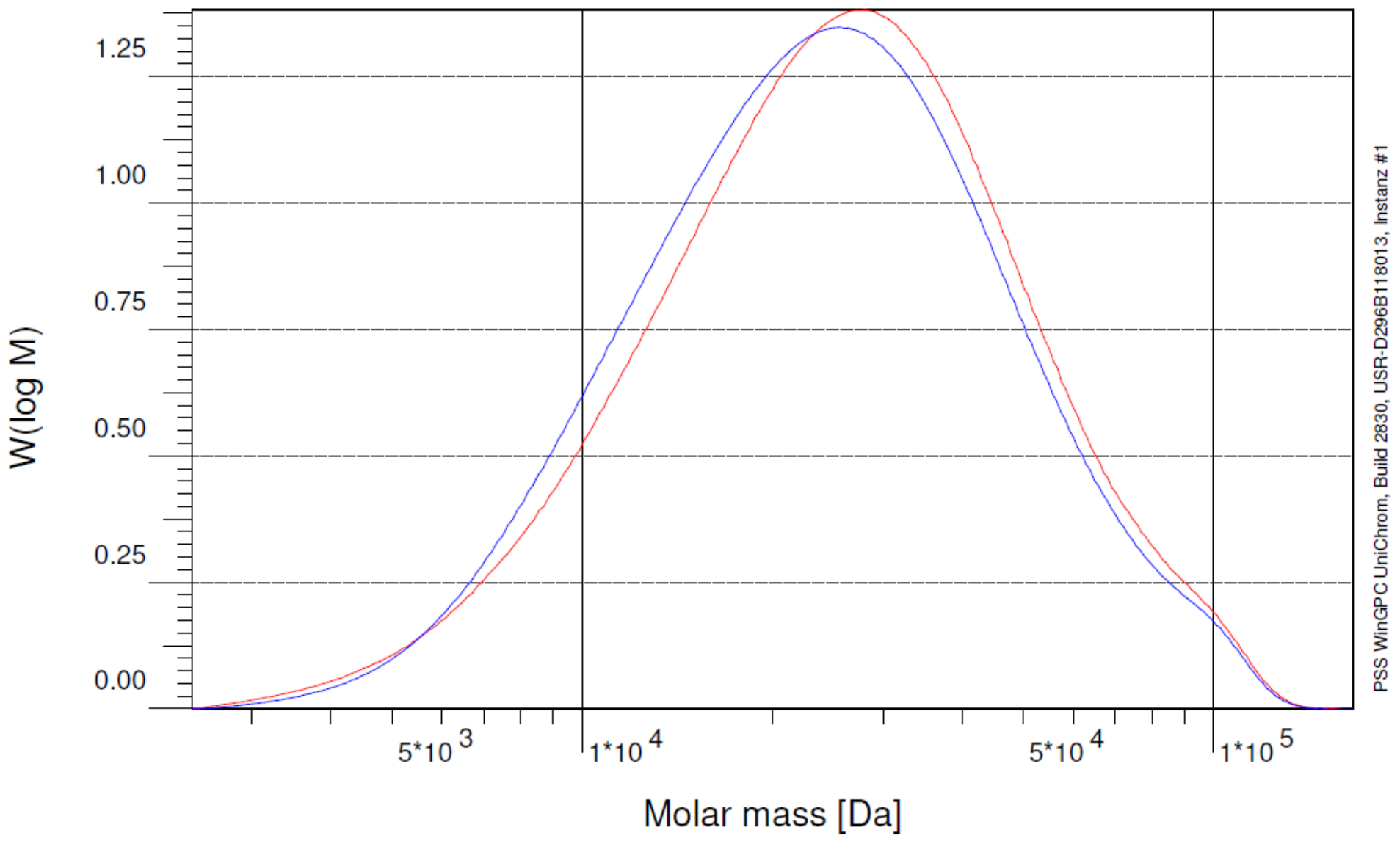

**Probe :**	Vial 13: FRJ100 - 1		
**Integration von :**	Montag 15.04.24 20:20:37		6.532 ml
**Integration bis :**	Montag 15.04.24 20:24:44		10.651 ml
**Kalibration :**	20022024GRAM 100A DMAcPMMA_LiBr.CAL	**Eluent :**	DMAc + LiBr 0.1
**MHK - A (Kal.):**	0.000E+0	**MHK - K (Kal.):**	1.000E+0 ml/g
**Int.Stand.-K :**	12.160 ml	**Int.Stand.-M :**	12.179 ml
**Pumpe :**	PSS SECcurity	**Flußrate :**	1.000 ml/min
**Konzentration :**	0.000 g/l	**Injektvolumen :**	20.000 ul
**Säule 1 :**	PSS GRAM 100A 2018	**Temperatur :**	60.000 °C
**Detektor 1 :**	I1: IsoPump 1, Pressure	**Versatz :**	0.000 ml
**Detektor 2 :**	I1: RID 1, RI Signal	**Versatz :**	0.170 ml
**Detektor 3 :**	I1: VWD 1, Signal A	**Versatz :**	0.000 ml
**Operateur :**	usr	**Messintervall :**	1.000 sec

	**I1: RID 1, RI Signal**	**I1: VWD 1, Signal A**	
**Mn :**	1.9564e4	1.8866e4	g/mol
**Mw :**	3.0737e4	2.9312e4	g/mol
**Mz :**	4.4648e4	4.3138e4	g/mol
**Mv :**	0.000000	0.000000	g/mol
**D :**	1.5711e0	1.5537e0	
**[n]:**	0.000000	0.000000	ml/g
**Vp :**	8.5001e0	8.5866e0	ml
**Mp :**	2.6860e4	2.4711e4	g/mol
**FI :**	3.4714e3	1.2488e2	ml*V
**< 2407**	0.00	0.00	
**w% :**	100.00	100.00	
**> 166705**	0.00	0.00	

**Projekt :**	C:\GPC-Daten\2024\April.LDX	**Kostenstelle :**	
**Datum :**	Dienstag 30.04.24 13:07:21	**Zeichen :**	

GPC data S943

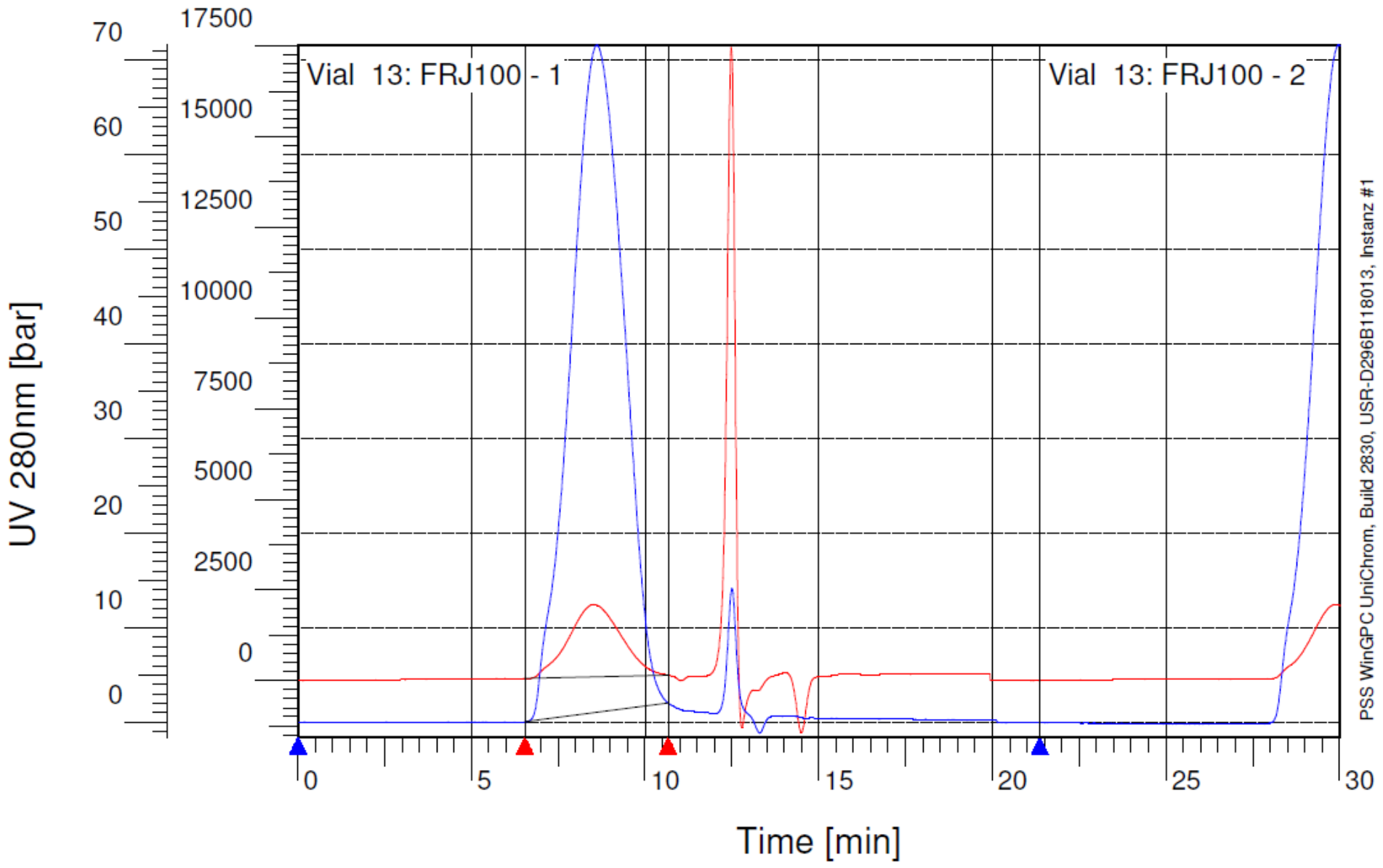

**Probe :**	Vial 13: FRJ100 - 1	**Methode :**	C:\wingpc_8#1\Methoden 2023\GRAM_100A_DM
**Basislinie von :**	6.542 ml	**bis :**	10.668 ml
**Integration von :**	6.532 ml	**bis :**	10.651 ml
**Kalibration :**	20022024GRAM 100A DMAcPMMA_LiBr.CAL	**Eluent :**	DMAc + LiBr 0.1
**MHK - A (Kal.):**	0.000E+0	**MHK - K (Kal.):**	1.000E+0 ml/g
**Int.Stand.-K :**	12.160 ml	**Int.Stand.-M :**	12.179 ml
**Pumpe :**	PSS SECcurity	**Flußrate :**	1.000 ml/min
**Konzentration :**	0.000 g/l	**Injektvolumen :**	20.000 ul
**Säule 1 :**	PSS GRAM 100A 2018	**Temperatur :**	60.000 °C
**Detektor 1 :**	I1: IsoPump 1, Pressure	**Versatz :**	0.000 ml
**Detektor 2 :**	I1: RID 1, RI Signal	**Versatz :**	0.170 ml
**Detektor 3 :**	I1: VWD 1, Signal A	**Versatz :**	0.000 ml
**Operateur :**	usr	**Messintervall :**	1.000 sec

**Projekt :**	C:\GPC-Daten\2024\April.LDX	**Kostenstelle :**	
**Datum :**	Dienstag 11.08.26 13:14:03	**Zeichen :**	

GPC data S1044

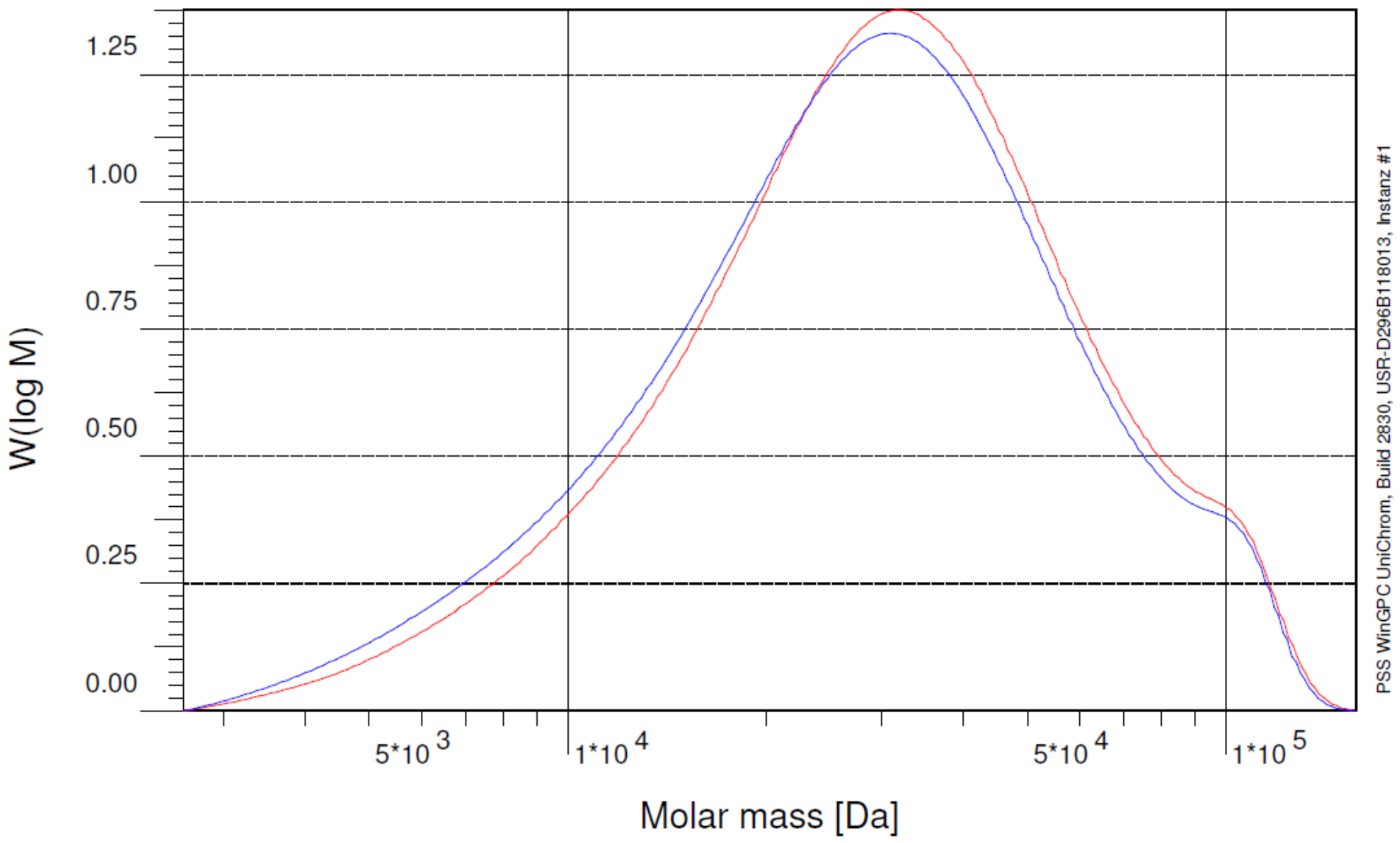

**Probe :**	Vial 14: FRJ101 - 1		
**Integration von :**	Montag 15.04.24 21:03:26		6.591 ml
**Integration bis :**	Montag 15.04.24 21:07:26		10.592 ml
**Kalibration :**	20022024GRAM 100A DMAcPMMA_LiBr.CAL	**Eluent :**	DMAc + LiBr 0.1
**MHK - A (Kal.):**	0.000E+0	**MHK - K (Kal.):**	1.000E+0 ml/g
**Int.Stand.-K :**	12.160 ml	**Int.Stand.-M :**	12.179 ml
**Pumpe :**	PSS SECcurity	**Flußrate :**	1.000 ml/min
**Konzentration :**	0.000 g/l	**Injektvolumen :**	20.000 ul
**Säule 1 :**	PSS GRAM 100A 2018	**Temperatur :**	60.000 °C
**Detektor 1 :**	I1: IsoPump 1, Pressure	**Versatz :**	0.000 ml
**Detektor 2 :**	I1: RID 1, RI Signal	**Versatz :**	0.170 ml
**Detektor 3 :**	I1: VWD 1, Signal A	**Versatz :**	0.000 ml
**Operateur :**	usr	**Messintervall :**	1.000 sec

	I1: RID 1, RI Signal	I1: VWD 1, Signal A	
**Mn :**	2.2221e4	2.0708e4	g/mol
**Mw :**	3.6598e4	3.5132e4	g/mol
**Mz :**	5.3709e4	5.2568e4	g/mol
**Mv :**	0.000000	0.000000	g/mol
**D :**	1.6470e0	1.6965e0	
**[n]:**	0.000000	0.000000	ml/g
**Vp :**	8.3448e0	8.3814e0	ml
**Mp :**	3.1145e4	3.0082e4	g/mol
**FI :**	2.9975e3	1.3956e2	ml*V
**< 2605**	0.00	0.00	
**w% :**	100.00	100.00	
**> 157878**	0.00	0.00	

**Projekt :**	C:\GPC-Daten\2024\April.LDX	**Kostenstelle :**	
**Datum :**	Dienstag 30.04.24 13:08:23	**Zeichen :**	

GPC data S1145

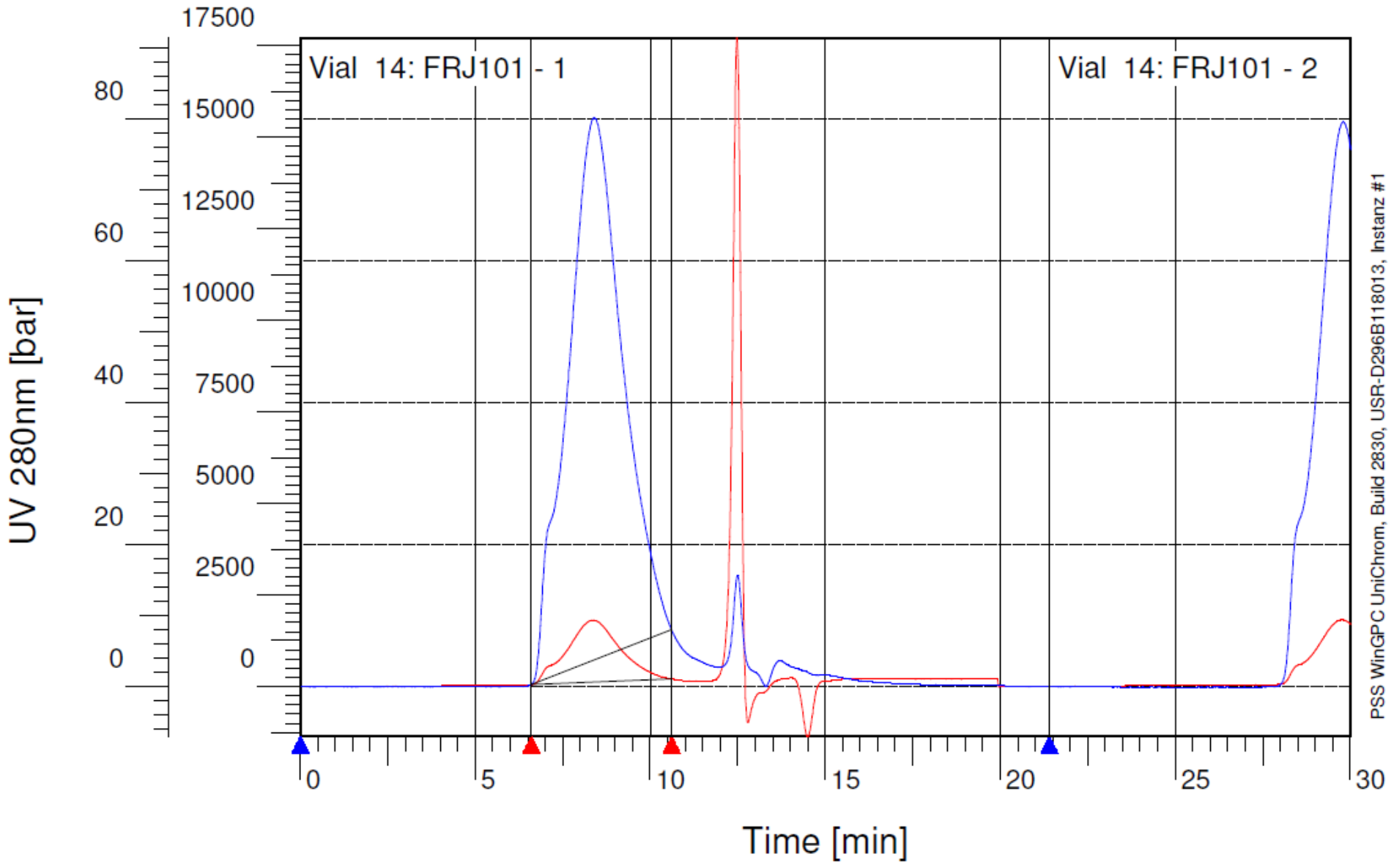

**Probe :**	Vial 14: FRJ101 - 1	**Methode :**	C:\wingpc_8#1\Methoden 2023\GRAM_100A_DM
**Basislinie von :**	6.601 ml	**bis :**	10.609 ml
**Integration von :**	6.591 ml	**bis :**	10.592 ml
**Kalibration :**	20022024GRAM 100A DMAcPMMA_LiBr.CAL	**Eluent :**	DMAc + LiBr 0.1
**MHK - A (Kal.):**	0.000E+0	**MHK - K (Kal.):**	1.000E+0 ml/g
**Int.Stand.-K :**	12.160 ml	**Int.Stand.-M :**	12.179 ml
**Pumpe :**	PSS SECcurity	**Flußrate :**	1.000 ml/min
**Konzentration :**	0.000 g/l	**Injektvolumen :**	20.000 ul
**Säule 1 :**	PSS GRAM 100A 2018	**Temperatur :**	60.000 °C
**Detektor 1 :**	I1: IsoPump 1, Pressure	**Versatz :**	0.000 ml
**Detektor 2 :**	I1: RID 1, RI Signal	**Versatz :**	0.170 ml
**Detektor 3 :**	I1: VWD 1, Signal A	**Versatz :**	0.000 ml
**Operateur :**	usr	**Messintervall :**	1.000 sec

**Projekt :**	C:\GPC-Daten\2024\April.LDX	**Kostenstelle :**	
**Datum :**	Dienstag 11.08.26 13:16:04	**Zeichen :**	

GPC data S1246

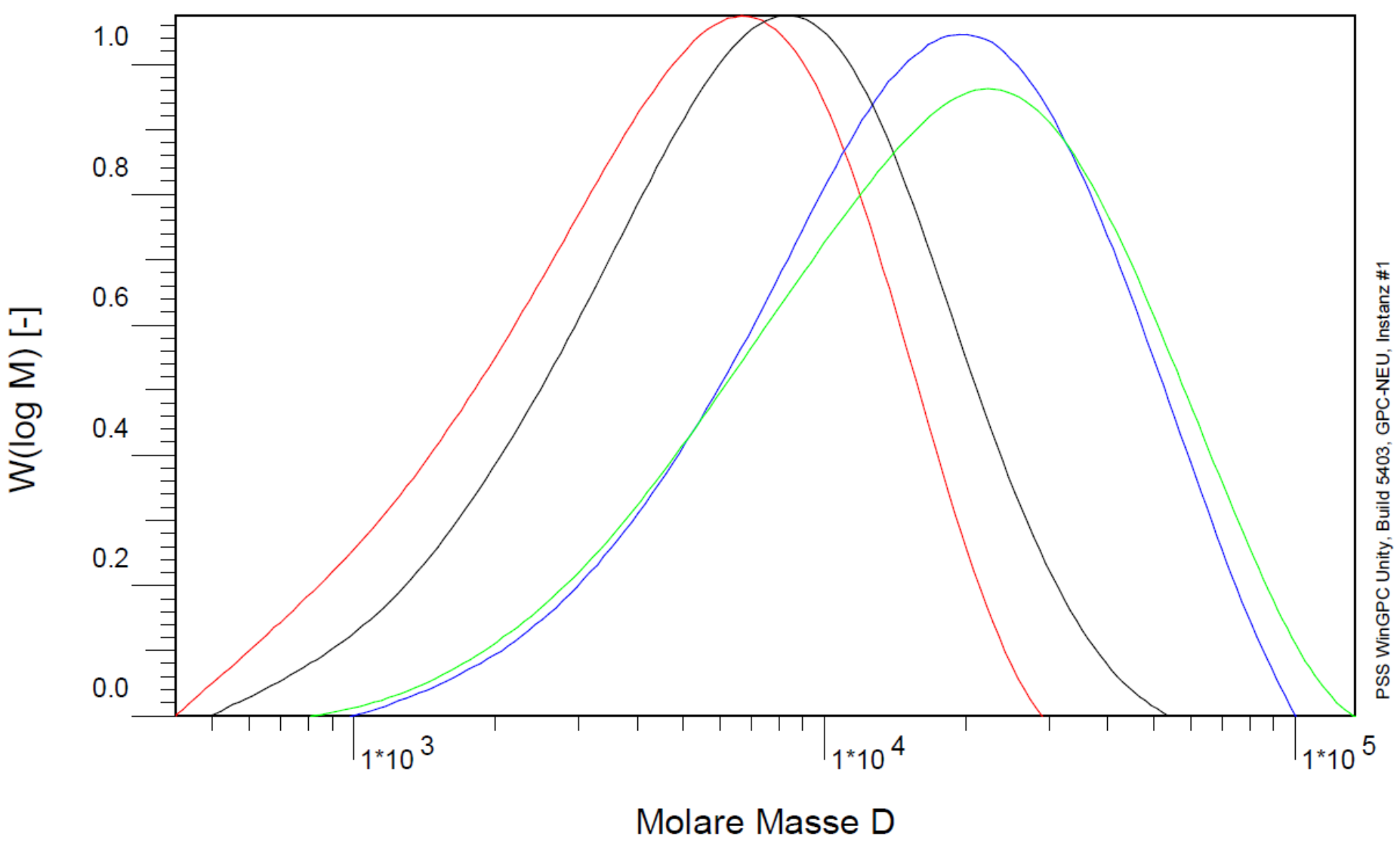

Kurve 1 : Vial 72: BAA095
Kurve 2 : Vial 73: BAA105
Kurve 3 : Vial 74: BAA109
Kurve 4 : Vial 75: BAA113

	Shodex RI71	Shodex RI71	Shodex RI71	Shodex RI71	
**Mn :**	3.2667e3	1.0615e4	4.5850e3	1.0374e4	g/mol
**Mw :**	6.4578e3	2.1623e4	9.1916e3	2.3953e4	g/mol
**Mz :**	1.0103e4	3.4847e4	1.5195e4	4.1325e4	g/mol
**Mv :**	0.000000	0.000000	0.000000	0.000000	g/mol
**D :**	1.9769e0	2.0370e0	2.0047e0	2.3090e0	
**[n]:**	0.000000	0.000000	0.000000	0.000000	ml/g
**Vp :**	1.0487e1	9.9723e0	1.0394e1	9.9162e0	ml
**Mp :**	6.6641e3	1.8242e4	8.0186e3	2.0280e4	g/mol
**FI :**	8.030e-3	6.362e-3	1.211e-2	1.310e-2	ml*V
**< 415**	0.00	0.00	0.00	0.00	
**w% :**	100.00	100.00	100.00	100.00	
**> 133894**	0.00	0.00	0.00	0.00	

Projekt : C:\pss_GPC\GPC-Daten\2024\February.LDX **Kostenstelle :**
Datum : Mittwoch 02/28/24 14:30:21 **Zeichen :**

Created with novaPDF Printer (www.novaPDF.com). Please register to remove this message.

GPC data S1347

S5 | Supplementary Tables

TABLE S1 | Bacteria used in this study with some of their noticeable characteristics.

Species	Strain	Origin, source, reference
S. aureus (SA)	Newman	Clinical (human infection) https://www.ncbi.nlm.nih.gov/nuccore/AP009351.1
	N315	Clinical (nosocomial), MRSA https://www.ncbi.nlm.nih.gov/nuccore/47118324
E. coli (EC)	DH5α	Laboratory https://www.ncbi.nlm.nih.gov/nuccore/NZ_CP025520.1
P. aeruginosa (PA)	PA19660	Laboratory, mucoid https://www.atcc.org/products/19660
	CH2678	Clinical https://bactome.helmholtz-hzi.de/
	F1758	Clinical https://bactome.helmholtz-hzi.de/

[a] MRSA, methicillin resistant *S. aureus*.

TABLE S2 | Composition and characteristics of aPDT-polymers.

aPDT-polymers	17	14	13	16	18	15	19
[Ru] (Mol%)	1.3	0.6	0.5	0.5	2.0	1.0	1.0
R[a]	R4	R1	R1	R3	R2	R3	R3
HEAAm (Mol%)	76.7	95.0	99.5	77.5	25.0	97.0	77.0
Surfactant (Mol%)	2.0	4.4	0.0	2.0	2.0	0.0	2.0
AA (Mol%)	20	0	0	20	71	0	20
Synthesis route[b]	PPM	FRP	FRP	PPM	PPM	FRP	FRP
Mn (KDa)	6.5	10.6	8.0	10.0	12.0	19.0	22.0
Charge (mV)[c]	Nd	+22.0	+27.8	-49.5	-28.1	+14.7	-16.9
Size (nm)[c]	Nd	478	535	220	1009	348	58
PDI[c]	Nd	0.49	0.54	0.46	0.75	0.46	0.35
A450[d]	0.135	0.067	0.058	0.071	0.106	0.057	0.070
Fluorescence (RFU)[d]	440	384	176	592	1264	304	392
$^1O_2$ production[d,e]	883	2288	1872	1155	2424	1747	1035
PA MIC (µg/mL)[f]	Nd	7.8	15.6	0.5	>500	15.6	62.5
EC MIC (µg/mL)[f]	Nd	3.9	15.6	>500	>500	15.6	>500
SA MIC (µg/mL)[f]	Nd	15.6	31.3	>500	>500	31.3	>500

[a], see Figure 1A in the main text; [b], PPM or FRP; [c], polydispersity index, for 1 mg/mL solution in water (DLS measurements); [d], for 50 µg/mL solutions in water with 100 µL per well (see Experimental section for details); [e], SOSG fluorescence (in RFU); [f], with reference to Table 1 in the main text. Nd, not determined; RFU, relative fluorescence unit.

S6 | Supplementary Figures

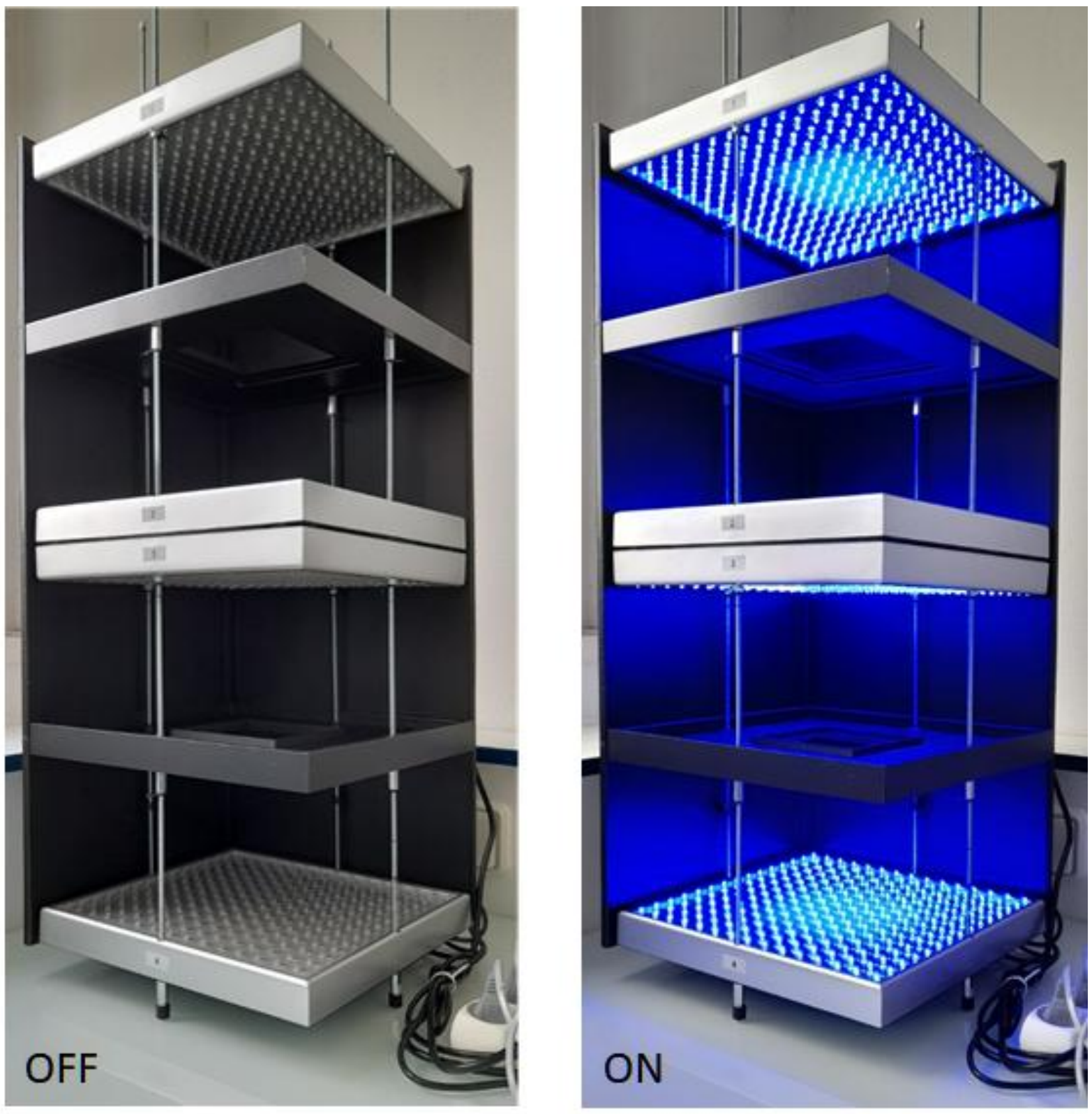

FIGURE S1 | Images of the light setup device used in this study. This is an enhanced version of the device previously reported.[3] It features two illumination stations, each equipped with two opposing LED panels, enabling light exposure for materials of various sizes (e.g., multiwell plates, culture dishes). Light treatments are conducted at room temperature (~22 °C). The setup is shown with the LED panels turned OFF (left) and ON (right). Importantly, temperature monitoring during blue LED irradiation revealed only a modest increase (~5°C, over 30 minutes), indicating that the observed antibacterial effects were predominantly photodynamic in nature rather than photothermal.

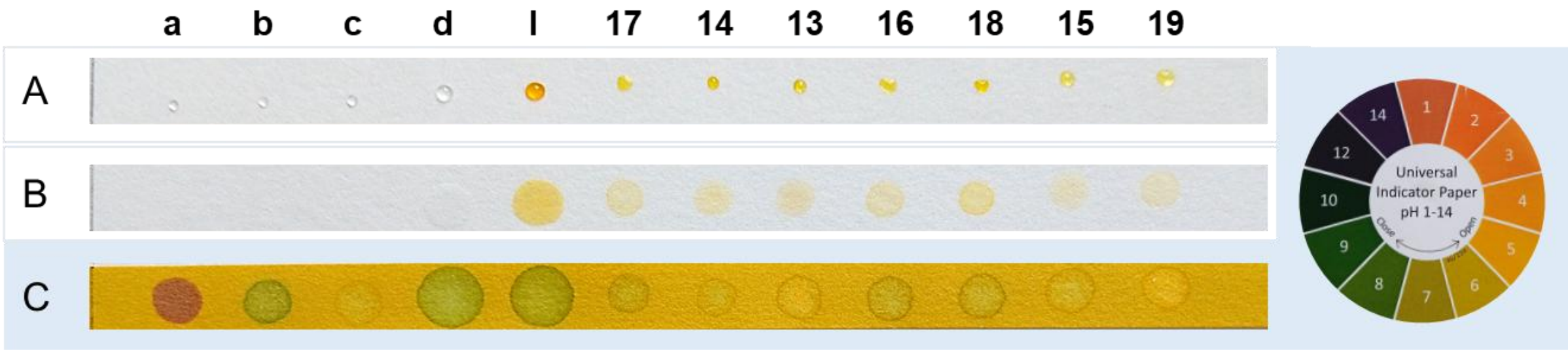

FIGURE S2 | Pictures of the aPDT-polymers at the liquid state in water **(panel A)**, in the dry state **(panel B)**, and applied to a pH paper **(panel C)**. For each condition, a 1 µL droplet was used, corresponding to 5 µg of compound **I** or a given aPDT-polymer. The labels a, b, c, and d correspond to 0.1 N HCl, 0.1 M HEPES, water, and DMSO, respectively. Compound **I** was not in water, but in DMSO.

A) Polymers **II** and **III**: UV-visible absorption and Fluorescence emission spectrum

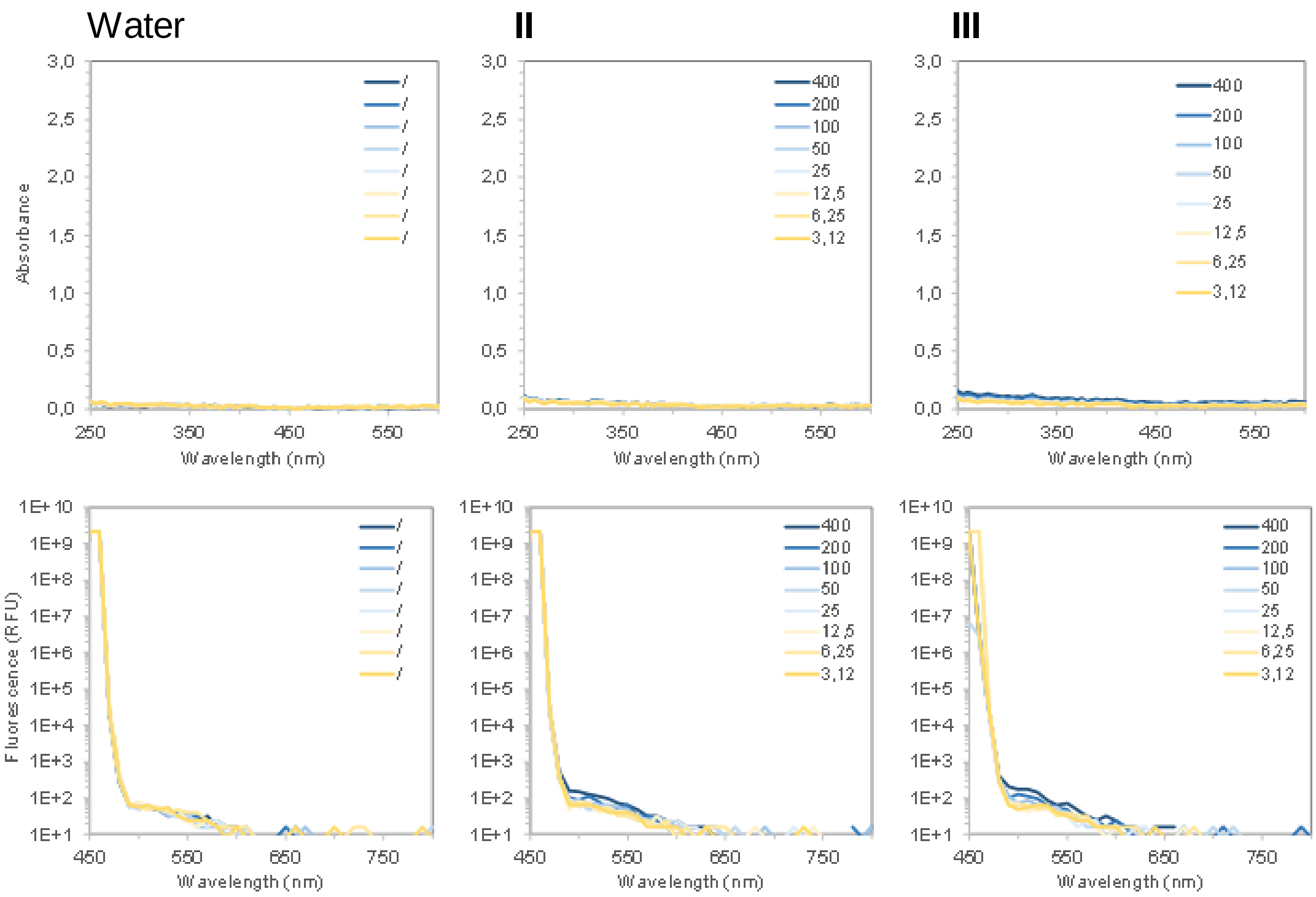

B) Compound **I** and aPDT polymers **13-19**: UV-visible absorption

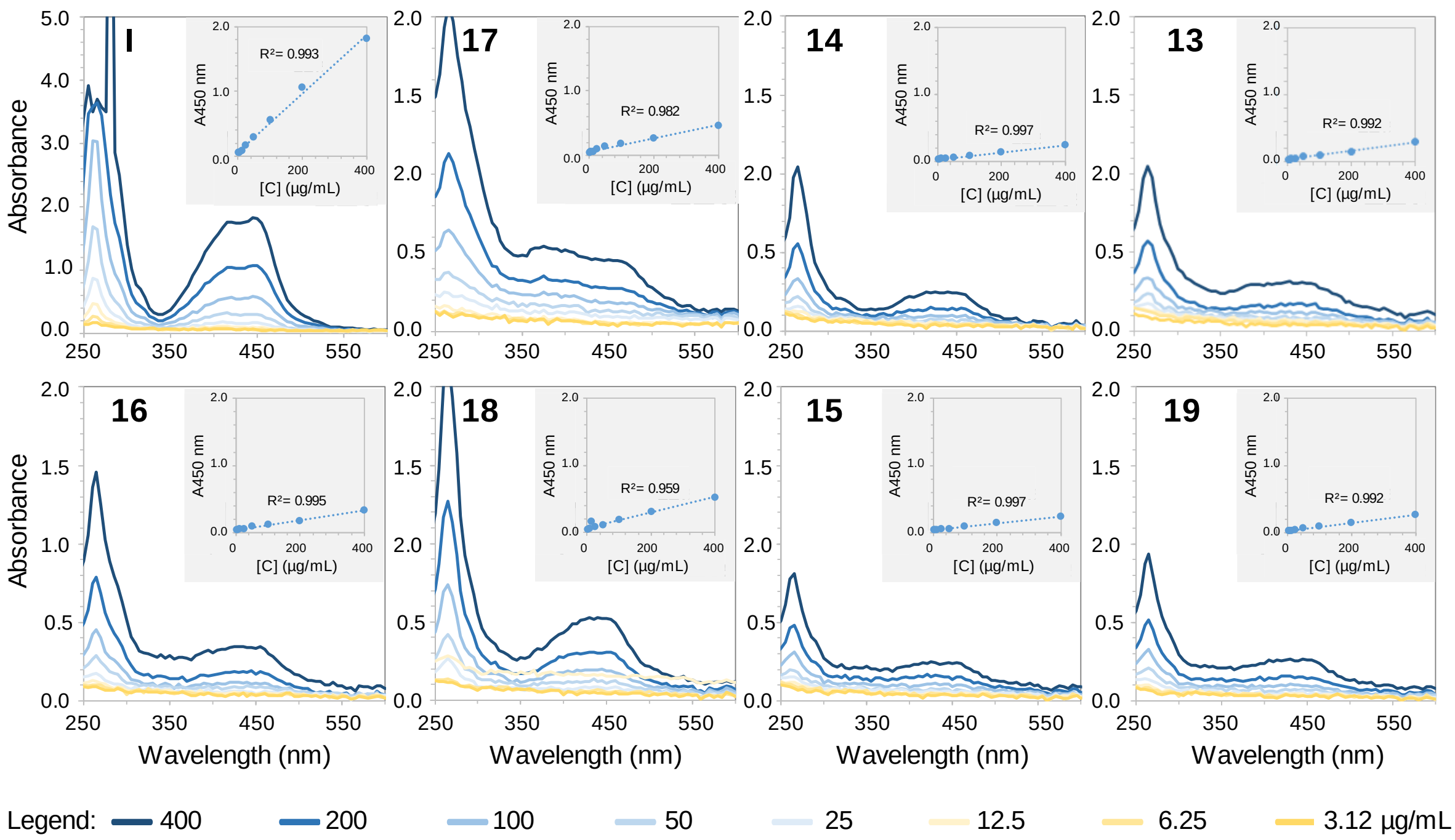

C) Compound **I** and aPDT polymers **13-19**: Fluorescence emission spectrum

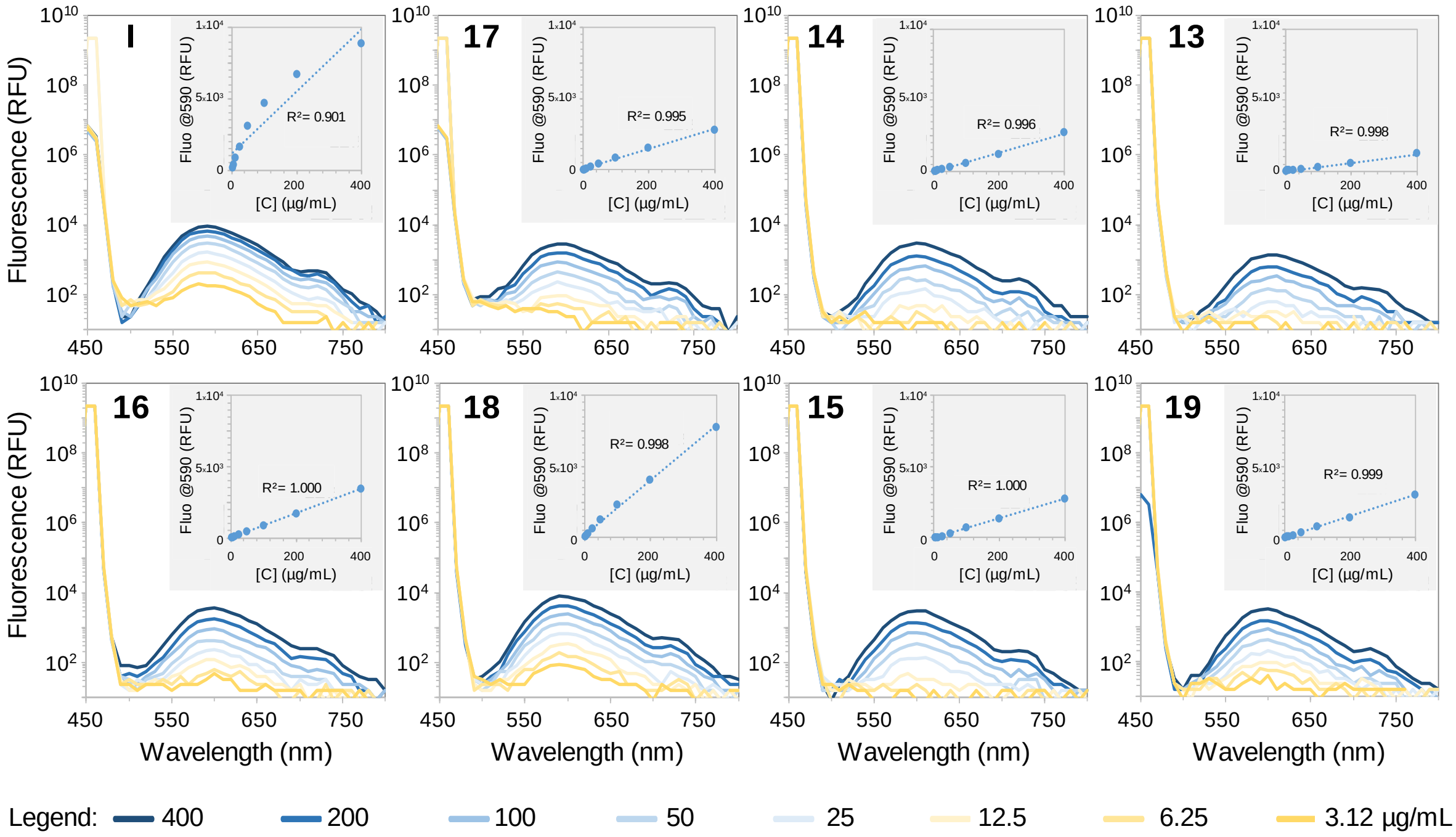

FIGURE S3 | UV-visible absorption and fluorescence emission spectra (upon excitation at 450 nm) of water, polymers **II** and **III** , and aPDT polymers **13-19** recorded across a range of concentrations (400 to 3.12 µg/mL). Measurements were performed in water. The insets show the correlation between the absorbance at 450 nm or the fluorescence at 590 nm, both as a function of the reagent's concentration.

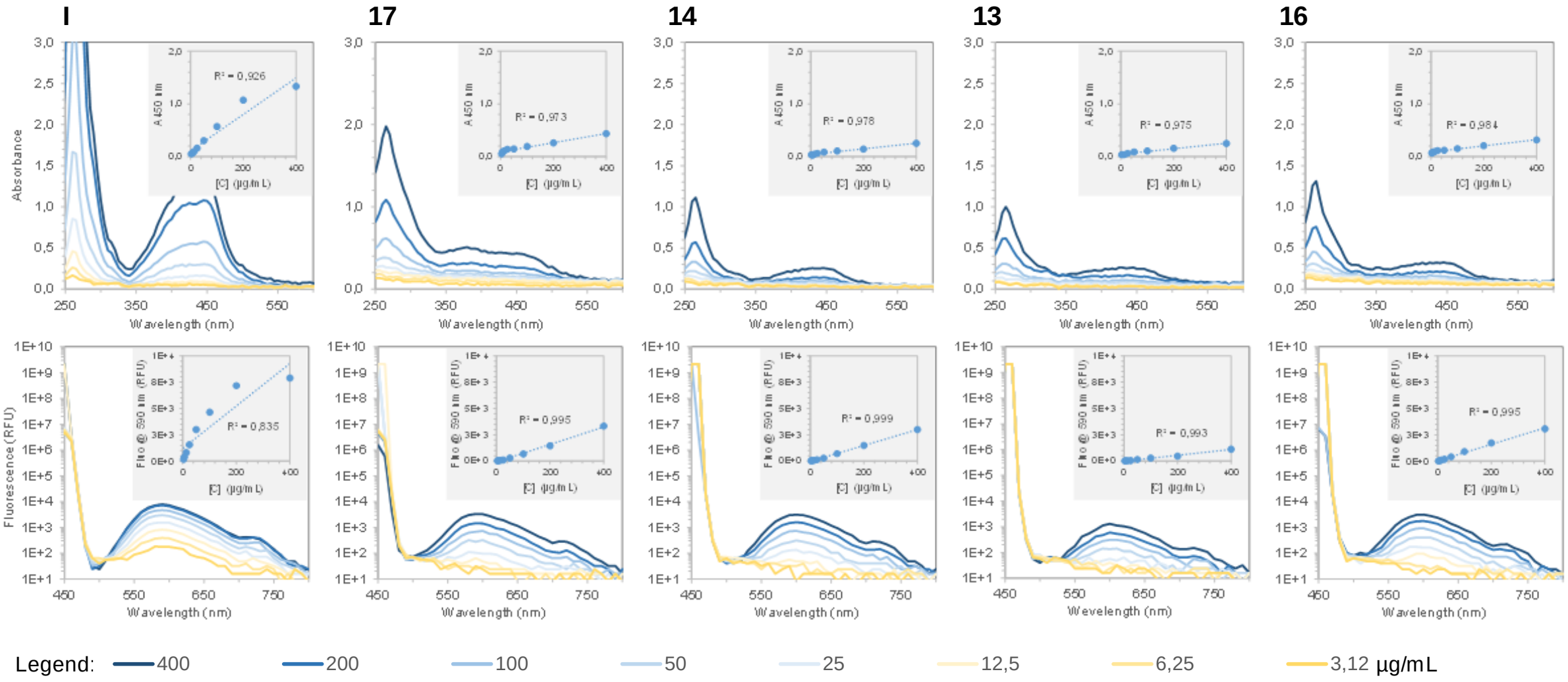

FIGURE S4 | UV-visible absorption and fluorescence emission spectra (upon excitation at 450 nm) of **I** and aPDT-polymers **17**, **14**, **13**, and **16** recorded across a range of concentrations (400 to 3.12 µg/mL) in 0.9% NaCl. The insets illustrate the correlation between absorbance at 450 nm (top) and fluorescence intensity at 590 nm (bottom) as a function of concentration.

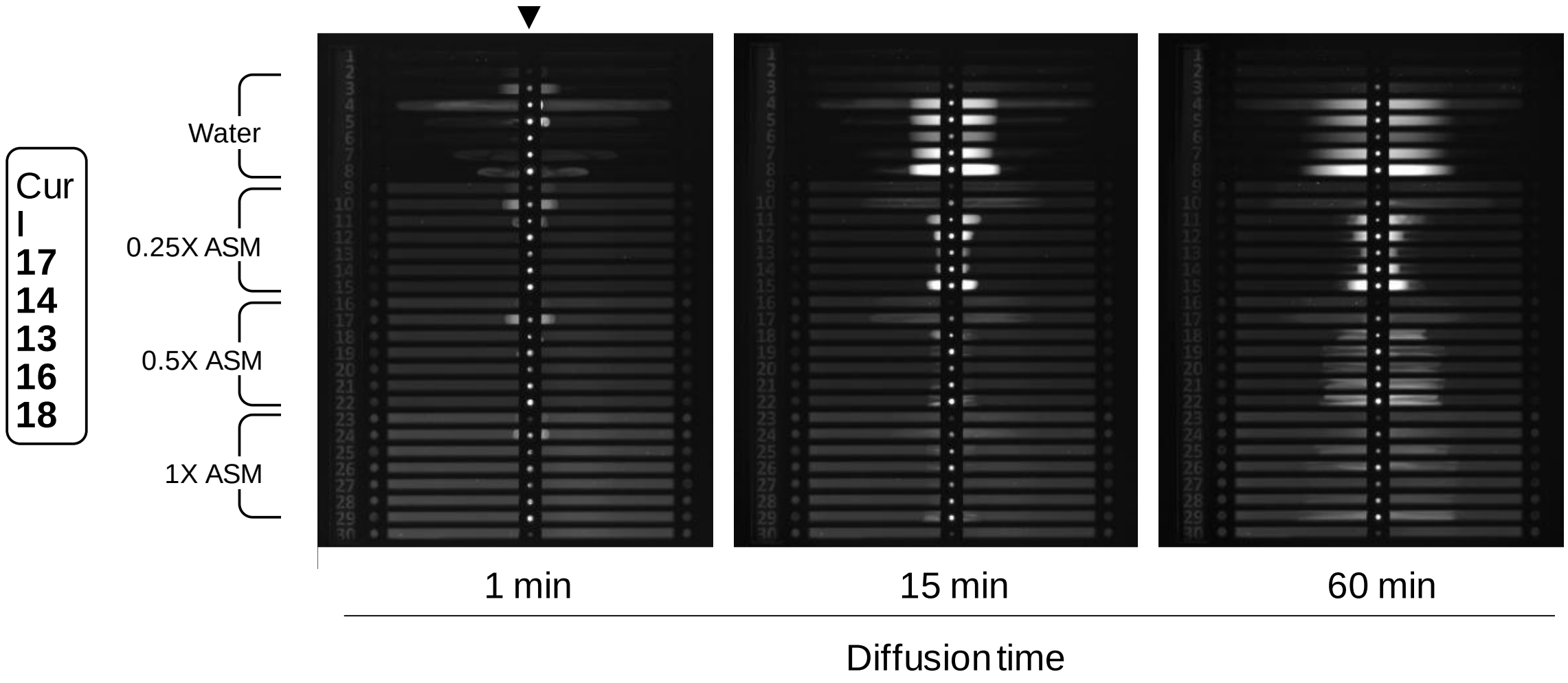

FIGURE S5 | Passive diffusion of compound **I** and PDT-polymers in either water or artificial sputum medium (ASM) at different concentrations. Images were captured under UV exposure at various time points, starting 1 minute after compound deposition (arrowhead). Curcumin (Cur) was used as a non-diffusive control.

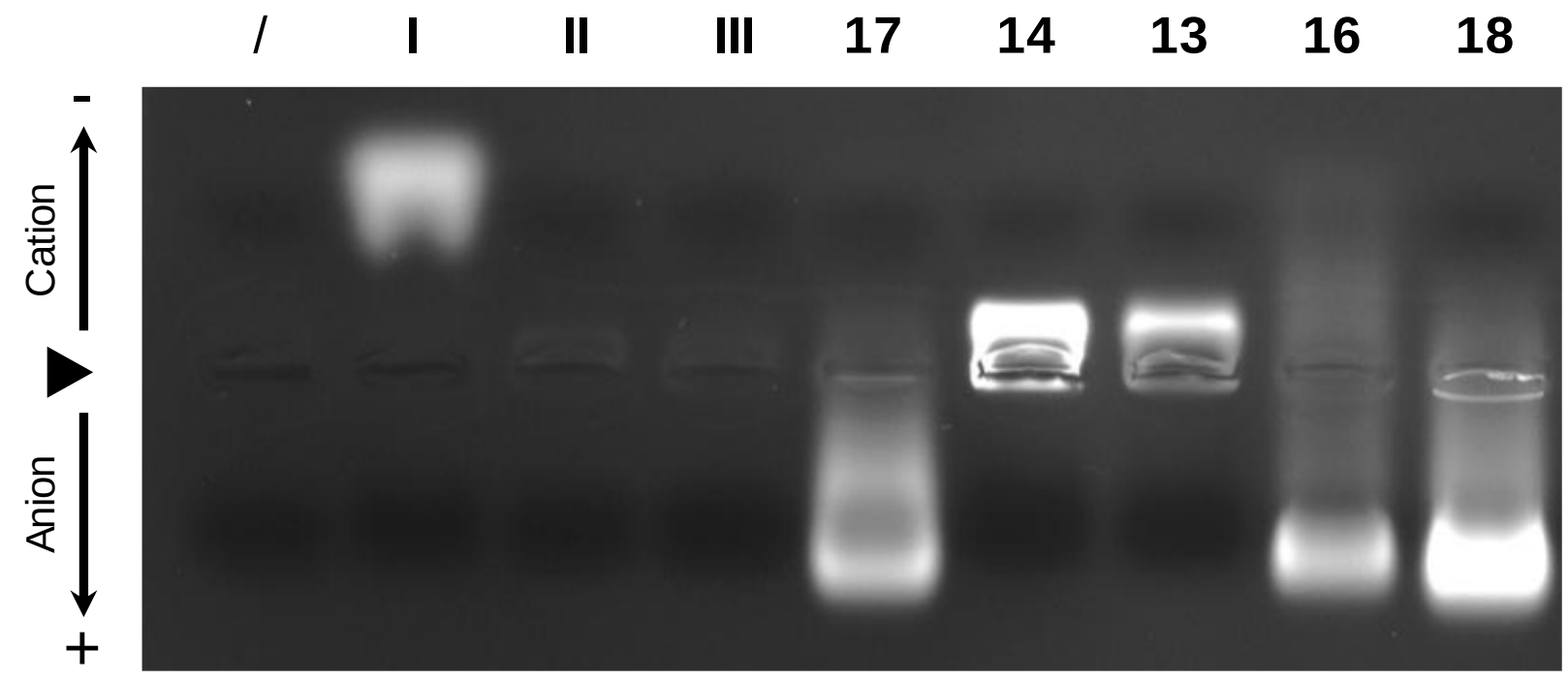

FIGURE S6 | Agarose gel electrophoresis of **I**, polymer backbones lacking Ru moieties, and various aPDT-polymers. Compounds **II** and **III** were synthesized via FRP and PPM strategies without reaction with any PS. The arrowhead marks the location of the sample wells, where 2 µg of **I** and 20 µg of polymers were loaded. Vertical arrows indicate the direction of electrophoretic migration, which varies depending on the net charge of the analytes, positive or negative.

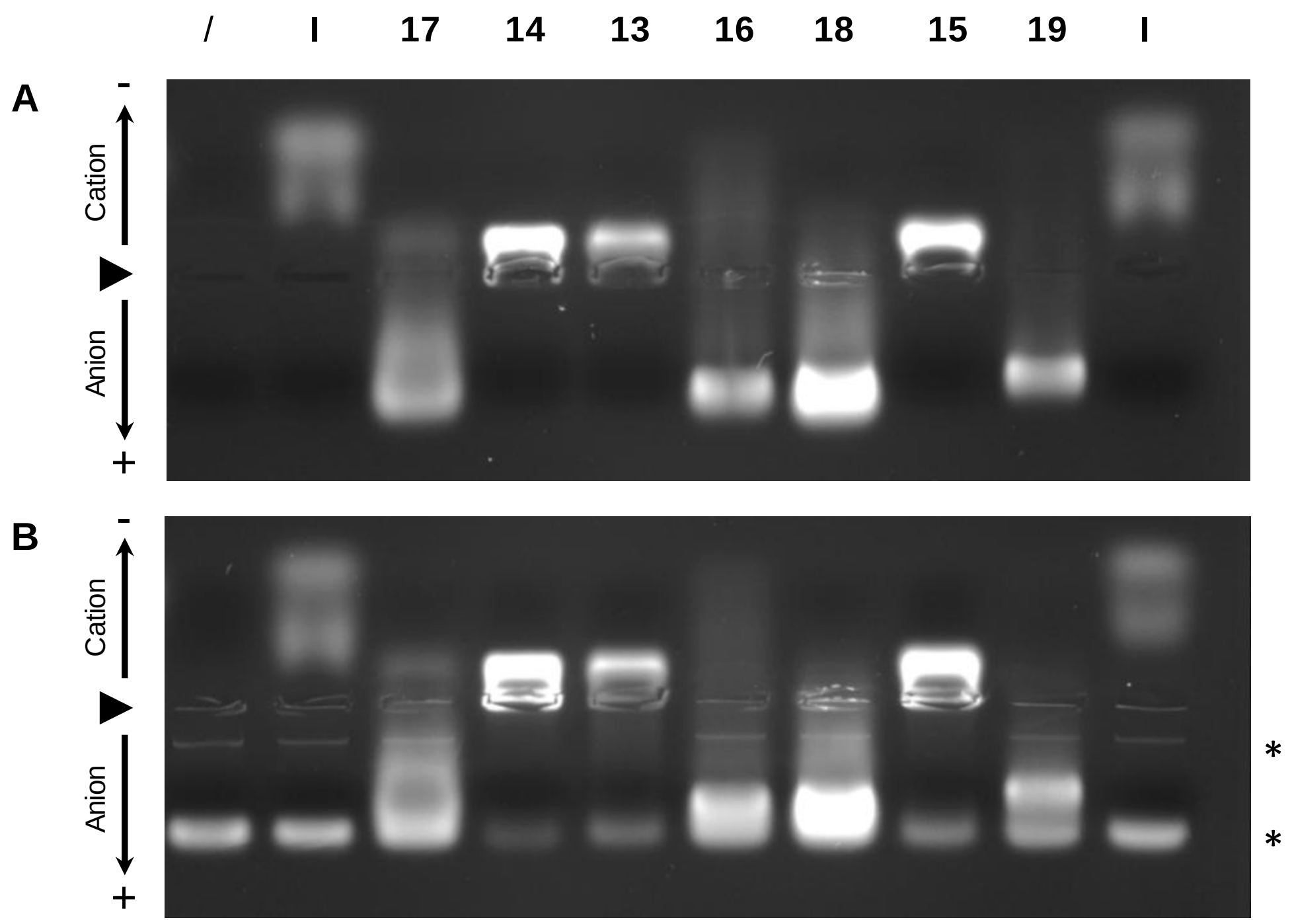

FIGURE S7 | Agarose gel electrophoresis of compound **I** and aPDT-polymers: Comparison of migration profiles for mixtures done in water **(panel A)** versus in artificial sputum medium (ASM, **panel B**). The arrowhead marks the location of the sample wells, where 2 µg of **I** and 20 µg of aPDT-polymers were loaded. Vertical arrows indicate the direction of electrophoretic migration, determined by the net charge of each reagent. The asterisks (*) specify DNA bands originating from ASM, visualized through ethidium bromide staining. The sign / denotes an empty well.

Note: Compared to other lanes, the DNA signal is either absent (upper band) or markedly reduced (lower band) in lanes corresponding to positively charged aPDT-polymers (i.e., **14**, **13**, and **15**). This observation suggests that DNA is complexed within these polymers via electrostatic interactions, resulting in the formation of so-called polyplexes.

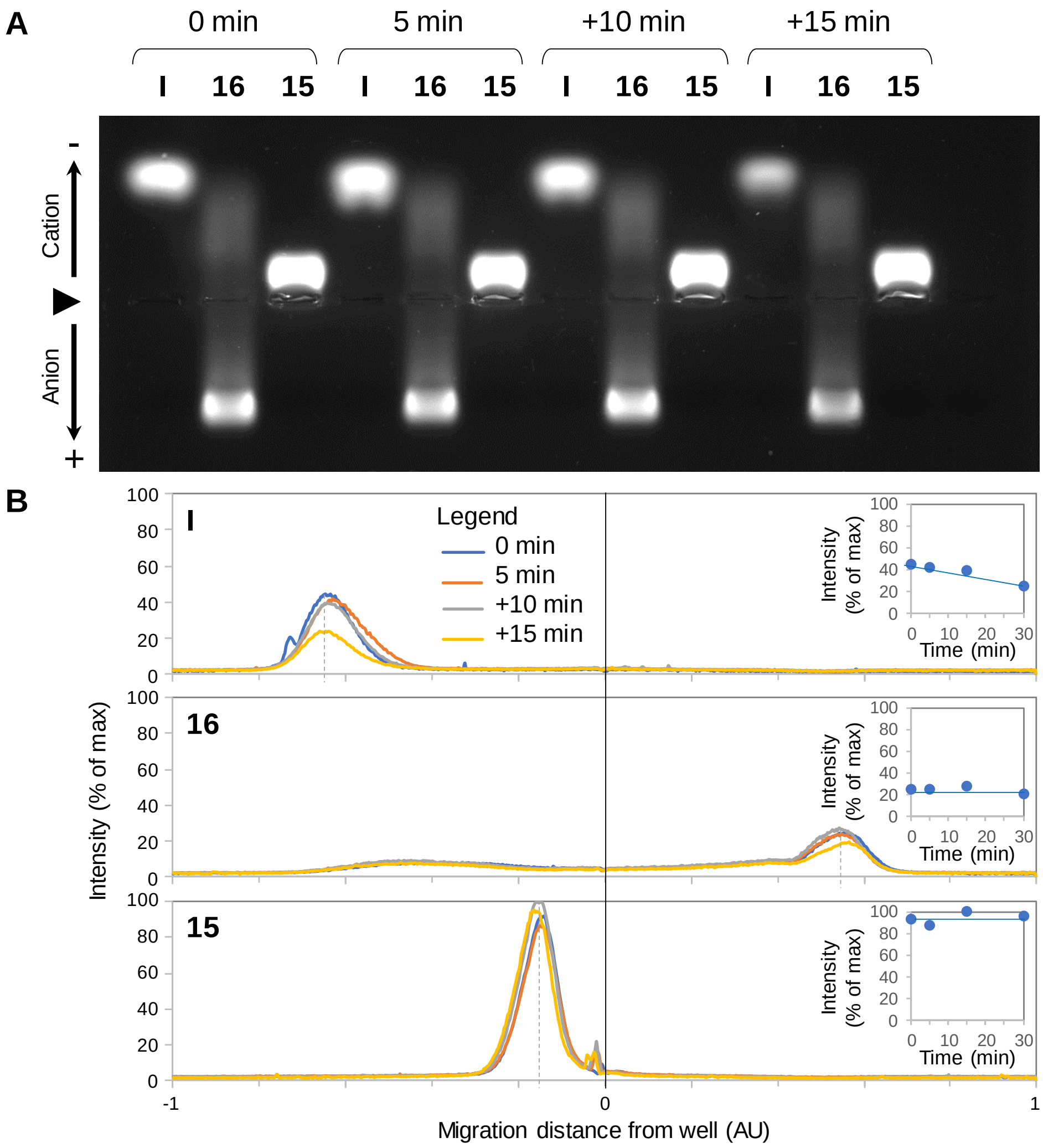

FIGURE S8 | Assessing the photostability of aPDT-polymers upon exposure to light for defined durations. Picture of the gel **(panel A)** and quantification of histogram profile of the signal for every compound **(panel B)**. aPDT-polymers with net negative (**16**) or positive (**15**) charge were exposed to blue light and sampled at defined time intervals (5, 15 [5+10], and 30 [5+10+15] minutes). Compound **I** was included as a benchmark. In panel B, the inserts illustrate the relative peak intensity over time for each compound. The arrowhead marks the wells where samples were loaded (2 µg of **I** and 20 µg of aPDT-polymers), and vertical arrows indicate the electrophoretic migration path, reflecting whether the reagents carried a net cationic or anionic charge. AU, arbitrary unit.

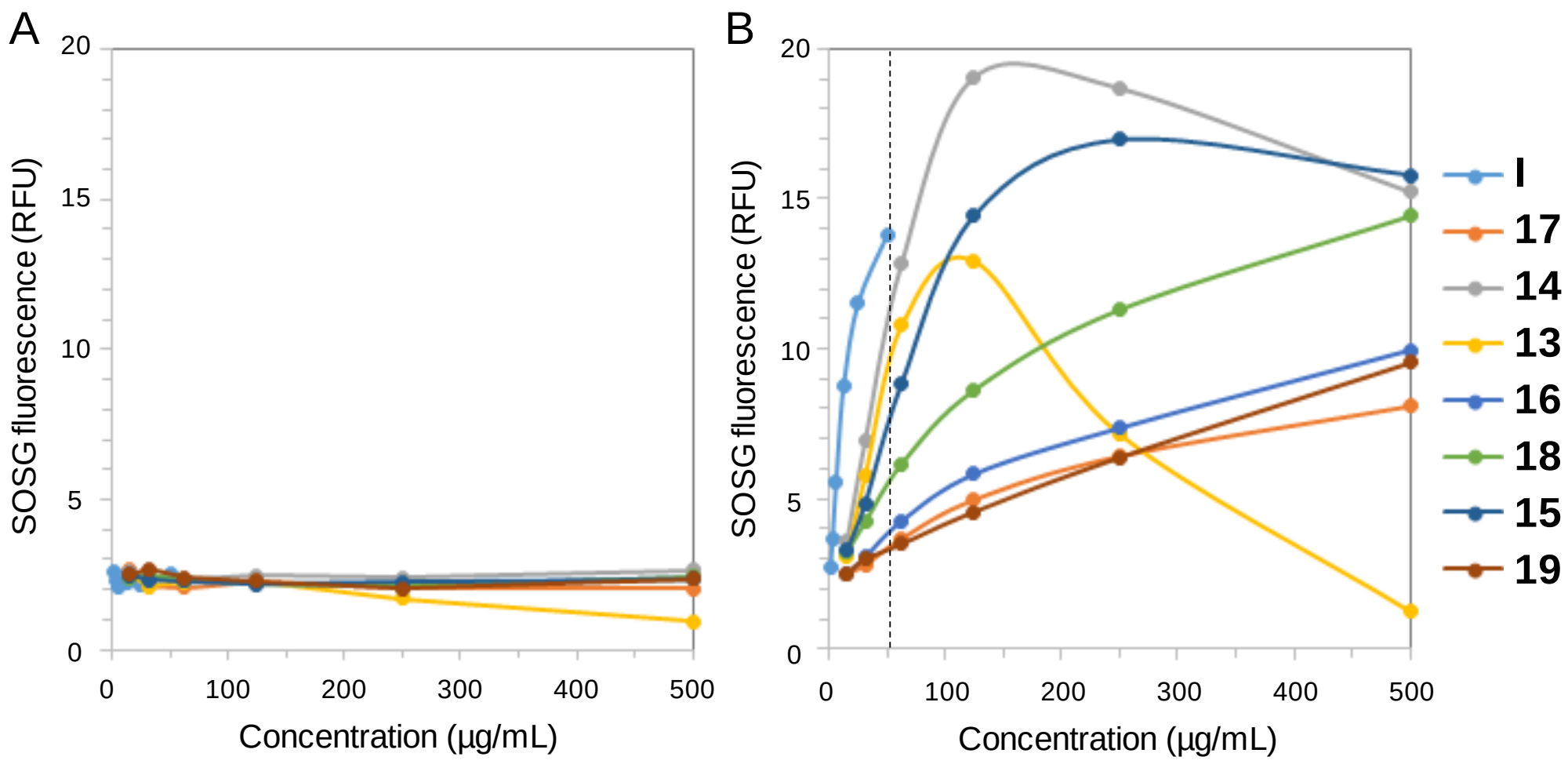

FIGURE S9 | Detection of singlet oxygen production by compound **I** and aPDT-polymers: Fluorescence intensity of the SOSG dye is shown before **(panel A)** and after **(panel B)** light exposure. Samples were tested in water at concentrations up to 50 µg/mL for compound **I** and up to 500 µg/mL for aPDT-polymers. In panel B, the dashed vertical line marks the 50 µg/mL threshold, beyond which the dose-response relationship deviates from linearity for almost all reagents. RFU: relative fluorescence units.

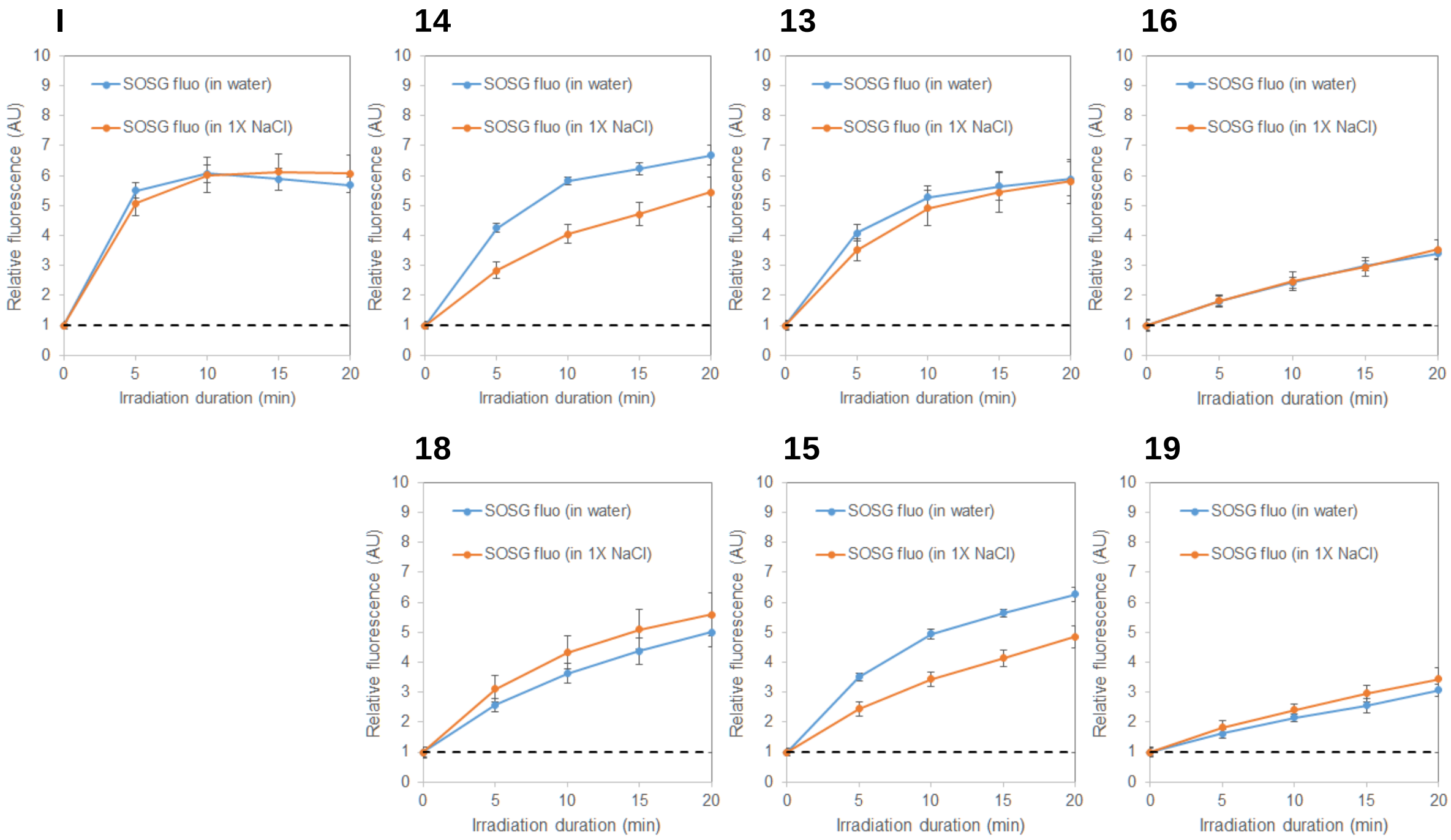

FIGURE S10 | Kinetics of singlet oxygen production by compound **I** and aPDT-polymers: effect of irradiation duration either in water or in 0.9% NaCl solution. All reagents were tested at a concentration of 50 µg/mL. Measurements were recorded following four successive 5-minute irradiation cycles. The dashed line indicates the baseline signal measured prior to light exposure. Data are presented as mean ± standard deviation (n = 3).

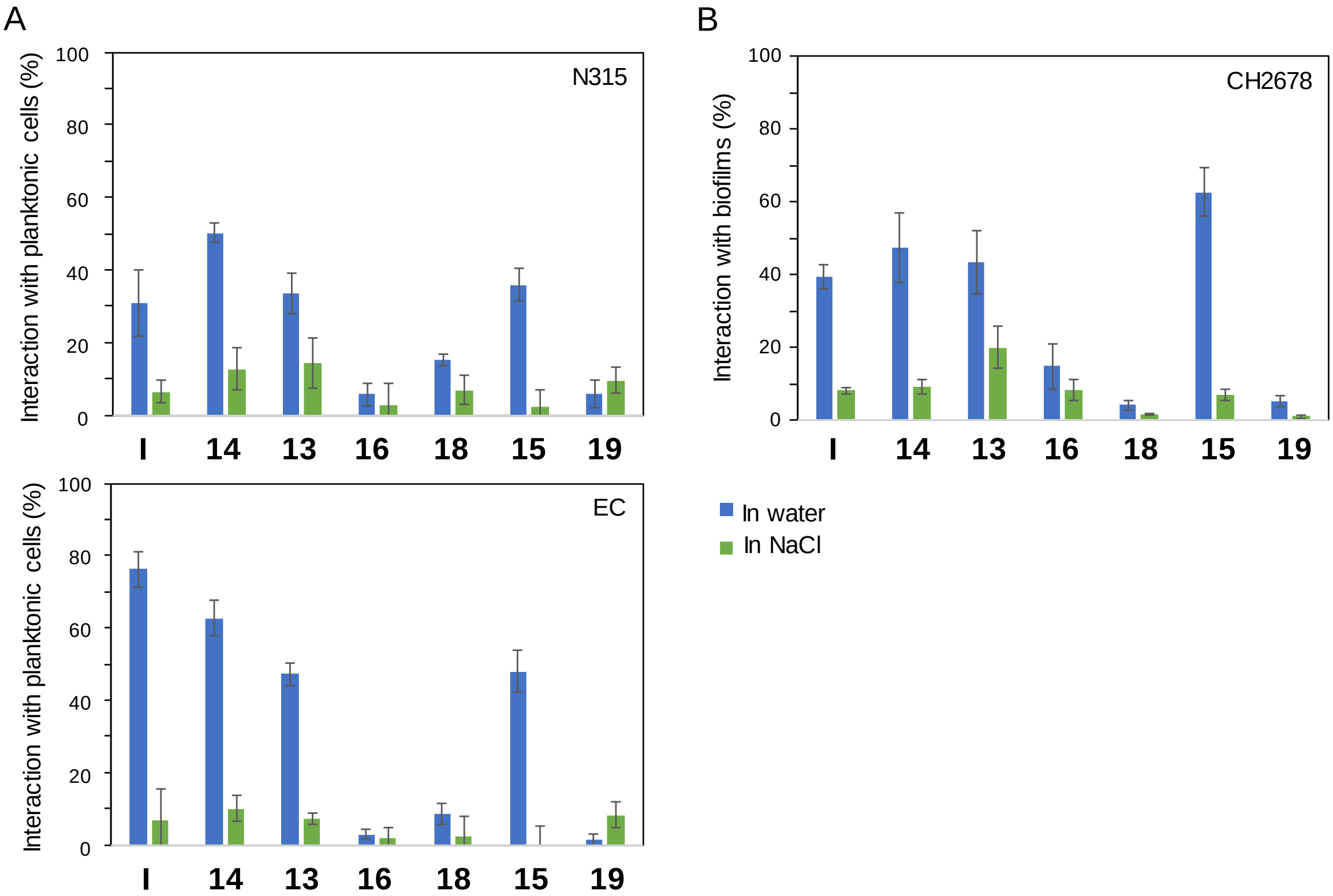

FIGURE S11 | Interaction of compound **I** and PDT-polymers with bacteria, either as planktonic cells **(panel A)** or embedded in biofilms **(panel B)**, following incubation in water or in 1× NaCl solution. Values > 0% indicate association. The results shown correspond to SA N315, EC DH5alpha, and PA CH2678. Data are presented as mean ± SD (n = 3).

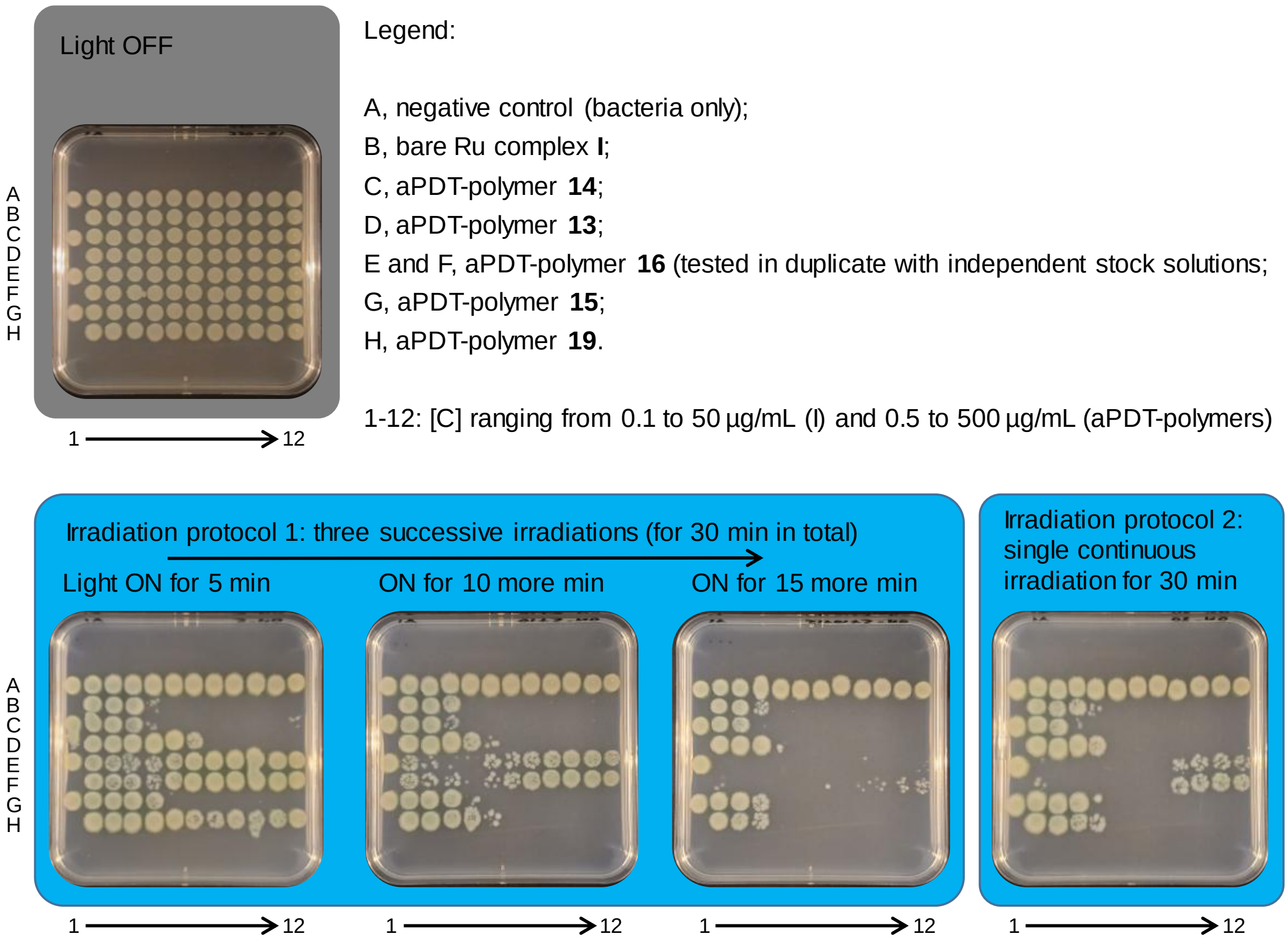

FIGURE S12 | Antimicrobial photodynamic therapy using **I** and aPDT-polymers against planktonic *P. aeruginosa* PA19 cells, focusing on light duration and irradiation protocols. Bacterial survival before and after exposure was assessed under either discontinuous or continuous irradiation. Compound **I** and aPDT-polymers were tested in aqueous solutions at concentrations [C] of 0.1-50 µg/mL and 0.5-500 µg/mL, respectively. At selected time points, 5 µL from each condition was plated on LB agar. After incubation at 37 °C for several hours, survival was determined by colony formation. Compound **16** was tested twice using independent preparations.

Notably, **16** yielded unexpected yet reproducible outcomes.

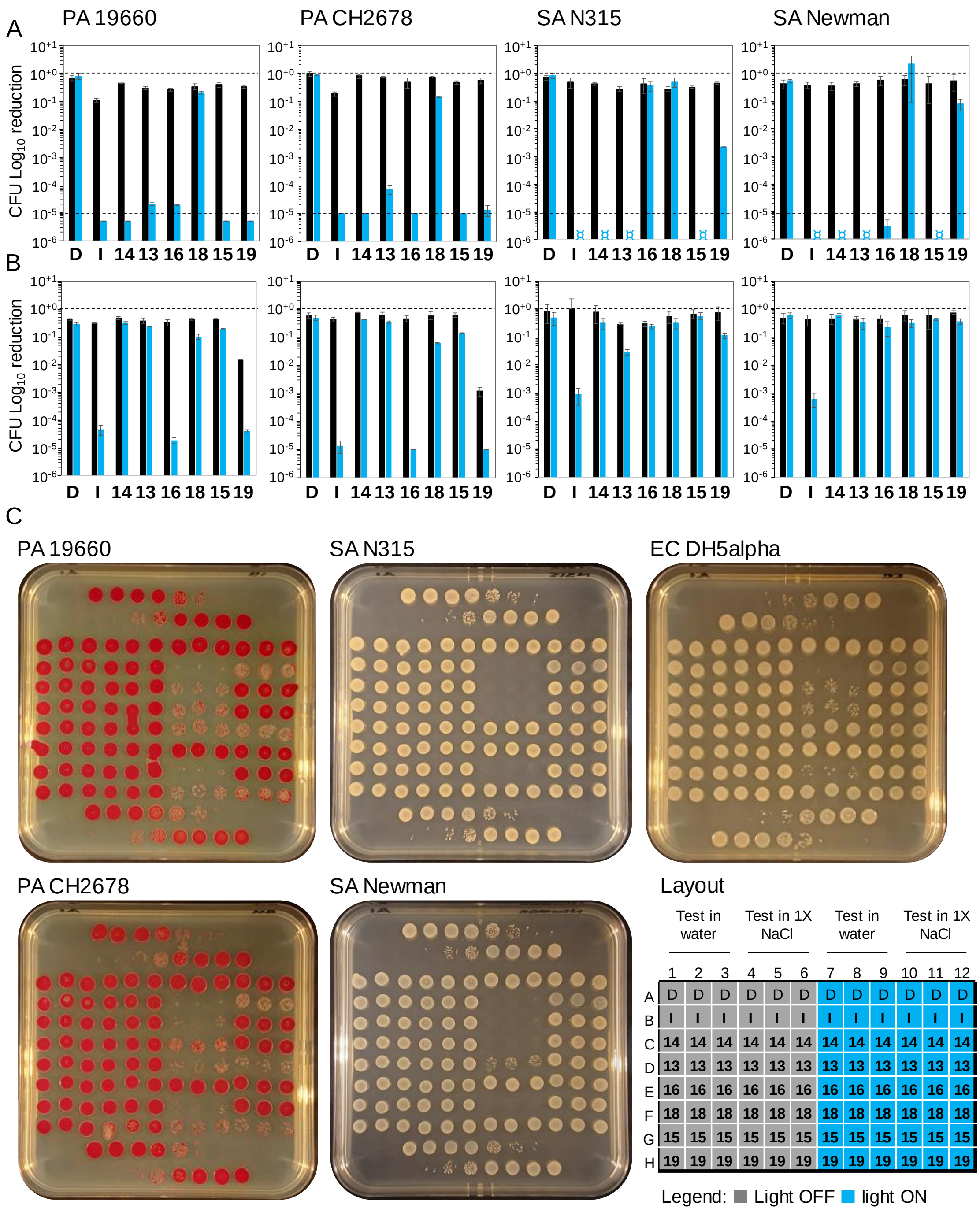

FIGURE S13 | Load reduction (in CFU Log_{10}) of planktonic bacteria treated with **I** or aPDT-polymers either kept in the dark (OFF) or exposed to light (ON): comparison of the results obtained for tests done in water **(panel A)** or in 0.9% NaCl **(panel B)**. The bacteria tested were Gram-negative *P. aeruginosa* (PA19 and CH2678) and Gram-positive *S. aureus* (N315 and Newman). The data correspond to mean +/- SD with n=3. The bottom dashed line refers to the limit of detection. The symbol ¤ is used to indicate reductions that fall below the detection threshold. (**Panel C**) Bacterial reduction treated with the bare complex **I** and the various aPDT-polymers and exposed to blue LED light (ON) or protected from that light (OFF): comparison of the results obtained for tests done in water or in 0.9% NaCl. Bacterial growth was used to estimate the log_{10} reduction, taking into account the parallel growth of bacterial dilutions (top and bottom parts of each agar plate). *E. coli* (DH5alpha) was additionally tested here. For both *P. aeruginosa* strains, the LB medium was supplemented with TTC to enable better visualization of bacterial growth. **17** was not tested here.

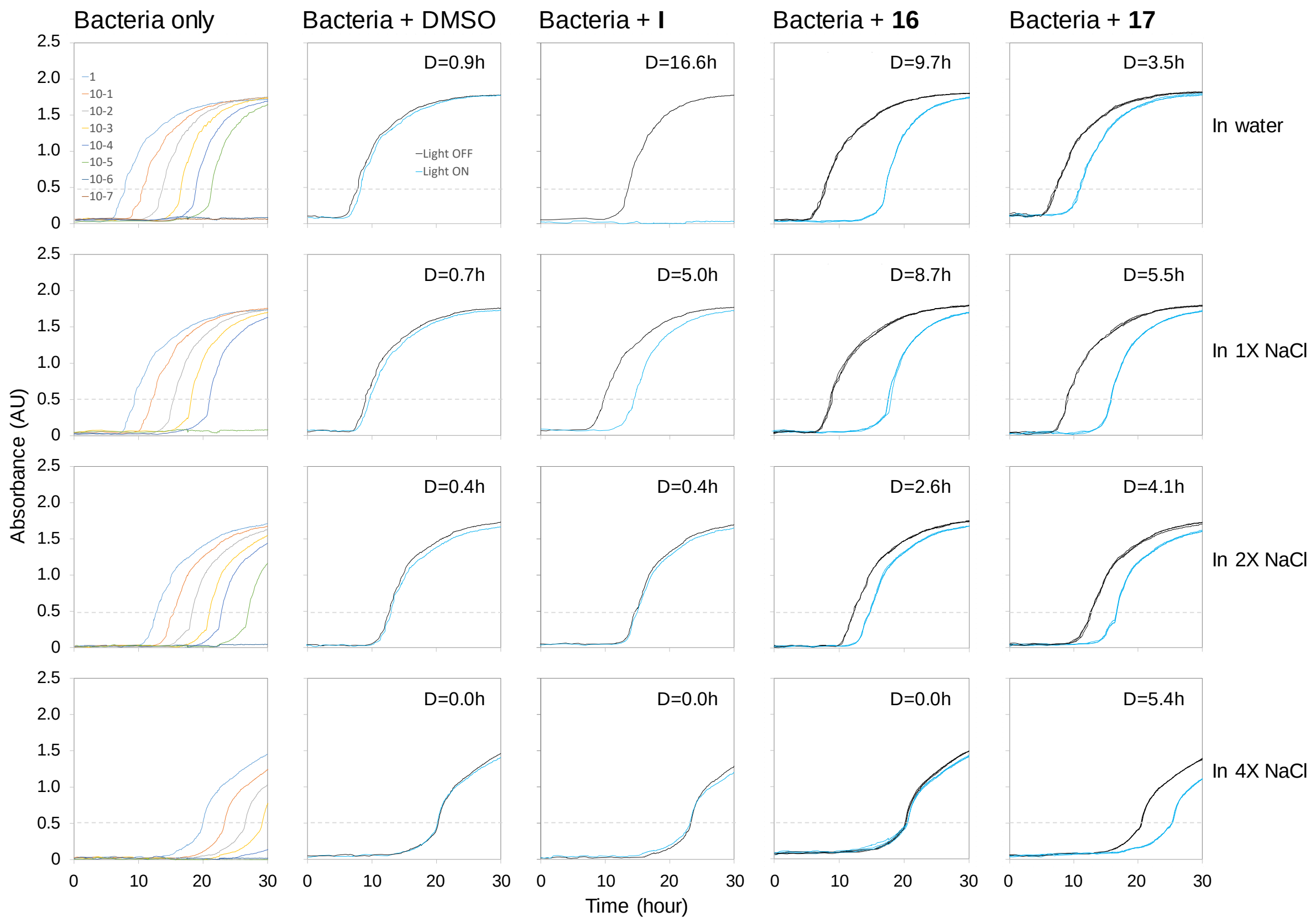

FIGURE S14 | Impact of NaCl concentration on the aPDT activity against planktonic PA19660 cells for compounds **I**, **16**, and **17**. Compound **I** was tested at 10 µg/mL, while **16** and **17** were tested at 100 µg/mL. DMSO (0.2%) and untreated bacteria served as parallel controls. The delay (D) represents the growth retardation (expressed in hours and calculated as the time required for the culture to exceed an absorbance of 0.5) observed between illuminated and non-illuminated conditions for each treatment. This delay can be translated into an estimated bacterial load reduction by referencing the growth kinetics of the corresponding bacterial dilutions grown in parallel.

Experiments were performed at sub-inhibitory concentrations to reveal how NaCl levels influence the ability of each compound to exert aPDT. The data highlight a strong sensitivity of compound **I** to salinity, an intermediate sensitivity for **16**, and a marked resistance of **17**, whose aPDT efficacy remains largely unaffected by increasing NaCl concentrations.

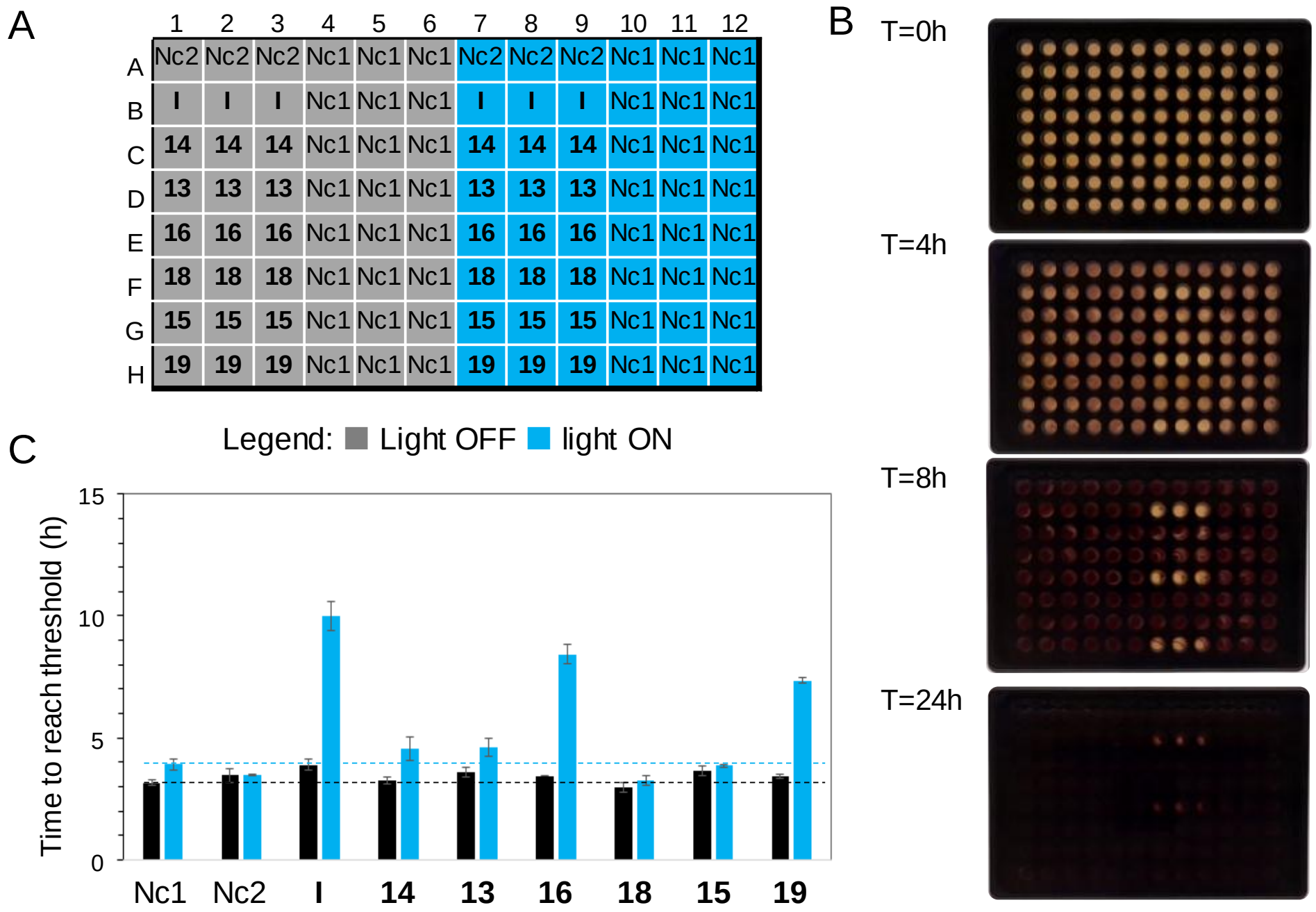

FIGURE S15 | Metabolic activity of *P. aeruginosa* CH2678 biofilms treated with **I** or aPDT-polymers and kept in the dark (OFF) or exposed to light (ON): Layout of the well plates **(panel A)**; images of biofilms after treatment at different incubation times **(panel B)**; and graphical representation of the calculated time required to detect metabolic activity (red shift) **(panel C)**. Compound **I** and aPDT-polymers were tested at 50 and 500 µg/mL, respectively. The dashed lines indicate the level determined for biofilm alone (not exposed to any compound). Nc1: biofilm alone; Nc2: biofilm with DMSO (solvent required for **I**). The data correspond to mean +/- SD with n=3.

Cell viability:

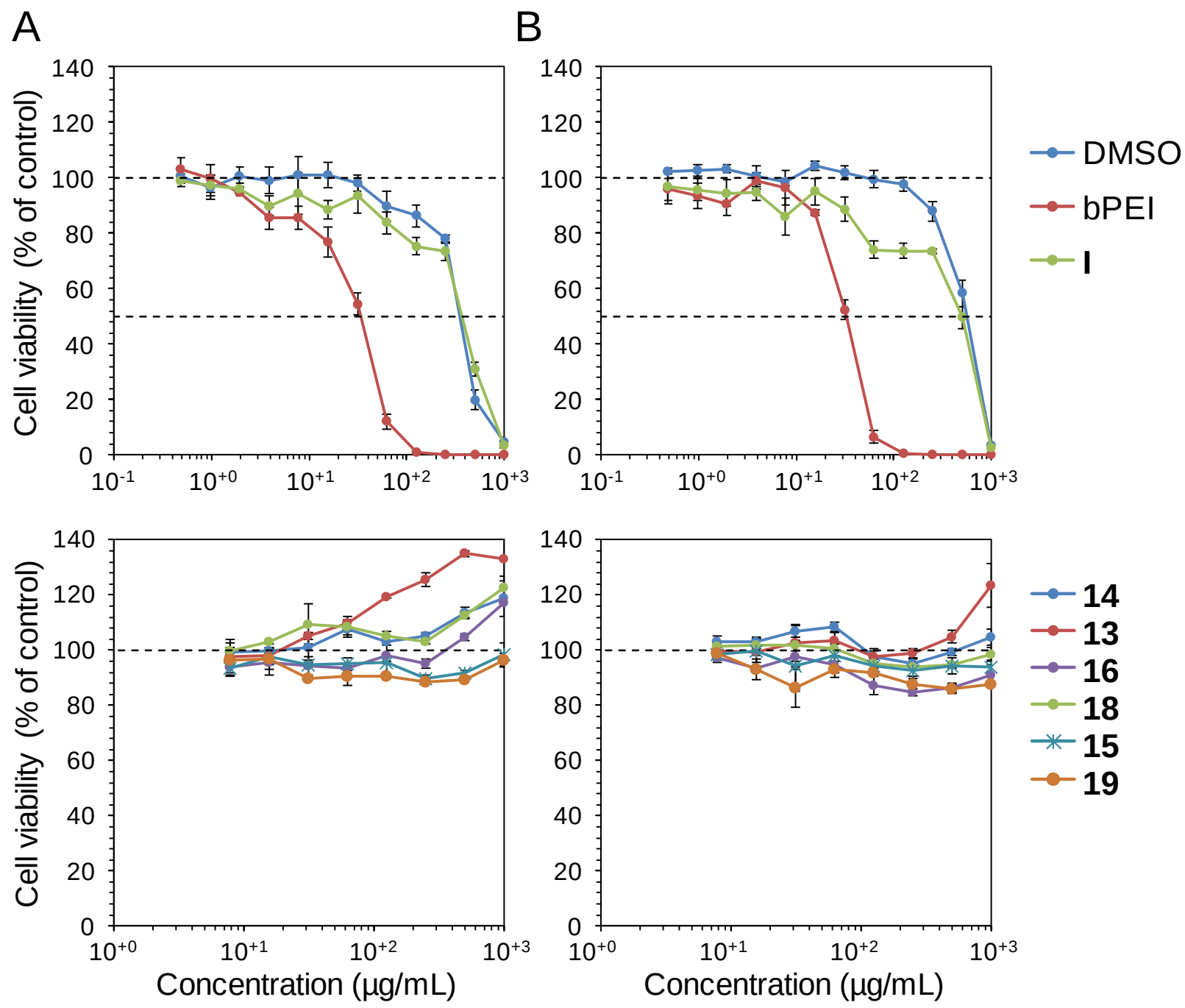

Cytotoxicity:

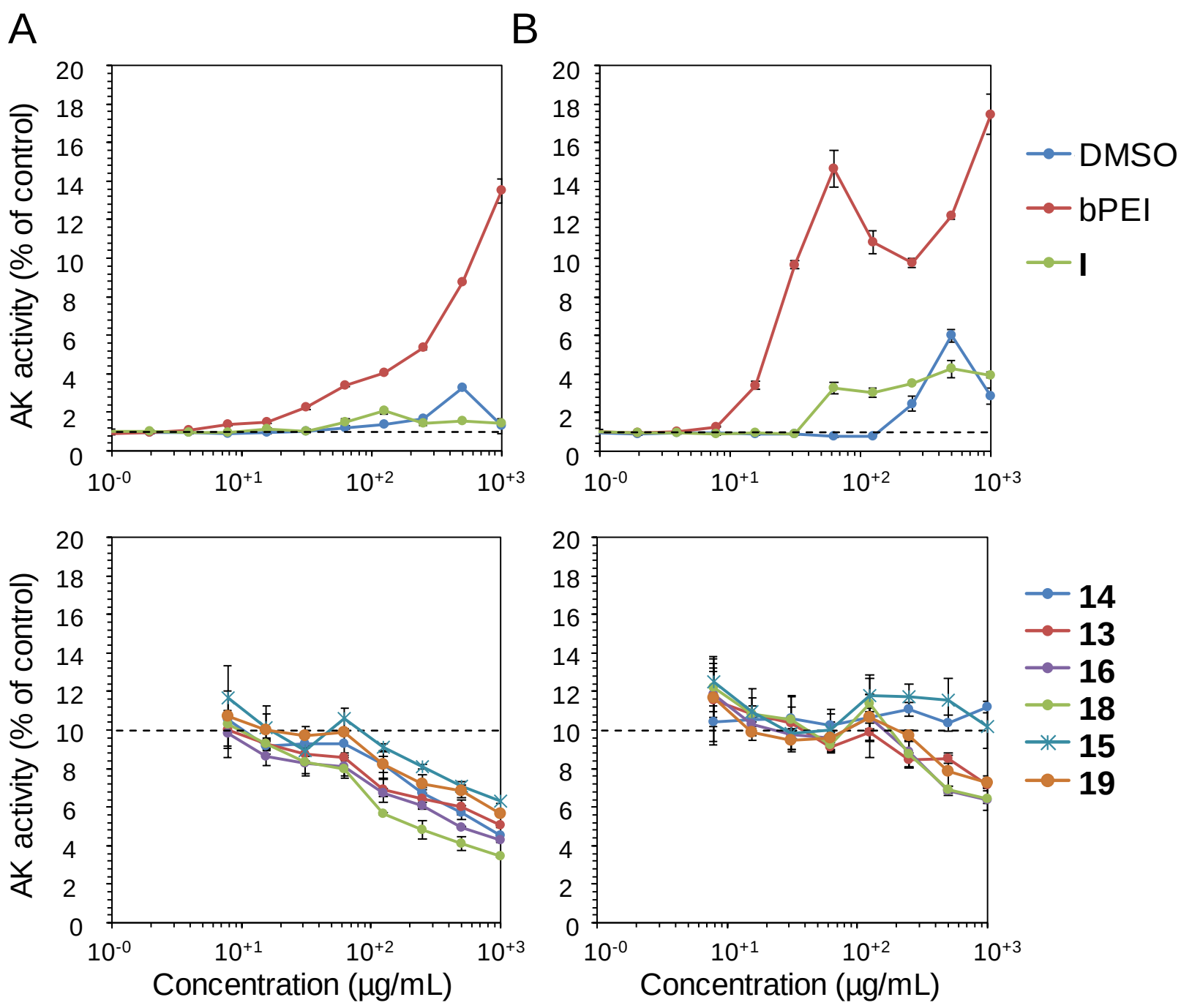

FIGURE S16 | Cell viability and cytotoxicity towards eukaryotic epithelial cells following 24-hour exposure to compound **I** or aPDT-polymers (in the absence of blue LED light). Viability was assessed based on intracellular ATP content, in 16HBE **(panel A)** or A549 **(panel B)** cells. DMSO (solvent required for **I**) and branched polyethyleneimine (bPEI) were included as control treatments. Dashed lines indicate reference levels for 100% and 50% cell viability. Cytotoxic effects were assessed by quantifying adenylate kinase (AK) release from damaged cells, either 16HBE **(panel A)** or A549 **(panel B)**. DMSO (solvent for **I**) and branched polyethyleneimine (bPEI) were included as control treatments. Dashed lines indicate the reference level corresponding to 1 unit of AK activity. As a reference, 50 µg/mL of **I** correspond to 1% DMSO. Data are presented as mean ± SD (n = 3).

S7 | Supplementary References

[1] K. Summer, J. Browne, M. Hollanders, K. Benkendorff, “Out of control: The need for standardised solvent approaches and data reporting in antibiofilm assays incorporating dimethyl-sulfoxide (DMSO)” *Biofilm* **2022**, *4*, 100081.
[2] T. Le Gall, G. Lemercier, S. Chevreux, K.-S. Tücking, J. Ravel, F. Thétiot, U. Jonas, H. Schönherr, T. Montier, “Ruthenium(II) Polypyridyl Complexes as Photosensitizers for Antibacterial Photodynamic Therapy: A Structure-Activity Study on Clinical Bacterial Strains” *ChemMedChem* **2018**, *13*, 2229–2239.
[3] R. Youf, A. Nasir, M. Müller, F. Thétiot, T. Haute, R. Ghanem, U. Jonas, H. Schönherr, G. Lemercier, T. Montier, T. Le Gall, “Ruthenium(II) Polypyridyl Complexes for Antimicrobial Photodynamic Therapy: Prospects for Application in Cystic Fibrosis Lung Airways” *Pharmaceutics* **2022**, *14*, 1664.
[4] R. Youf, R. Ghanem, A. Nasir, G. Lemercier, T. Montier, T. Le Gall, “Impact of mucus and biofilm on antimicrobial photodynamic therapy: Evaluation using Ruthenium(II) complexes” *Biofilm* **2023**, *5*, 100113.
[5] T. Baba, T. Bae, O. Schneewind, F. Takeuchi, K. Hiramatsu, “Genome sequence of Staphylococcus aureus strain Newman and comparative analysis of staphylococcal genomes: polymorphism and evolution of two major pathogenicity islands” *J Bacteriol* **2008**, *190*, 300–310.
[6] M. Kuroda, T. Ohta, I. Uchiyama, T. Baba, H. Yuzawa, I. Kobayashi, L. Cui, A. Oguchi, K. Aoki, Y. Nagai, J. Lian, T. Ito, M. Kanamori, H. Matsumaru, A. Maruyama, H. Murakami, A. Hosoyama, Y. Mizutani-Ui, N. K. Takahashi, T. Sawano, R. Inoue, C. Kaito, K. Sekimizu, H. Hirakawa, S. Kuhara, S. Goto, J. Yabuzaki, M. Kanehisa, A. Yamashita, K. Oshima, K. Furuya, C. Yoshino, T. Shiba, M. Hattori, N. Ogasawara, H. Hayashi, K. Hiramatsu, “Whole genome sequencing of meticillin-resistant Staphylococcus aureus” *Lancet* **2001**, *357*, 1225–1240.
[7] A. L. Cozens, M. J. Yezzi, K. Kunzelmann, T. Ohrui, L. Chin, K. Eng, W. E. Finkbeiner, J. H. Widdicombe, D. C. Gruenert, “CFTR expression and chloride secretion in polarized immortal human bronchial epithelial cells” *Am J Respir Cell Mol Biol* **1994**, *10*, 38–47.
